# A method for the further assembly of targeted unigenes in a transcriptome after assembly by Trinity

**DOI:** 10.3389/fpls.2015.00843

**Published:** 2015-10-14

**Authors:** Xinlong Xiao, Jinbiao Ma, Yufang Sun, Yinan Yao

**Affiliations:** ^1^Key Laboratory of Biogeography and Bioresource in Arid Land, Xinjiang Institute of Ecology and Geography, Chinese Academy of ScienceUrumqi, China; ^2^University of Chinese Academy of SciencesBeijing, China

**Keywords:** RNA-seq, unigene assembly, *Salicornia europaea*, nitrate transporter gene, phosphate transporter gene, expression pattern, RPKM

## Abstract

RNA-sequencing has been widely used to obtain high throughput transcriptome sequences in various species, but the assembly of a full set of complete transcripts is still a significant challenge. Judging by the number of expected transcripts and assembled unigenes in a transcriptome library, we believe that some unigenes could be reassembled. In this study, using the nitrate transporter (*NRT*) gene family and phosphate transporter (*PHT*) gene family in *Salicornia europaea* as examples, we introduced an approach to further assemble unigenes found in transcriptome libraries which had been previously generated by Trinity. To find the unigenes of a particular transcript that contained gaps, we respectively selected 16 *NRT* candidate unigene pairs and 12 *PHT* candidate unigene pairs for which the two unigenes had the same annotations, the same expression patterns among various RNA-seq samples, and different positions of the proteins coded as mapped to a reference protein. To fill a gap between the two unigenes, PCR was performed using primers that mapped to the two unigenes and the PCR products were sequenced, which demonstrated that 5 unigene pairs of *NRT* and 3 unigene pairs of *PHT* could be reassembled when the gaps were filled using the corresponding PCR product sequences. This fast and simple method will reduce the redundancy of targeted unigenes and allow acquisition of complete coding sequences (CDS).

## Introduction

Whole transcriptome sequencing (RNA-seq) with next-generation sequencing (NGS) technology has been used to uncover the complex landscape and dynamics of transcriptomes in various plant species since the success of the massively parallel pyrosequencing of the Arabidopsis transcriptome (Weber et al., 2007). Because of the great depth of sequencing it allows, RNA-seq can produce a nearly complete profile of a transcriptome, even including rare transcripts. Furthermore, RNA-seq has many advantages, such as the base-pair-level resolution, the large range of expression level, and *de novo* annotation (Martin and Wang, 2011). Compared with the high cost of genome sequencing, using RNA-seq, ordinary laboratories can produce transcriptome sequences for species of interest (Hamilton and Buell, 2012), and more than 50 different plant species have been sequenced using this technology (Schliesky et al., 2012).

There are two principal strategies for transcriptome assembly: *de novo* assembly or assembly based on a reference genome. A reference genome is not available for most species, therefore, *de novo* assembly of the transcriptome is commonly used for non-model species (Zhao et al., 2011). Several applications have been designed for *de novo* assembly, such as Multiple-k (Surget-Groba and Montoya-Burgos, 2010), Rnnotator (Martin et al., 2010), Trans-ABySS (Robertson et al., 2010), Oases (Schulz et al., 2012), and Trinity (Grabherr et al., 2011). However, regardless of which assembly application is employed, it is still difficult to reconstruct a comprehensive set of full-length transcripts, because RNA-sequence reads are often quite short and many informatics problems exist (Metzker, 2010; Martin and Wang, 2011). A Fermi estimate indicates that approximately 15,000 transcripts possibly exist in a plant transcriptome, and conceivably nearly twice as many, but not greater than 10-fold more (Schliesky et al., 2012). In fact, the number of unigenes (i.e., the final assembled sequence) often exceeds the number of expected transcripts (including isoforms), not to mention the large amount of discarded short unigenes between 0 and 200 bp in length (Schliesky et al., 2012). Therefore, some unigenes that represent the same transcript but contain a gap so that they cannot be assembled might exist in some transcriptome libraries. Finding and assembling these unigenes will allow the acquisition of full-length transcripts and further comprehensive study of the genome.

**Figure 1:**
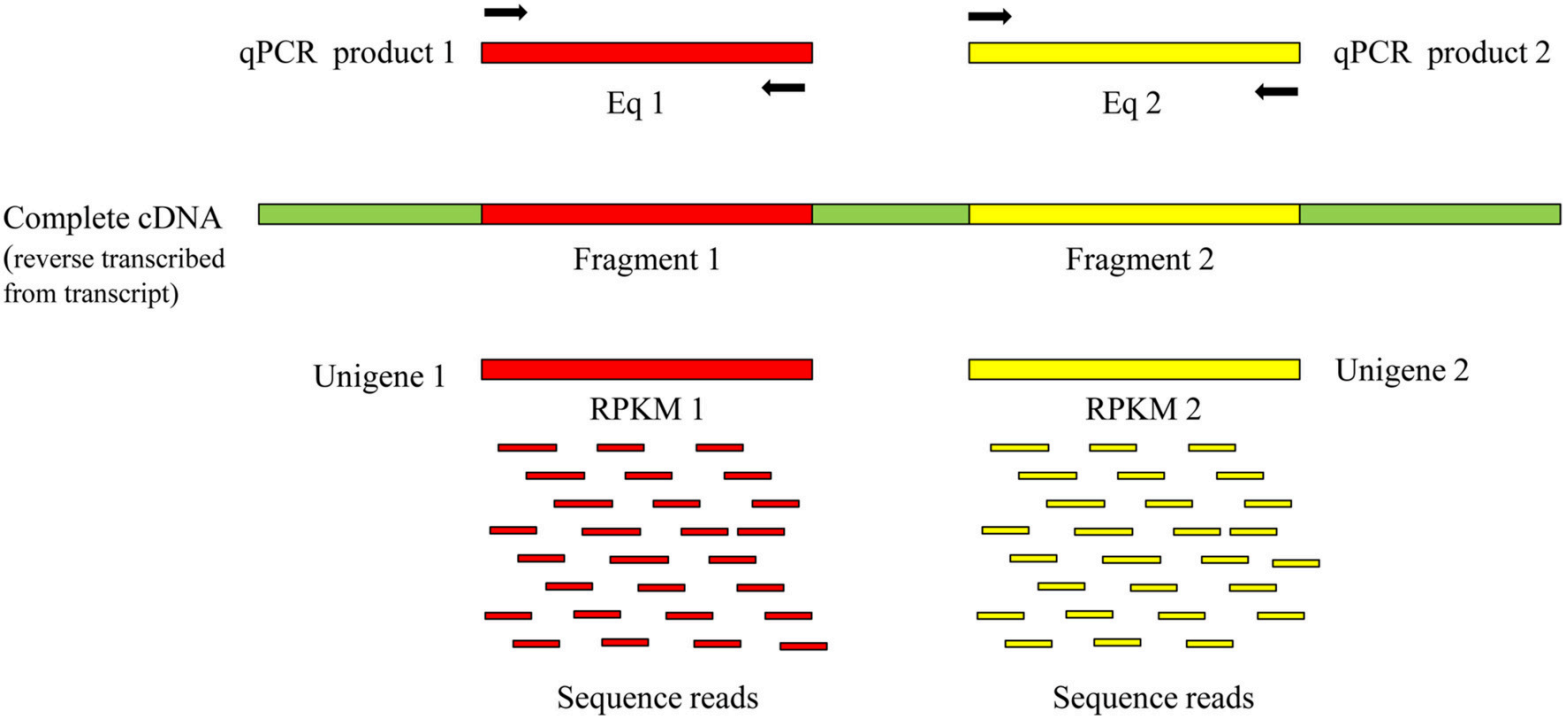
**Schematic diagram for detecting the expression of a transcript on different fragments by RT-qPCR and RNA-seq**. Eq. expression quantity. Black arrows represent primer pairs of RT-qPCR.

We believe that the same quantities of such unigenes were expressed in identical RNA-seq samples. RPKM (reads per kilobase of exon model per million mapped reads) is often used to quantify transcript levels in RNA-seq samples (Mortazavi et al., 2008). As shown in the schematic diagram in Figure 1, we assumed that two fragments comprised a transcript and that RT-qPCR and RNA-seq were respectively used to determine the expression of the transcript. Although, the qPCR primer pairs were designed to match different sequences in the transcript, the value obtained from Eq1 should be equal to that from Eq2. Similarly, if unigene1 and unigene2 both represent the same transcript, their expression levels as indicated by RPKM reflect the expression level of the transcript and in theory, RPKM1 should be equal to RPKM2. This should be a feasible method to distinguish the unigenes that represent the same transcript based on the expression patterns among various RNA-seq samples.

*Salicornia europaea*, one of the most salt-tolerant halophytes, can tolerate as much as 1000 mM NaCl in the soil (Flowers and Colmer, 2008; Lv et al., 2011). It also has a high capacity for the absorption of inorganic nitrogen, such as ${\text{NO}}_{3}^{-}$ and ${\text{NH}}_{4}^{+}$ (Webb et al., 2012). Three types of nitrate transporters, NRT1, NRT2, and NRT3 (NAR2) that aid in the transport of the principal inorganic nitrogen source ${\text{NO}}_{3}^{-}$ have been identified in higher plants (Forde, 2000; Chen et al., 2008). In Arabidopsis, there are 53 *NRT1* genes, 7 *NRT2* genes and 2 *NRT3* genes (Chapman and Miller, 2011; Tsay and Hsu, 2011). Some members of the NRT1 family are peptide transporters (PTR), therefore, NRT1 and PTR are usually classified into a single family known as NRT1/PTR (Tsay et al., 2007). For the absorption of inorganic phosphate (Pi), four types of phosphate transporters, PHT1, PHT2, PHT3, and PHT4, have been identified in higher plants. And there are 9 *PHT1* genes, 1 *PHT2* genes, 3 *PHT3* genes and 6 *PHT4* genes in Arabidopsis (Liu et al., 2011).

**Figure 2:**
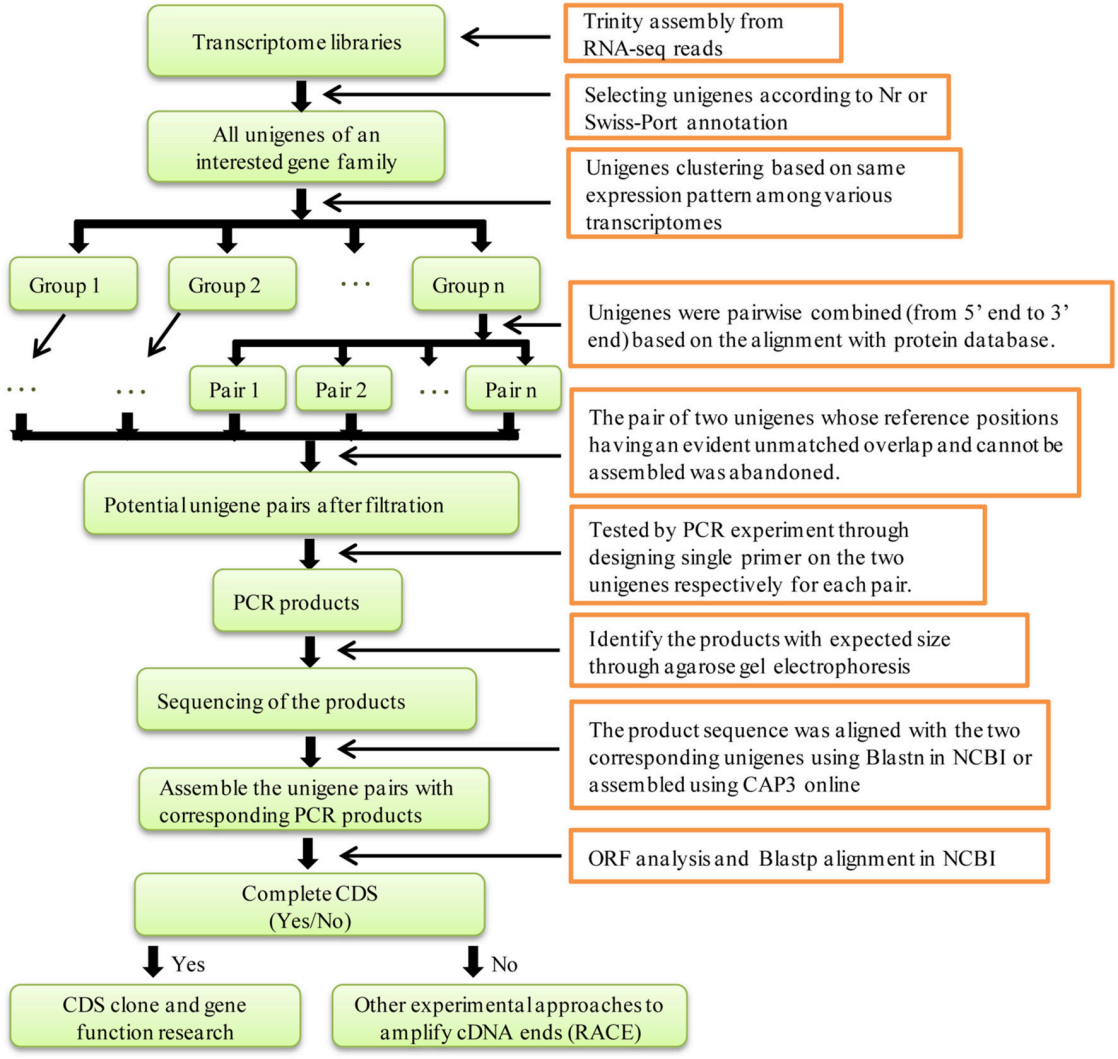
**Flow chart for distinguishing and assembling potential unigenes for a transcript in a transcriptome library**. The transcriptome library contained all of the unigenes which were assembled by Trinity from RNA-seq reads.

In a previous study, we subjected four samples of *S. europaea* to RNA-seq via Illumina HiSeq 2000 and assembled the sequence reads *de novo* using Trinity (Ma et al., 2013). There were 118 *NRT* unigenes (including 75 *NRT1/PTR*, 37 *NRT2*, and 6 *NRT3*) and 47 *PHT* unigenes (including 24 *PHT1*, 2 *PHT2*, 12 *PHT3*, and 9 *PHT4*) in the various transcriptome libraries. It is possible that some unigenes could be reassembled because Xiao et al. found that two unigenes for an ammonium transporter (AMT) in the transcriptome libraries were assembled through experimental validation (Xiao et al., 2014). However, a global analysis of all of the unigenes in the gene family with respect to the assembly of the *AMT* unigenes considered in this study was not performed and the expression pattern of the unigenes was not unambiguously classified. Therefore, in this study, the method was improved to systematically find and assemble putative unigenes that represented single transcripts for the entire *NRT* and *PHT* family in *S. europaea*. The steps of this method are shown in Figure 2.

## Materials and methods

### RNA-seq of *S. europaea* samples

In a previous study, we RNA-sequenced four *S. europaea* samples (shoot and root, under 200 mM NaCl-treated or NaCl-free conditions, respectively) using the Illumina HiSeq 2000 (Ma et al., 2013). Numerous unigenes for each transcriptome library were generated and these unigenes could not be extended on either end as assembled by Trinity. A library named Unigene-All was clustered among the four transcriptome libraries using an application TGICL to obtain non-redundant unigenes as long as possible (Pertea et al., 2003). Functional annotation of the unigenes was accomplished using Blastx alignment (*e* < 0.00001) between the unigenes and protein databases including Nr, Swiss-Prot, KEGG, and COG. The proteins that with the highest sequence similarity were taken as the annotation for the unigenes. The RPKM method (Mortazavi et al., 2008), which normalizes for the effect of the gene lengths and sequencing levels in the calculation of gene expression was used to determine gene expression level under four conditions.

### Collection and clustering of *NRT* and *PHT* unigenes

In this study, the *NRT* and *PHT* unigenes were collected from Unigene-All transcriptome library based on the Nr or Swiss-Prot annotation. The information for each unigene is comprised of the sequence length, the annotation and the RPKM values for corresponding libraries. The software Cluster 3.0 was used to group unigenes by hierarchical clustering method with log transform data according to the expression level in four libraries (de Hoon et al., 2004). The clustering results were viewed using Java TreeView (Strickler et al., 2012). Unigenes that had the same expression pattern were classified into a group.

### Potential unigenes belonging to a transcript after filtration

The unigenes in each group were located in sequences from *Arabidopsis thaliana* using Blastx in GenBank to estimate the position of the corresponding transcript based on the alignment with a reference protein, for example, locating the 5′end but not the 3′ end, or locating the 3′end but not the 5′ end, or failing to locate either the 5′or the 3′ end. Pairwise analysis was used for the unigenes in each group and combinations in which two unigenes had an evident unmatched overlap and could not be assembled were not considered. However, combinations of unigenes that contained a gap or had not an evidence overlap were retained for further analysis.

### Verified by PCR and sequencing of products

The selected pairs of unigenes were subjected to PCR amplification. We assumed that the two unigenes came from a single transcript and were joined together based on the position of the 5′ or the 3′ end. Forward and reverse primers were respectively designed based on the two corresponding unigenes, and at least a 200 bp overlap between the hypothetical PCR products and each unigene was ensured.

The total RNA of *S. europaea* was extracted using an RNeasy Mini Kit (Qiagen) and cDNA synthesis was accomplished with a Reversed Transcription Reagent Kit (TaKaRa) following the manufacturer's instructions. The PCR reaction was performed in Bio-Rad PCR Instrument in triplicate. The PCR reaction mixture contained 10 μL of high fidelity Es Taq mix (CWBIO), 2 μL of a 10-fold dilution of cDNA, 0.8 μL of each of the forward and reverse primers (10 μM), and 6.4 μL of PCR-grade water in a final volume of 20 μL. The following reaction conditions were used: 2 min at 94°C, 30 cycles of 30 s at 94°C and 30 s at the corresponding annealing temperature and 1 min at 72°C, and a final 5 min at 72°C. PCR products were visualized with 1.2% agarose gel electrophoresis and the expected bands were collected using a Gel Extraction Kit (Omega). The products were directly sequenced (BGI, Beijing) or sequenced after insertion into a pMD20-T vector (TaKaRa) and cloning in *Escherichia Coli*. The PCR sequences were aligned with the two corresponding unigenes using Blastn in NCBI and assembled based on matching overlaps using CAP3 (http://doua.prabi.fr/software/cap3) (Huang and Madan, 1999).

### Sequence analysis of the assembled unigenes

The assembled sequences were performed Blastx alignment in nr database of *Arabidopsis thaliana* to determine their annotation of coding protein. Open reading frames (ORF) were analyzed on ORF Finder (http://www.ncbi.nlm.nih.gov/gorf/gorf.html) to find the biggest one and whether that had a termination codon forward the initiation codon in 5′ end. The deduced amino acid sequences were searched against the nr protein database using Blastp in GenBank to judge whether it was a complete CDS through comparing the number of amino acid with closely related organisms.

### Expression analysis of two fragments of a transcript

To prove that the unigenes that comprise a single transcript had same expression levels, we randomly chose two pairs of unigenes (comprising to a transcript) and quantified the gene expression via real-time qPCR. *S. europaea* was exposed to NaCl stress or NaCl-free conditions, and then the shoots and roots were collected in triplicate. The total RNA was extracted using an RNeasy Mini Kit (Qiagen) and contaminating DNA was eliminated using an RNase-free DNase kit (Qiagen). An aliquot of 1 μg of the total RNA was used for cDNA synthesis at a final volume of 20 μL using a Reverse Transcription Reagent Kit (TaKaRa) following the manufacturer's instructions. Specific primer pairs for various unigenes were designed online using NCBI Primer-BLAST (Ye et al., 2012). Two reference genes (UBC and CAC) were used as an internal control for RT-qPCR under NaCl stress (Xiao et al., 2015a). RT-qPCR was performed in 96-well optical plates with a CFX96 Real-Time PCR Detection System (Bio-Rad, USA) using SYBR real-time PCR premixture (BioTeke, Beijing). The relative expression quantities of the unigenes were calculated by the 2^−ΔΔCt^ method (Livak and Schmittgen, 2001).

## Results

### *NRT* and *PHT* unigene sets

More than 140,000 high-quality unigenes in the Unigene-All transcriptome library were generated and had a mean size of 573 bp, which indicated that the transcriptomes were not completely assembled and that most unigenes did not contain a complete CDS (coding sequence) because of their short length and N50 value (Table 1). We found a total of 118 *NRT* unigenes, including 75 *NRT1* unigenes, 37 *NRT2* unigenes, and 6 *NRT3* unigenes per Nr annotation or Swiss-Prot annotation, in which only 10 *NRT1* unigenes, 1 *NRT2* unigene and 5 *NRT3* unigenes contained a complete CDS as indicated by an ORF analysis and Blastx alignment with other species in GenBank (Table 2A). *NRT3* transcripts were relatively well assembled because of their short coding region (approximately 200 aa), while most unigenes for *NRT1/PTR* and *NRT2* did not contain a complete CDS and could not be directly utilized for studies on gene function. Besides, 47 *PHT* unigenes were found, including 24 *PHT1* unigenes, 2 *PHT2* unigenes, 12 *PHT3* unigenes, and 9 *PHT4* unigenes, in which most did not contained a complete CDS except 3 *PHT1* unigenes, 1 *PHT3* unigenes, and 3 *PHT4* unigenes (Table 2B).

**Table 1:** **Quality assessment of Trinity assembly results**.

**Sample**	**Number**	**Total length (nt)**	**Mean length (nt)**	**N50**
Unigene_Se200S	101751	44551677	438	547
Unigene_SeCKS	97865	41490134	424	523
Unigene_Se200R	140086	56587612	404	482
Unigene_SeCKR	122728	52358894	427	528
Unigene_All	142721	81719801	573	780

*The unigenes with length under 200 nt were excluded. N50 represents the length for which 50% of the sequence in an assembly is in contigs of this size or larger*.

**Table 2A:** **Summary of ***NRT*** unigenes**.

**Unigene**	**Total number**	**Mean length (nt)**	**Number of unigenes with incomplete CDS**	**Number of *AtNRT* genes**	**Range of coding protein number (aa)**
*NRT1/PTR*	75	1023	65	53	521–636
*NRT2*	37	615	36	7	493–557
*NRT3*	6	989	1	2	209–210

**Table 2B:** **Summary of ***PHT*** unigenes**.

**Unigene**	**Total number**	**Mean length (nt)**	**Number of unigenes with incomplete CDS**	**Number of *AtPHT* genes**	**Range of coding protein number (aa)**
*PHT1*	24	832	21	9	516–542
*PHT2*	2	2543	2	1	613
*PHT3*	12	569	11	3	309–375
*PHT4*	9	1154	6	6	432–541

### Expression patterns clustering and unigenes grouping

To find unigenes that had the same expression pattern, we clustered the *NRT1, NRT2, NRT3, PHT1, PHT2, PHT3*, and *PHT4* unigenes based on gene expression under four conditions (200S, CKS, 200R, CKR). The unigenes, which are the nearest neighbors and indicate the shortest branch, have the most similar expression pattern. Based on the similarity of the unigenes expression, two to five unigenes were clustered into a group for further analysis, which were marked a box (Figures S1A, S1B). For the cluster of *NRT* unigenes, 18 *NRT1* groups and 9 *NRT2* groups were generated for further analysis (Table S1). There was no analysis for *NRT3*, because only 1 *NRT3* unigene (Unigene60534) did not have complete CDS and could not be assembled with other 5 *NRT3* unigenes that all had a complete CDS. For the cluster of *PHT* unigenes, 4 *PHT1* groups, 1 *PHT2* groups, 4 *PHT3* groups, and 2 *PHT4* groups were selected for next step (Table S2).

### Potential unigene pairs belonging to a transcript

The position of a unigene was estimated according to its alignment with a reference protein using Blastx in GenBank. Unigene pairs (two unigenes), which had a gap or did not have an evident overlap were combined and generated a supposed transcript according to the position of encoding protein. As shown in Table S1, we selected 0–2 potential *NRT* unigene pairs for each group. In total 16 unigene pairs, including 12 *NRT1* unigene pairs and 4 *NRT2* unigene pairs were selected for further experimental verification (Table 3A). As shown in Table S2, we selected 0–3 potential *PHT* unigene pairs for each group. Twelve unigene pairs, including 8 *PHT1* unigene pairs, 3 *PHT3* unigene pairs, and 1 *PHT4* unigene pairs were selected for next experimental verification (Table 3B).

**Table 3A:** **Primers and estimated PCR products for 16 pairs of selected ***NRT*** unigenes**.

**Numbers**	**Unigene pair (5′ end + 3′ end)**	**Reference location**	**Possible gap**	**Primer: forward/reverse (5′–3′)**	**Designed PCR size (bp)**	**Estimated size (bp)**
1	Unigene53952_All + Unigene31144_All	12–101+208-3′	107 aa	GACTACCAAGGAAATCCAGTGG/CAGGAAGGGCAAGACAACG	721	1042
2	Unigene67667_All + Unigene54470_All	5′–177+359–3′	182 aa	TTGGTCCTTTGCTTGGTGC/TTTTCGATGAGGGCGGC	575	1121
3	Unigene68619_All + Unigene91547_All	5′–170+164–234	none	CAAAGTGACAAATGGGAAGG/ACAATAGAGTCGTGGTGGAGAT	700	700
4	Unigene60049_All + Unigene54473_All	4–145+136–3′	none	CAGCAGTGGGAAACAACCT/ACCAGAAACCAAGCAAATCA	599	599
5	Unigene31738_All + Unigene31143_All	5′–212+203–3′	none	CGTTGTCTTGCCCTTCCTG/GCCATACTTCTCATATTCTCTGG	695	695
6	Unigene63092_All + Unigene71213_All	5′–80+78–3′	none	ATCACACCAGCAGAACACGT/CAAGAACACCCCAAAATCAA	896	896
7	Unigene49607_All + Unigene30293_All	59–245+311–3′	66 aa	TGCCTTCCTTAGTGATTCCTAT/AGATTTCCACAACTCCTGCC	768	966
8	Unigene63092_All + Unigene34485_All	5′–80+218–3′	138 aa	GTGGGGTGACCAAGAAGAGA/TAGGAGGATGCTGGCGATG	768	1182
9	Unigene85390_All + Unigene23477_All	268–392+466–541	74 aa	ACTAGGGGGATTAGGCCTTT/TGTCGTCGTTTGAACTATGGA	479	701
10	Unigene71982_All + Unigene61016_All	5′–321+482–3′	162 aa	TTGGTTTTTTGGCTTCTGC/CCTCCTCCTTTATCTTCTGTGA	560	1046
11	Unigene34465_All + Unigene61016_All	5′–430+482–3′	52 aa	CACTTCCACTTACCGCCAC/CGTCCTCCTCCTTTATCTTCTG	731	887
12	Unigene49930_All + Unigene54471_All	41–122+359–3′	237 aa	CACCTTGGGCTTCGCATA/AACGGCAGTCATCATTTCG	563	1274
13	Unigene54452_All + Unigene44258_All	5′–243+423–463	180 aa	GGGTTTTGTTTCGGGGTG/GGATTAAAGGCTTGTCAGGC	816	1356
14	Unigene34113_All + Unigene5588_All	5′–258+415–3′	157 aa	GTTCCTCATGCCCCTTGTC/TTGGTGCTTCTCTCGTTTCTAC	465	936
15	Unigene39295_All + Unigene80102_All	165–252+381–413	129 aa	TGTTATCCTTAAAACAGGGG/TTGAGTTATGACAGCACCC	437	824
16	Unigene136150_All + Unigene132777_All	5′–66+402–3′	336 aa	ATTATCAATAGCAAAGCCTC/CAACTGCTCCACTACCCT	417	1425

*Reference location indicated the location of unigene coding protein aligning with protein database of Arabidopsis thaliana. aa, amino acid; bp, base pair*.

**Table 3B:** **Primers and estimated PCR products for 12 pairs of selected ***PHT*** unigenes**.

**Numbers**	**Unigene pair (5′ end + 3′ end)**	**Reference location**	**Possible gap**	**Primer: forward/reverse (5′–3′)**	**Designed PCR size (bp)**	**Estimated size (bp)**
1	Unigene91380_All + Unigene105912_All	82–170+348–448	178 aa	TAGTTGTGGAGGAGAAATGG/AGGATGGAGAAGGTGACG	558	1092
2	Unigene5546_All + Unigene11489_All	5′–157+229–319	72 aa	CTTTTGGGGCGTCTGTA/GAACCACGTGCTTGTGG	553	769
3	Unigene5546_All + Unigene47851_All	5′–157+166–3′	9 aa	CTTTTGGGGCGTCTGTAC/TTCCCTTCGCTTTTTGTG	677	704
4	Unigene16022_All + Unigene50223_All	5′–91+417–3′	326 aa	ATTTCATCACACACCCAG/ATTTACCCATAGACTCCG	854	1832
5	Unigene16022_All + Unigene90558_All	5′–91+417–3′	326 aa	ATTTCATCACACACCCAGA/CAAGAGATTTACCCATAGACTC	860	1838
6	Unigene11489_All + Unigene63539_All	229–319+412–493	93 aa	CAGATAGACGCCGATGAGG/GCAACCAAGAACAATAAGAGAG	416	695
7	Unigene48391_All +Unigene44133_All	5′–63+404–3′	341 aa	CTTTCCACCTCAAGTCAT/GTGCAACCAAGAACAATAAGA	587	1610
8	Unigene53055_All + Unigene44133_All	5′–163+404–3′	241 aa	TCTGTATCTCCCTCCTAAC/CCAAGAACAATAAGAGAGTT	632	1355
9	Unigene125621_All + Unigene61379_All	5′–68+131–277	63 aa	CCCCATCTCCACTCCTGTT/AAGGTTGACGGTGGTGTTC	566	755
10	Unigene141416_All + Unigene129694_All	5′–233+210–281	none	CTGCTATCACCCCTCTTG/CCTTGGTTCAATTTTTATCC	745	745
11	Unigene141415_All + Unigene129694_All	8–87+210–281	123 aa	AAAGTATAATGGTAGGAAGTCC/GTTCAATTTTTATCCTCAGG	458	827
12	Unigene29837_All + Unigene46396_All	5′–230+292–3′	62 aa	GTGTTGCTTTGTGGTCCT/CCCATGTAATATTCCGGT	962	1148

*Reference location indicated the location of unigene coding protein aligning with protein database of Arabidopsis thaliana. aa, amino acid; bp, base pair*.

### Validation of unigene pairs by PCR and sequencing

**Figure 3:**
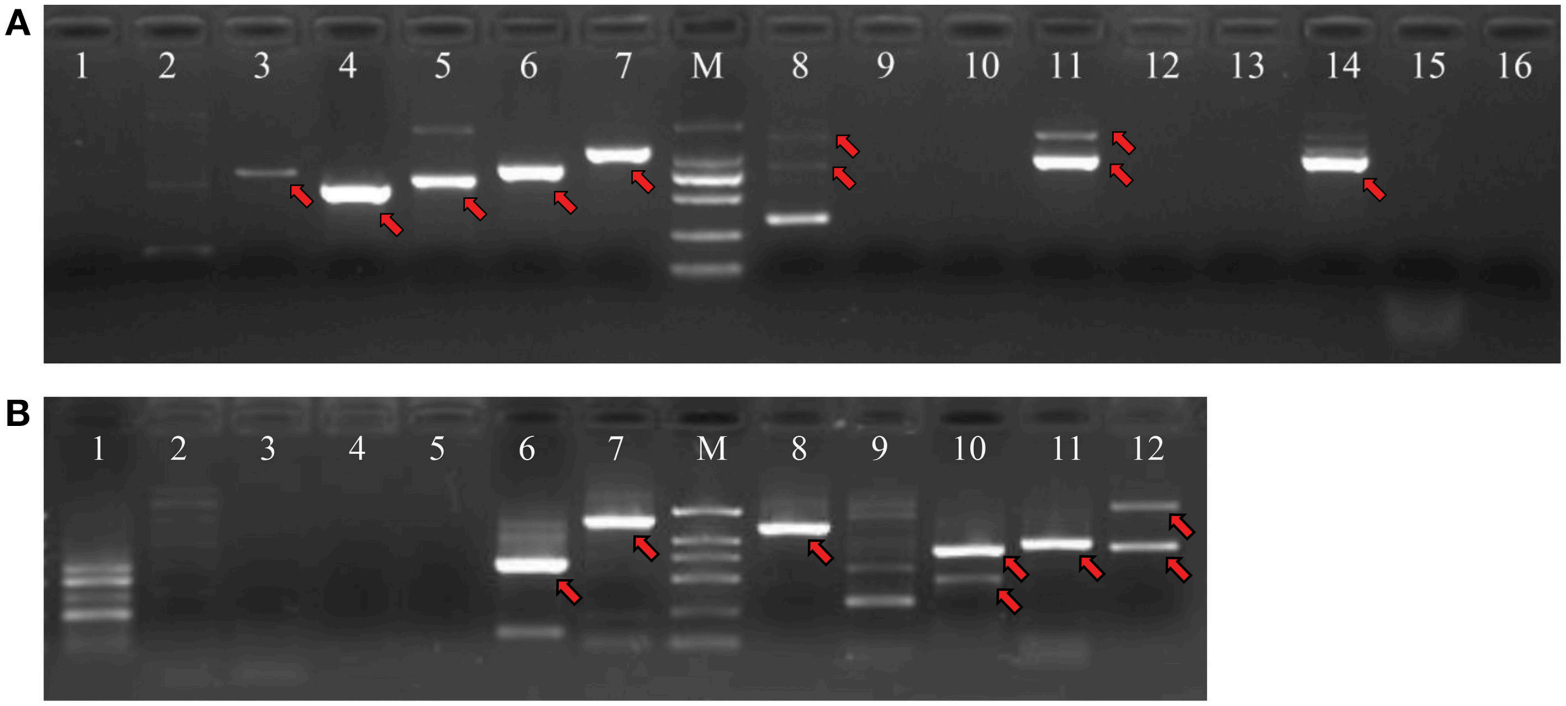
**Agarose gel electrophoresis of PCR products for unigene pairs**. PCR reactions were performed with corresponding primer pairs and a pooled cDNA template. Amplified fragments were separated by 1.2% agarose gel electrophoresis. **(A)** PCR products for *NRT* unigenes. **(B)** PCR products for *PHT* unigenes. M: DNA marker with bands of 2000 bp, 1000 bp, 750 bp, 500 bp, 250 bp, 100 bp from high to low, respectively. The serial numbers represent corresponding PCR reactions for various unigene pairs, which are coincident with the number in Tables 3A,B.

PCR products would be generated through the polymerase chain reaction using corresponding primers, if two unigenes represent a single transcript. Figure 3 shows an electrophoretic analysis of the PCR products amplified using corresponding primer pairs. One or more bands can be found in lanes 3–8, 11, 14 in Figure 3A (*NRT*) and lanes 6–8, 10–12 in Figure 3B (*PHT*). From each lane we extracted and sequenced bands (marked arrows) whose size was the size expected from the designed primers plus the size of supposed gap. We found that 10 *NRT* unigenes (5 unigene pairs: Unigene68619_All + Unigene91547_All; Unigene60049_All + Unigene54473_All; Unigene49607_All + Unigene30293_All; Unigene34465_Al + Unigene61016_All; Unigene34113_All + Unigene5588_All) and 6 *PHT* unigenes (3 unigene pairs: Unigene11489_All + Unigene63539_All; Unigene53055_All + Unigene44133_All; Unigene141416_All + Unigene129694_All) could be assembled with corresponding PCR products (Figures S2A, S2B; Data Sheets 1, 2).

### Sequence information of the assembled unigenes

According to the best alignment results against the nr protein database of *Arabidopsis thaliana*, all 5 *NRT* assembled sequences were annotated nitrate transporter (Table 4A) and all 3 *PHT* assembled sequences were annotated phosphate transporter (Table 4B). Two of *NRT* assembled sequences and one of *PHT* assembled sequences contained complete CDS as indicated by an ORF analysis and a Blastp alignment in GenBank, which could presumably be used for further research on gene function.

**Table 4A:** **Information of assembled ***NRT*** unigenes**.

**Assembly sequence**	**5′ end unigene**	**3′ end unigene**	**Assembled length (bp)**	**Encoded protein (AA)**	**Complete CDS**	**Annotation (Blastx in nr database of Arabidopsis thaliana)**
>Assembly 3 (Unigene68619_All + Unigene91547_All)	762	215	953	293	no	Nitrate transporter 1:2 (*Arabidopsis thaliana*)
>Assembly 4 (Unigene60049_All + Unigene54473_All)	429	1861	2254	591	no	Probable peptide/nitrate transporter (Arabidopsis thaliana)
>Assembly 7 (Unigene49607_All + Unigene30293_All)	561	958	1672	431	no	Nitrate transporter 1.5 (Arabidopsis thaliana)
>Assembly 11 (Unigene34465_Al + Unigene61016_All)	1655	695	2498	626	yes	Putative peptide/nitrate transporter (Arabidopsis thaliana)
>Assembly 14 (Unigene34113_All + Unigene5588_All)	821	747	2030	504	yes	Nitrate transporter2.5 (Arabidopsis thaliana)

**Table 4B:** **Information of assembled ***PHT*** unigenes**.

**Assembly sequence**	**5′ end unigene**	**3′ end unigene**	**Assembled length (bp)**	**Encoded protein (AA)**	**Complete CDS**	**Annotation (Blastx in nr database of Arabidopsis thaliana)**
>Assembly 6 (Unigene11489_All + Unigene63539_All)	274	249	789	263	no	Phosphate transporter (Arabidopsis thaliana)
>Assembly 8 (Unigene53055_All + Unigene44133_All)	619	621	1959	534	yes	Putative inorganic phosphate transporter 1-3 (Arabidopsis thaliana)
>Assembly 10 (Unigene141416_All + Unigene129694_All)	722	238	1055	345	no	Phosphate transporter 3;1 (Arabidopsis thaliana)

### Two fragments of a transcript have same expression quantities

**Figure 4:**
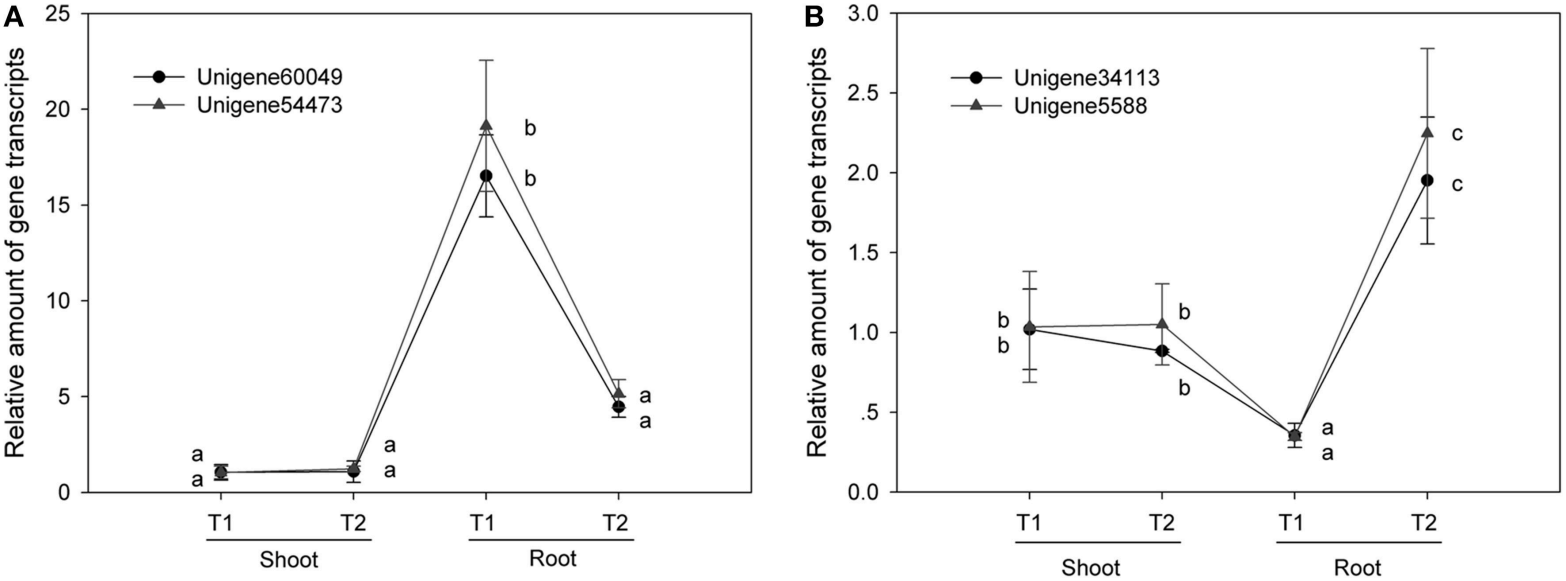
**RT-qPCR shows that unigenes for the same transcript have the same expression levels**. The same letter indicates no significant difference. T1, T2: the treatment under NaCl stress or NaCl-free conditions. **(A)** Unigenes pair 4 (NRT). **(B)** Unigenes pair 14 of (NRT).

**Figure 5:**
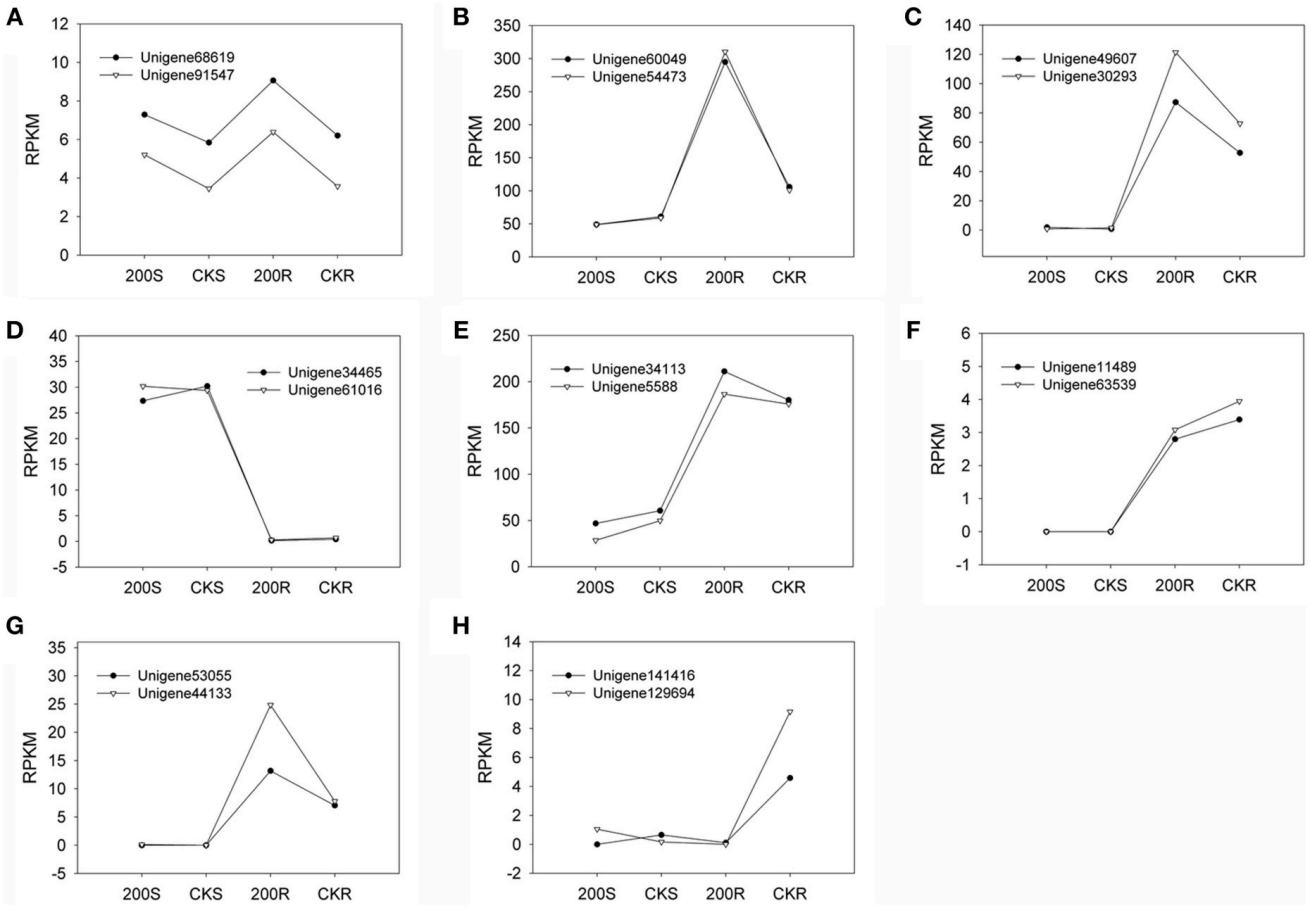
**Expression pattern for 8 pairs of unigenes belonging to a transcript**. **(A–E)**: *NRT* unigene pairs. **(F–H)**: *PHT* unigene pairs. RPKM (reads per kilobase of exon model per million mapped reads): a method for indicating the expression level of a unigene. Four transcriptome libraries (200S, CKS, 200R, CKR) provided samples for RNA-seq.

We quantified the expression levels of two pairs of unigenes (Unigene60049_All and Unigene54473_All; Unigene34113_All and Unigene5588_All) under various NaCl stress conditions via real-time qPCR technology (Table 5). There was no significant difference between the two unigenes for a single transcript in terms of expression level under the various conditions investigated (Figure 4). Furthermore, we compared the RPKM value between the two unigenes in each pair (comprising a transcript) for four RNA-seq samples (Figure 5) and found that the values were basically consistent. Although, the RPKM values of the unigene pairs (Unigene68619_All + Unigene91547_All) were not equal under the different conditions because of the bias of the RNA-seq method (Mortazavi et al., 2008), the trends of change were identical among the various samples. RPKM is a common approach for calculating the amount of the expression of many unigenes via RNA-seq, and this study indicated that the unigenes representing a single transcript had the same expression patterns based on clustering analysis of RPKM. It is an effective method to distinguish the unigenes representing a single transcript over a large range of expression levels (i.e., an RPKM range from zero to tens of thousands or higher). Moreover, the greater number of RNA-seq samples one obtains, the more expression patterns are available to single out unigenes, making this method more effective to find the unigenes for a single transcript. Unigenes that have the same annotation and same expression pattern in multiple samples are very likely to arise from the same transcript.

**Table 5:** **Gene specific primers for determining expression of unigenes using RT-qPCR**.

**Name**	**Sequence length (bp)**	**Annotation**	**Forward primer (5′–3′)**	**Reverse primer (5′–3′)**
Unigene60049_All	429	NRT1/PTR	TCCCTCCTTGGTGGTTACCT	GTTGTGGTAGGTGTGCTTGC
Unigene54473_All	1861	NRT1/PTR	AAGTAAGCCCGGACGTGAAG	ATGCATGCCTTGTCCAAACAC
Unigene34113_All	821	NRT2	CAGTTCCTCATGCCCCTTGT	TTGGCCGAAAAGCATGACTG
Unigene5588_All	747	NRT2	AGTGGGGAGGTGCATTTTGT	TCGTTTCTACTGCCTTCAGCA

## Discussion

RNA-seq has been widely applied to obtain transcriptome data because of its great advantages (Van Verk et al., 2013). Identifying a full set of transcripts assembled from huge amounts of raw reads is an essential requirement for the deep study of a species. However, at present, no assembly application can generate a complete transcriptome containing full-length transcripts (Steijger et al., 2013). The number of unigenes is far higher than the estimated number of transcripts and most unigenes are not full length (Schliesky et al., 2012), which indicates that two or more unigenes belong to the same transcript but exist separately in the transcriptome library. Therefore, some of these unigenes could be reassembled.

To find the unigenes that belong to a particular transcript is not easy in a transcriptome library that contains more than a 100,000 unigenes. Unigenes that have different annotations are unlikely to represent the same transcript, therefore limiting the analysis to a gene family or subfamily (i.e., one in which the unigenes that have same Nr or Swiss-Prot annotation) of interest will greatly reduce the difficulty of finding the unigenes of a single transcript. The number of unigenes for a gene family, which usually ranges from several to hundreds, depends on the size of the member genes and the quality of the assembly. In a *S. europaea* transcriptome library, we found 118 *NRT* unigenes, which could be further divided into 75 *NRT1* unigenes, 37 *NRT2* unigenes and 6 *NRT3* unigenes. It was more practical to purposefully analyze a small number of unigenes by excluding many irrelevant unigenes, and validate the results of the analysis experimentally.

It has been shown that different unigenes for the same transcript have same expression level under identical conditions per RT-qPCR (Figure 4). Attempting to find the unigenes for a transcript based on same expression pattern under various conditions is a novel approach. RT-qPCR is a powerful tool for detecting gene expression because of its accuracy and sensitivity, but the number of detecting genes is limited (Xiao et al., 2015b), however, we can generate expression profiles for thousands of genes via RNA-seq, using a method such as RPKM, calculated based on the rate of randomly-picked reads and sequence lengths (Grabherr et al., 2011). Although, the unigenes, belonging to a given transcript might not show identical RPKM values because of a difference in the bias of the RNA-seq outcome (Wang et al., 2009), the values are approximate and the changes in the expression trends proved to be consistent among various RNA-seq samples (Figure 5). The unigenes were clustered based on their expression patterns, therefore, the more RNA-seq libraries analyzed, the more likely that the unigenes of a given transcript could be distinguished from unrelated unigenes. Clustering unigenes that have same expression pattern greatly reduces the combinations of unigenes that must be reassembled. Adding more unigenes to a group will yield more combinations so the loss of possible unigene pairs can be avoided, but the workload is greatly increased. In this study, 2–5 unigenes with similar expression patterns were grouped according the clustering results and we found that 8 pairs of unigenes could be reassembled from 118 *NRT* unigenes and 47 *PHT* unigenes.

To further decrease invalid experimental validation, impossible combinations of two unigenes in a group were excluded if their positions of the proteins coded conflicted with each other with respect to the protein sequence of *Arabidopsis thaliana*. We assumed that if the protein coded by two unigenes overlapped but the overlapping sequences were not identical, the unigenes must belong to a different transcript and were unlikely to be assembled. Although, the RNA-seq reads were assembled using the Trinity application, there were still some unigenes for particular transcripts that could not be assembled because of gaps. This study introduced a method to find these unigenes and fill the gaps.

Some unigenes might belong to a different transcript even though they have the same expression pattern. Therefore, putative unigene pairs should be validated by PCR technology (Saiki et al., 1988). The primer pairs contain forward and reverse primer sequences which were designed by mapping two different unigenes. If the two unigenes belong to the same transcript, the PCR product will be amplified exponentially, otherwise, no bands (except nonspecific amplification bands) can be found in agarose gels after electrophoresis. We validated 5 pairs of *NRT* unigenes from 16 pairs of putative unigenes and 3 pairs of *PHT* unigenes from 12 pairs of putative unigenes to comprise transcripts. The success rate of assembly can reach 31% and 25% respectively. Thus, we have discovered a feasible method for the further assembly of target unigenes.

## Conclusion

Unigenes for a particular transcript that remain unassembled in transcriptome libraries after performing RNA-seq assembly using present assemblers still exist. Unigenes having a similar annotation and the same expression pattern in multiple samples are very likely to represent the same transcript. We introduced a novel approach to distinguish these putative unigenes and to further assemble a few unigenes of a gene family after experimental verification, which is a quick and useful way to extend incomplete cDNA sequences or obtain complete coding regions. This method was validated by assembling unigenes from *NRT* and *PHT* gene families and successfully produced 8 assembled sequences. Moreover, because it involves common bioinformatics tools available online and routine molecular biological techniques, biologists can easily grasp the method. We suggest that this method be given priority for use to assemble unigenes of interest when extending incomplete CDS before using rapid-amplification of cDNA ends (RACE) technology, because it assembles more unigenes more cheaply and is easy to operate without a complicated bioinformatics analysis.

### Conflict of interest statement

The authors declare that the research was conducted in the absence of any commercial or financial relationships that could be construed as a potential conflict of interest.

## Supplementary material

Figure S1A **Hierarchical clustering of expression pattern for the three types of ***NRT*** unigenes**. The unigenes on the closer branch had more similar expression pattern, which were grouped and marked a red box. The number represents the base pairs number of corresponding unigene.

Figure S1B **Hierarchical clustering of expression pattern for the four types of ***PHT*** unigenes**. The unigenes on the closer branch had more similar expression pattern, which were grouped and marked a red box.

Figure S2A **Alignment between a PCR product and corresponding ***NRT*** unigene pairs using Blastn in NCBI**. A PCR product served as the Query sequence.

Figure S2B **Alignment between a PCR product and corresponding ***PHT*** unigene pairs using Blastn in NCBI**. A PCR product served as the Query sequence.

Table S1 **Information regarding 27 groups of ***NRT*** unigenes**. The unigenes were grouped according to the clustering of the expression patterns. The reference location indicated the location of unigene coding protein aligned with the protein database of *Arabidopsis thaliana*.

Table S2 **Information regarding 11 groups of ***PHT*** unigenes**. The unigenes were grouped according to the clustering of the expression patterns. The reference location indicated the location of unigene coding protein aligned with the protein database of *Arabidopsis thaliana*.

Data Sheet 1 *****NRT*** sequences of ***Salicornia europaea*****. The doc file contains the sequences of 16 unigenes pairs, 10 sequences of PCR products, and 5 assembled sequences.

Data Sheet 2 *****PHT*** sequences of ***Salicornia europaea*****. The doc file contains the sequences of 12 unigenes pairs, 8 sequences of PCR products, and 3 assembled sequences.
