# Supplementary figures and images for: A method for the further assembly of targeted unigenes in a transcriptome after assembly by Trinity
Source: Front Plant Sci. 2015 Oct 14;6:843. doi: 10.3389/fpls.2015.00843 (PMC4604318; doi:10.3389/fpls.2015.00843)

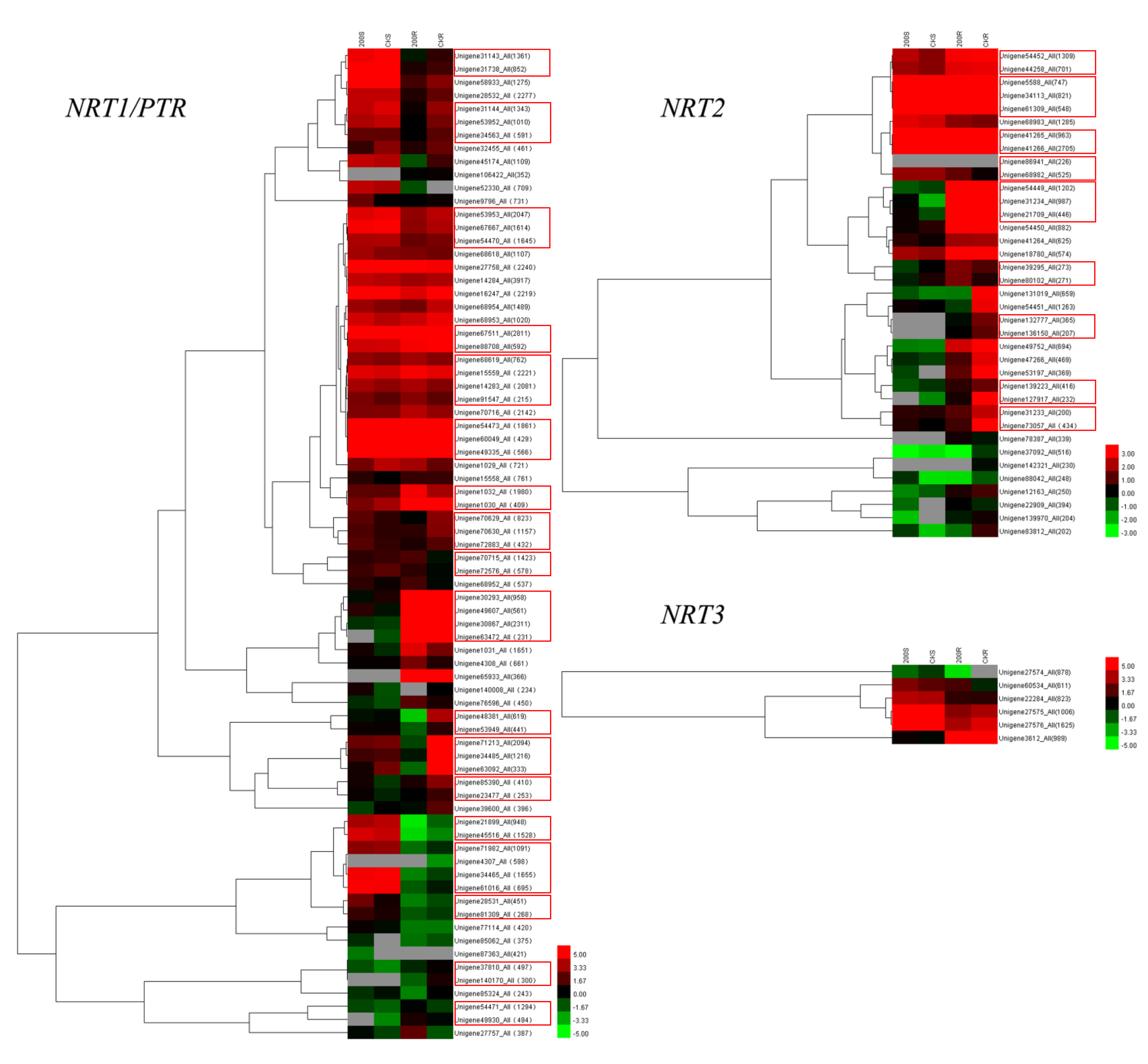

Supplement: Figure S1A — Hierarchical clustering of expression pattern for the three types of NRT unigenes. The unigenes on the closer branch had more similar expression pattern, which were grouped and marked a red box. The number represents the base pairs number of corresponding unigene. [file FigureS1A.TIF]

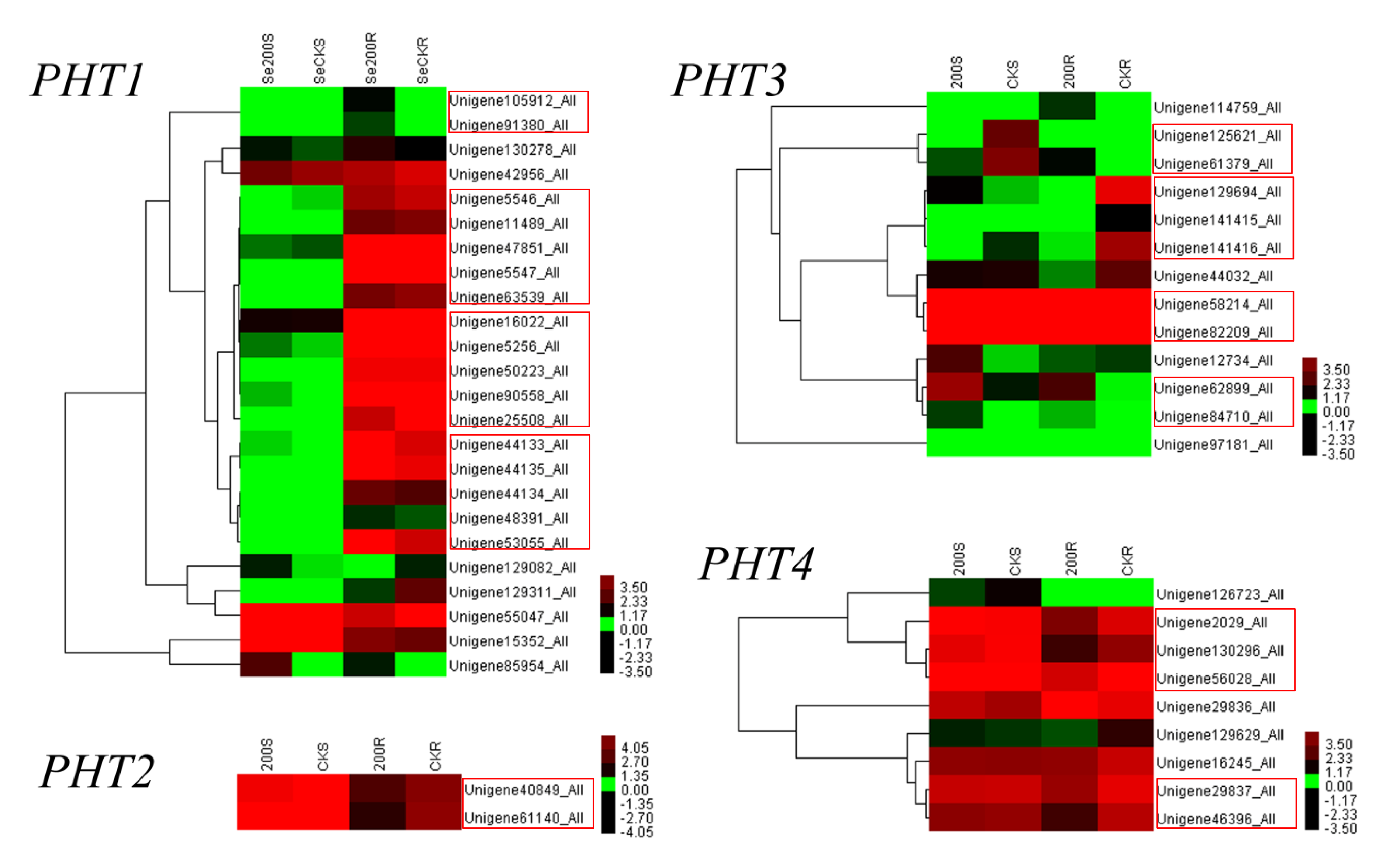

Supplement: Figure S1B — Hierarchical clustering of expression pattern for the four types of PHT unigenes. The unigenes on the closer branch had more similar expression pattern, which were grouped and marked a red box. [file FigureS1B.TIF]

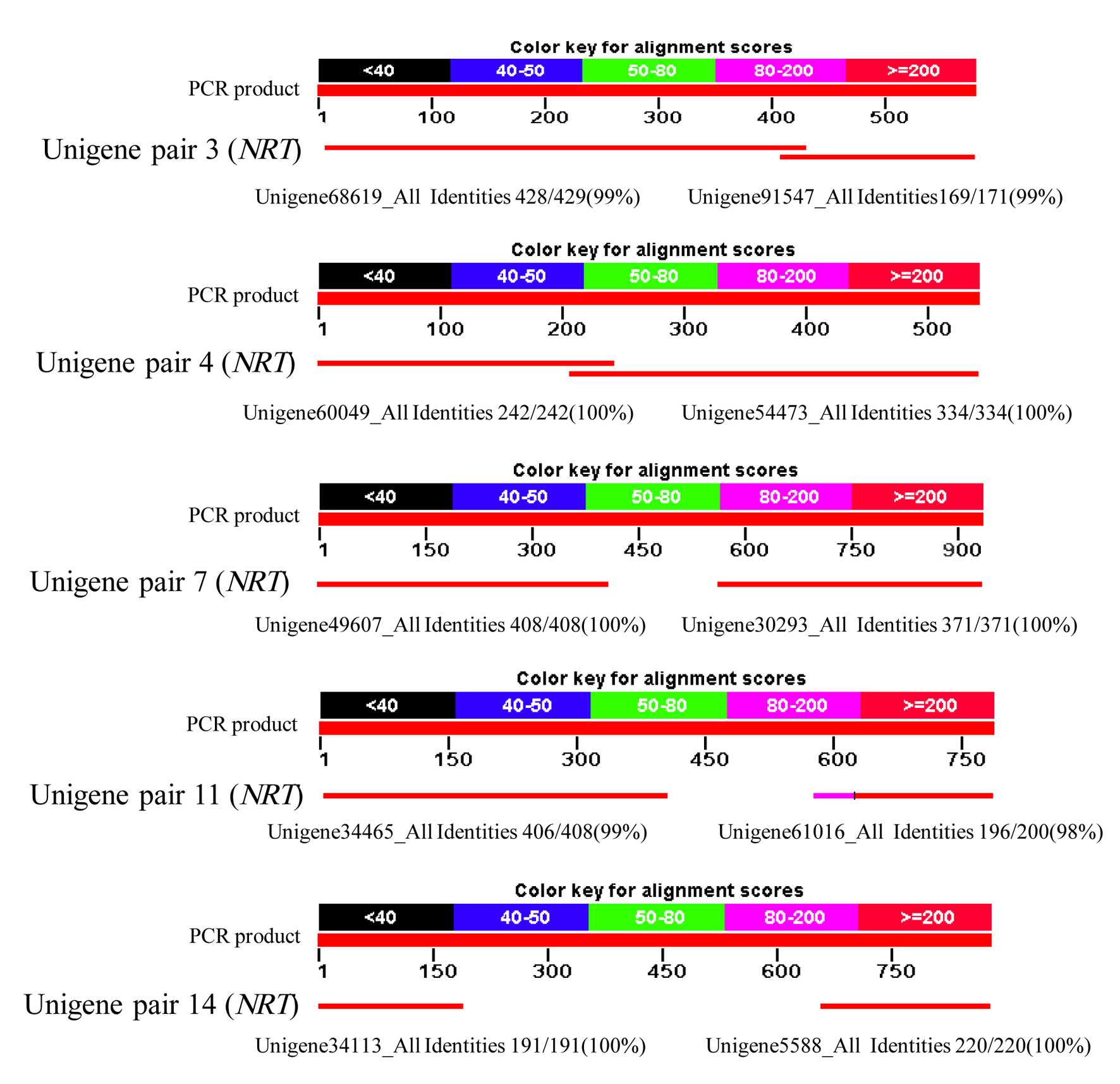

Supplement: Figure S2A — Alignment between a PCR product and corresponding NRT unigene pairs using Blastn in NCBI. A PCR product served as the Query sequence. [file FigureS2A.TIF]

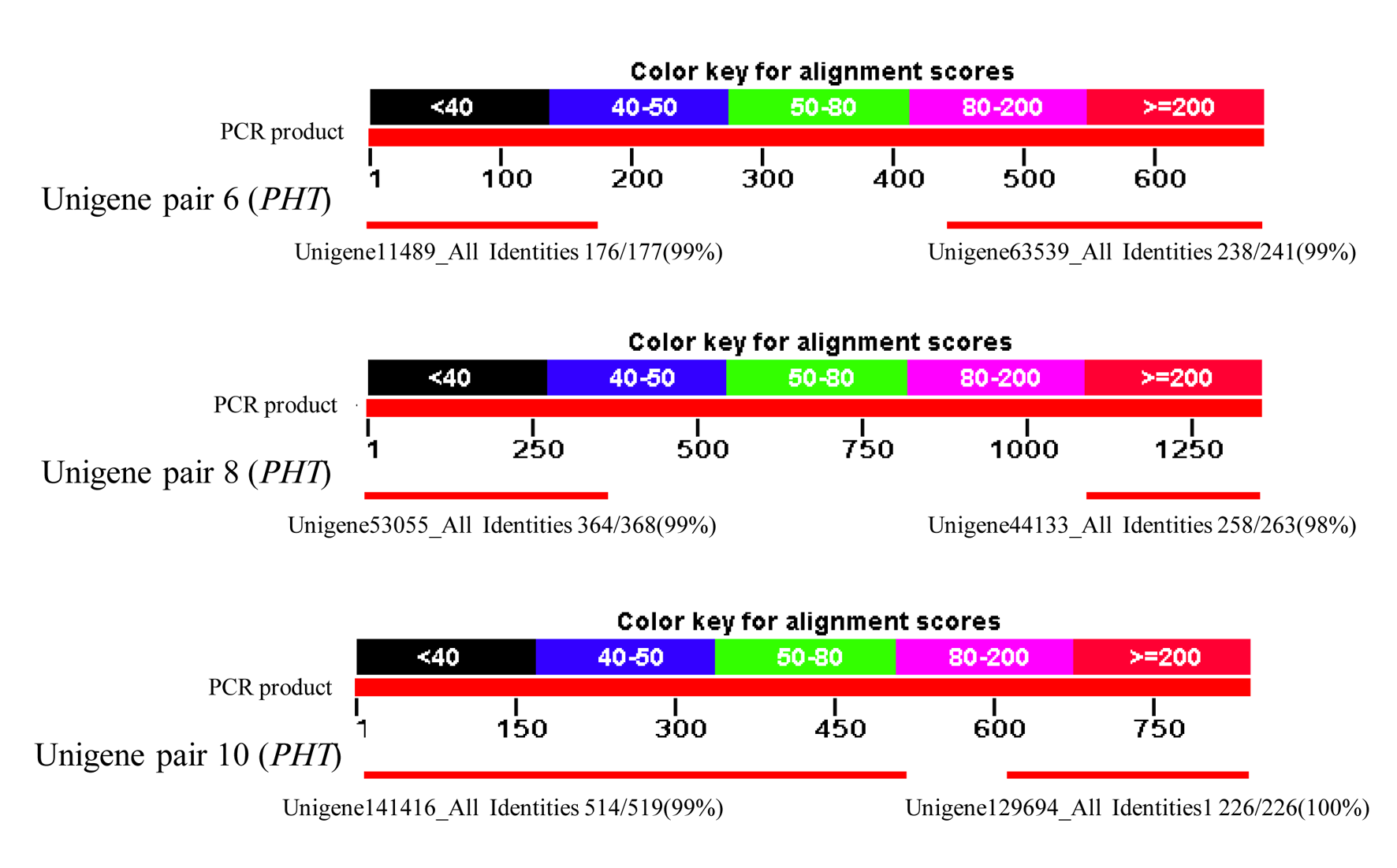

Supplement: Figure S2B — Alignment between a PCR product and corresponding PHT unigene pairs using Blastn in NCBI. A PCR product served as the Query sequence. [file FigureS2B.TIF]
