# Supplementary material for: A method for the further assembly of targeted unigenes in a transcriptome after assembly by Trinity
Source: Front Plant Sci. 2015 Oct 14;6:843. doi: 10.3389/fpls.2015.00843 (PMC4604318; doi:10.3389/fpls.2015.00843)
Supplement: Data Sheet 1 — NRT sequences of Salicornia europaea. The doc file contains the sequences of 16 unigenes pairs, 10 sequences of PCR products, and 5 assembled sequences. [file DataSheet1.DOC]

**Sequences of 16 unigene pairs**

>Unigene53952_All - nr

AATGTCTTTCATCAAGATTGATGGAGCATCACTAATTTCCATTCCACTCAAATTTATGTA

ATTTGACTGAGTGACTCACTGTAATCTTACAGATGTGCACCCTGTCAAAAATGTCAGTAT

CATCAGTTTAGCTGTTTAGGCTTCAAAACTTGAGTCTATAAAAACCTCATTATTTCATTA

ACATACTATCATTCCTAACCAACTTAAAATTCCCTCTTTACCATTGAAGCTTTGTACCCA

ATTTAGGATCTACTTTCAACTTGTTTCCCCAAACCTCCATTCAATTATTTCACTTGCCCA

TTCACTTGTTTGCTGTCATTACTGCAACTGTATCCAGGACAAAGCTCCACCCCTGTTTTC

TTGCTTCTTGTTATTGCATTATATACCTCTCTTTGTACCTCCCTCTTCTCTCTTTCTATC

ACACATTTTCATTCTCAATTTGTACATTGATTCCCTCATTTCTCACTAGCTTCTTTTCAG

TGCTTCCTTACATACTGATACCATCATGGTTTTGGCTGGAGAGTTTGATAGAAATGGTGA

TGAAAGTGAAGCTGTTACTGACTACCAAGGAAATCCAGTGGACAAGTCTTGTACTGGTGG

ATGGCTTGCTGCAGGCCTCATTTTAGGAACTGAGTTTGCTGAGAGAGTATGTGTGATGGG

GATATCCATGAATTTGGTCACTTACTTGGTTGGATATCTGCATCTTACTTCATCAAAATC

TGCTATAATAGTGACCAATTTTATGGGAACCCTTAATCTTCTTGGCTTGTTTGGTGGATT

TGTTGCTGATGCTAAGCTCGGCCGGTACTTGACTGTTGCTATCTTTGCATCTATAGCAGC

TGTGGTGAGTTTCAGATTCTCAACTTTGCATCATTTTTTTTGGCTAATTTGGGTAACTAA

GGCAAAATTATGGATCTTTTTGCAAGAAACAAAAAGAAGTATGTGTGTAAAAATAAACAA

AGAAAAGAAGCAAGAGAAGAAAAGAAGCAAATCTTTTTGCAGGACATGGG

>Unigene31144_All - nr

GAAGAAGAGGAATAAACAGTTATTACAATTGGTATTACACAACATTCACAATTGTAGTAA

TGATAACATTAACAGTTGTGGTTTATATACAAGACTCAGTGAGTTGGGTTTGGGGTTTTG

GGATCCCAACAAGTCTTATGTTGTGCTCAATTTTGCTCTTCTTCTTTGGAATGAAGTTTT

TTGTGTACATCAAGCCTCAAGGAAGCATCTTCTCTAGCATTTTTCGAGTATTTGTTGCCG

CGTATAGGAAACGTTGTCTTGCCCTTCCTGATCATCATCGTGGCGTTGATAAAGGTGTTT

TCTATGATCCTCCTCTTTCTGGAGTTCTTCAATCAGTGCTCCCTCTTACCAATCAATATA

GTGGTTTAAACAAAGGAGCTATAATACAAGAAGGAGAGCTGAGGTCAGATGGCAGAGCAA

CAGCAGGAAGCAAATGGAGGTTATGCAGCATCCAAGAAGTAGAACAAGTAAAATGTGTAC

TAAAAGTGCTTCCAATTTGGGCGTCTGGTATAATCTGTTTCACATCAACGGCTCAGCAAT

CAACCTTCGCAGTGTCCCAAGCCATGATCATGAACCGAAACATAGGCCCGAAATTTCAAA

TCCCACCTGGGTCAATAACCATCATATCTATGCTAACCATAGGAGTGTGGGTCCCAATCT

ACGACCGGTTCGTGGTCCCGGCCCTTAGGAAGATCACGAAACACGAGGACGGCCTCTCCC

TCCTCCAAAGGATGGGGATCGGCCTCGTATTCTCGACACTTTCGATGGTGGTGGCCGGGT

TAGCCGAGCACAATAGAAGGGCTAATGCTAACTTGCACCACAATAACAGAGTATCAGTGA

TGTGGTTAGCCCCTCAACTAGTTATGCTAGGGCTTGCTGAAGGGTTCAATTTTATAGGTC

AAATTGAGTTTTATAATAAGCAATTTCCTGAGAATATGAGAAGTATGGCTACTTCTATGT

TCTTTTGTACAATTGCTGGGGCTAGCTACTTGAGTTCTGCTGTGGTTACTTTGGTTCATA

AGTTTACTGGTACCCCTGATTGGTTAACCAATGATCTTAACCAGGGTAAGGTGGACTATT

TTTACTACTTGCTTTCTGTTATGGGGCTCTTGAATTTTATTTACTTTTTTATTGTTGCTA

GGAATTACAAATATAAAGGCATTAATTATGATACTAATCATCATGATATAGAACTTGATC

CAGTTATTAAGCATTAATAACTAATTAATTAATTAAAAAGGAGGGATATTAGCTAGAGGG

AGGGACAGTTATTTAATTCTCTAGTTTAAGGTATATATAATAATATATAGTTTTATATAT

ATATGTAATGTAATAAGCAAAGG

>Unigene67667_All + nr

ACGAAATCAAAATATAGTTCATTACTACAAATAATAATTCATTACTAAATAATATACAAC

AAAATTTTGTCAAAGATTTTTGTTCCCGTGCTTTGGACCAACACACACTATTACAGCAGC

AAAGTCTTTTTTTTCTGTTTCAACTTCGCTGGAAAACGGAGGCAAAATAAAAAAGAGTTC

CAATAAACCAAAGAACCCTAAAAACAAGATAACAAAATTAAATTAAAGAAAAGGCCTTCC

TCAATTTTTTCTTTTTTGAGAAAGCCCTAAAAAAACTAACATAAAAAAAACCCAGAAAAT

TTTTATAAAAAAAATCGAGAAAATTCAGTGGATGGATCTGCAAAGAGGGCAAGTAGAGTG

ATGATTATCGAACCATTTATCCAAACAATTAGTATGAAAAAAATGGTTACAGGACAACTC

ACTCACCTCTTCTTCATCCTCAAATTTACATAAACAAACACAACATTCATCTGTTCTTCT

AATTTCACCTTCAAATTCAACCTTAACCTTTGAATCTTCATCTTCTTCATTTCTTCTTTT

TCTGCTGCTGCTGCTGCTGTTGATTTTACTGGGCTTATCACACAAACACTTGAACTGTGT

AATTGATACATCTCTAATCCTTGAAATTCTGTAACTACTGTTTGGTTGGTATAATTCATT

ATTTGTTGATTGATTTTCTTCAAAATTTCTGGTTACCCAGCTTAGTGCTCCCATTAATTG

AAGAAATGACCTCAGTAAATTCTTCAGTATTGCTACTGACATTACTGTGTTCATCACTAG

TATTGGTAATACCCCTTCTGCTGGGCTTGGAAAATTTGATAACCCCATTTGATATTTTTT

TTAACAGATGTCAGATTTTTTAATGGAATGAAAGATTTTAGAGGGAGAAACTGAGGATTT

TGTTTTGGTTTTAGAGAGAGAGAGAGAGAGCCAGAGAGATATATAACAATAATGGAGAGA

GAAACTAACAAAGAGAAAATACATTCTCCTTCTTCTTCTTCTTCTACTACTACATGGTTA

TCATGGGGTTGTAATTTGGCACCATTTTGCTTAACTTCAATAAAGGATTCTTCCAATAAT

GTTAGCATGGGGAGCTCTAACCAGAACAAGCCTGGTGGATGGAAGTCTATGCCTTTTATT

TTAGGGAATGAGACATTTGAGAGGCTAGCAACATTTGGGGTAATAGCCAACTTCATGGTG

TATTTAAGAAGGGAGTATCATATGGACCAGGTGGCTGCCGTTAACGTCATCAACATTTTT

TCCGGGGTCTCTAATTTTGGTCCTTTGCTTGGTGCTTTTATTTCTGATGCTTATATTGGT

CGCTTTTGGGCTATCGCTATTGGTTCTTTGGCTTCTTTTTTGGGAATGTTGGCACTAACA

TTAACAGCTTCAATCCCAAGCCTAACACCACCTTGGTGTCCATACAATGACCAAAAGAGC

CAAACAATAGGTCCACAGCAGATGGGCTCATGCACAAAGCCCAATGGGCCACAAATGGGC

TTCTTAATACTAGGCCTTGGGCTGCTAAGCATAGGGACAGCAGGAATAAGGCCATGTAGC

ATACCATTTGGGATGGACCAATTTGATCACACCACAGAAGAAGGAAGAAGAGGA

>Unigene54470_All + nr

CCAACAAGGCAGTCAAATGAACACCCAACTCACTTCCTCCATAAACATCCCCCCAGCCTC

ACTCCAATCCATTCCTTACCTCCTTCTCCTCTTCCTCGTCCCACTTTACGACAAACTCTT

CGTCCCTTTCATCCGCCGCTTCACGGGCCACCCCTCCGGCCTCTCCCCTCTCCAACGTAT

CGGGTCCGGCCTCTTCCTCGCCACCTTCTCAATGATCTCCGCCGCCCTCATCGAAAACCA

CCGTAGAAACCACCAACCACCTCTCTCTATCTTCTACATCACCCCACAATTCCTCATATT

TGGACTATCCGAAATGATGACTGCCGTTGGACTTATTGAATTTTTTTACAAACAGTCACT

TAAAGGAGGGTCATTACAGTCTTTTCTTACGGCGATTACTTACTGCTCTTACTCTTTTGG

GTTTTACCTTAGCTCTGTGTTGGTTACCCTTATTAATAAACTCACTTCTTCTTCTAAGAC

TAATTATCAAGGGTGGTTAAGTGATAATGACCTTAATAATGATAGGTTAGACTTGTTTTA

TTGGTTGTTAGCTGCCCTTAGCTTCTTAAACTTCCTTAATTACCTATTTTGGTCTCGTTG

GTACTCTTTTAATCCTTCTCTTTTGCTCTCTTCTCAACCACATGTTTCTCCTCATGATGA

CCTCACCCCTCCTCCTACCAATAATACACATTCATTAATTTAATTAGATTATAATTAAAT

TAAGGAAAATTTGTTAGAAACCACCTTATGAATTAAATTTTTCGTCAAAAACCACCTTTT

AAATTATTATTTTGTTTGTAAGAAGCCACCTTTTTTGGATTTTTTTTTTGGTGAGGAACC

ACCTTTGGTTGAATTTCGGGTTTCGATTTTGGGTTATTTTTGGCGTGGTGGTTCAGGGGA

GGAAAAGGAACGGAGACTGTTTGGGGGGTACGAAGGGGGTTTGTCGGAATGGCAGGGGTT

ATCGAAATTCGGACGAAGGTGGTTTTTAAAAAAAAAAGATGGTTTTTGAAGAAAAAAAAT

TTATAAGATGATTTTTAACAAATTTTTCATTTTTTTAATCAAAAAATAATAATAATTGTA

TAATAATATTGGTGATATTATATATATAATTACCATGAACTGAGTGGGTAGTTATGAATG

CAATCATTCCAAGGATTAGAGGGATGATTGTGTGATCTGCTATCTTTTAAAGCCTTCCAA

ACCAAACTCCCAATCTTAAAACCAGTCCCAAATGCAATCATCCAAACTCTATCCCCTTTC

TTAATCCGCTGTTTTGCATCAAAATAAGCTAATTCATAAAACACTAAGCTACTTGACGTG

TTCCCAAACCGATGAAGTGTCATCTTAGCTGGCTCCACAATTAAGTCATCAAGCTTCAAC

AATTTTTCCACTTGCCCAATCACTGCCTTACCCCCCGTGTGAATGCATATGTGCTCGAAA

GCCGTTGTGAAATCCGGCACCATCGGCTTTGACTCGCCCTGAGTTAACCCGTTCAGCACC

ACCGATCTAACGTACTGAACGAGCTGACTCAGTGGAAGGACTCGTGGGGCCAGGACTTTT

ATATGCGCCCTTAAATTATGACCCGCTACACGGATCAAGTCTTTAGTGAGCGACACGCCC

GTGTTGCCCTTCCCGTCTTCTTCCTG

>Unigene68619_All + nr

GTTATCGAAAGATACAATGATTCCTAAATCCTACATAAGCATTTTTAGAGTCCTTTAAAT

AGTGTCCCATCAGCCATCGTATTCCCCAAATTAACGTGTTTTTACTCGAACAAACAAACA

AACAAACAAACTTCTCTCAAAAAATCCATTACTATAATCTCCCACCTTCTTCAATTCTTC

TCTACCAATCACCTTCATCTTATTTCTTACTACCTCCATTTCCAATCAATATTATGGAAG

TAGAAAATCAAAGTGACAAATGGGAAGGCTACGTCGATTGGAAAGGCCGCCCTGCTCTCC

GCCACCGCCACGGTGGCTTTCTTGCTGCTTCCTTTGTTCTTGGGGTGGAAGTGATGGAAA

ATATGGCGTTTTTAGCAAATGCAAGCAATTTGGTGATGTATGTATCAAAAGACATGCATT

TTACACCGTCAAAATCATCAAACACCGTTACCAATTTCATGGGCACTGCTTTTCTCCTTG

CTTTGCTCGGTGGTTTCCTCTCTGATGCCTTTTTCACCACCTTTCAAGTCTACCTTACTA

GTGCTGCCCTTGAGTTTTTGGGACTAGTATTGTTGACGATTCAAGCATACAAACCGTCCC

TACAACCACCAAAATGCAACCCAATGGACACCACAACAGGAGGAGCGTGCAGGGGACTTG

ATACTTCTGAAGCAGCAATGTTGTACATAGGCCTATACCTAGTGGCAGCCGGTGTTGGAG

GGATAAAAGGGTCATTACCAACTCATGGCGCGGAACAATTTG

>Unigene91547_All + nr

CAACTCATGGCGCGGAACAATTTGATGAGAGCACCCAACAAGGGAGGATAAAAAGATCTA

CCTTCTTCAATTACTTTGTGTTCTGCCTATCGGCTGGTGGTCTCATTGCAACCACGTTTG

TGGTCTGGGTCGAAGACAACAAGGGTTGGAAATGGGGCTTCCTCATCTCCACCACGACTC

TATTGTTATCTGTTCCTGTCTTCCTTTGTGGCTCC

>Unigene60049_All + nr

TCCACGTATGAACTCTTTAAGGAATAATAATAATAATAATAATAATGGAGAATCTGGAAT

TACTGATCAAACTACTGTTGATTGGAGAGGCAGACCTTCCAATCCTACTAAGCATGGTGG

CATGAGAGCTGCTCTCTTTGTTCTTGGGTTACAAGGATTTGAGATAATGGGAATAGCAGC

AGTGGGAAACAACCTGATAACGTACGTGATAAATGAAATGCATTATCCGTTGTCAAAGGC

AGCGAATATCGTCACCAATTTTATTGGCACCATTTTCCTCCTCTCCCTCCTTGGTGGTTA

CCTCTCTGATTCTTTTCTTGGCAGCTTTTGGACCATGCTCATCTTTGCCTTCGTCGAACT

TTCTGGATTCATCCTACTATCAGTGCAAGCACACCTACCACAACTAAAACCACCAAAATG

CAACATGCT

>Unigene54473_All + nr

CCTACCACAACTAAAACCACCAAAATGCAACATGCTAGAAGCAGATAAAGTATGTGAGGA

AGCAAAAGGGATAAAAGCAGTCATATTCTTTGCAGCACTTTACTTAGTAGCCTTAGGAAG

TGGTTGTGTAAAACCTAACATGATAGCTCATGGTGGTGATCAATTCAACTCTAACCAATC

TAAACAACTCTCCACCTACTTCAACGCCGCCTACTTCGCCTTCTCCGTTGGTGAGCTCAT

CGCCCTCACCGTCCTCGTTTGGGTCCAAACCCATTCGGGTATGGATATCGGGTTTGGTAT

CTCTGCTATTGTCATGGCTATGGGCTTGATTTGCTTGGTTTCTGGTACTCTTTTTTACAA

GAACAAGAGGCCTCGTGGTAGCATTTTCACTCCTATTGCTCAGGTATTTGTGGCCGCATT

TTTAAACAGAAAGAAAGTAAGCCCGGACGTGAAGCTCCTTCATGGAAGTTACAATGCATC

ACACAATAATCTCATACACACTGAGAGATTTAGGTGTTTGGACAAGGCATGCATAAAAAC

AGAAGGAGAAGAACAAGGACCATGGAGAGTATGTTGCACAGTAACACAAGTAGAACAAGT

AAAACTCCTAATATCAATCCTCCCAATATTTGGTTGCACAATAGTATTCAACACTATCTT

AGCCCAACTCCAAACATTCTCAGTCCAACAAGGCAGTCAAATGAACACACAACTCACTTC

TTCATTAAGCATACCCCCAGCCTCACTCCAATCCATCCCTTACCTCCTCCTCCTCTTCCT

CGTACCTCTCTACGACAAACTCTTCGTCCCTTTCATCCGCCGCTTCACGGGCCACCCCTC

GGGCCTCTCCCCTCTCCAACGAATCGGGTCGGGCCTCTTCCTCGCCACCTTCTCCATGAT

CTCCGCCGCCCTCATTGAAAACCACCGTAGAAACCACCACCACCAACCGCTCTCTATCTT

CTACATCACCCCACAATTCCTCATATTTGGACTATCCGAAATGATGACTGCCGTTGGACT

TATTGAATTTTTTTACAAACAGTCACTTAAAGGAGGGTCATTACAGTCTTTTCTTACGGC

GATTACTTACTGCTCTTACTCTTTTGGGTTTTACCTTAGCTCTGTGTTGGTTACCCTTAT

TAATAAACTCACTTCTTCTTCTAAGACTAATTATCAAGGGTGGTTAAGTGATAATGACCT

TAATAATGATCGGTTAGACTTGTTTTATTGGTTATTAGCTGGGCTTAGCTTCTTAAACTT

CCTTAATTACCTATTTTGGTCTCGTTGGTACTCTTTTAATCCTTCTCTTTTGCTCTCTTC

TCAACCACATGTTTCTCCTCATGATGACCTCACCCCTCCTCCTACCAATAATACACATTC

ATTAATTTAATTAGATTATAATTAAATTAAGGAAAATTTGTTAGAAACCACCTTATGAAT

TAAATTTTTCGTCAAAAACCACCTTTTAAATTATTATTTTGTTTGTAAGAAGCCACCTTT

TTTGGATTTTTTTTTTGGTGAGGAACCACCTTTGGTTGAATTTCGGGTTTTGATTTTGGG

TTATTTTCGGCGTGGTGGTTTAGGGGAGGAAAAGCGATGGAGACGCTTGGGGAGGTACGA

AGGGAGTTGCCGAAATCATGGAGGGAGGTTTGATTTAAACACCGAAACTAAGCTAAAGGT

GATTCTTTACAAAAAAAAAAAAATTAAAAAAAGGTGGCTTCTTACAAAAAAAAAAAAAAT

TTAAGGTGGTTTTTGACACAAAACAAAAATGTTTTATAAGGTAGTTTTTAACAAATTTTC

CTTAAATTAATGTATAATTTCATGATATTTCTTTCTTATTATGTACTAATTAATTAATTA

G

>Unigene31738_All + nr

TAAATAAATCCATTATAACATAAGTTACTTACAAAACTTTGCCTATATAAGGGAACCTCC

CATGCTATCTTTATATATTGCAAGTCCAAGCCAATTCAAGCAATTTATTAAGCATATTAA

TTACTTTGTTTGTCACATAAAATATTAGCACATCCATCTTCACCTTCAATTCAACACATT

GTTAGCTTCTCCCTCTAAGACTTCTACAAATGGAGGGAAAGATGAGTTGGGCAGTTGCTG

ATGCCCTTGACTATAAGGGCTCCCCGGCCGACCGCTCGAAAACTGGTGGTTGGGTGCCTG

CTGCTCTTATTCTTGGGATTGAGATGTGCGAAAGGCTATCAACAATGGGGATAGCAGTGA

ACCTTGTGACATACTTAGTTGGAACTATGCATATACCTAGTTCAACCTCGGCAAACGTTG

TCACAGATTTCATGGGTACTTCCTTCCTCCTCTGCTTGCTAGGAGGCTTTTTGGCTGACT

CCTTTCTTGGTAGATTCAAAACTGTTGGAATCTTTTCTACAATCCAAGTACTAGGAACAG

CAATGCTAGCGGTGTCGACAGCATTGCCGGAGCTAAGGCCACCACCATGCCACTTTCGGA

GGAACGAACCGTGCGAAAAGGCAACAGAACTCCAAATGGGAGTACTATACTTAGCCTTAT

ACTTGATAGCCCTAGGAACAGGGGGACTAAAATCAAGTGTATCAGGATTAGGAAGTGACC

AATTTGATGAGAGTGATGATAAAGAAAGGGCTCAAATGACTGCATTTTTTAGCAGGTTTT

TCTTCTTCATAAGCTTAGGGACACTAATGGCAGTGACAGTGTTGGTGTGGGTCCAAGATG

AGGTAGGAAGAA

>Unigene31143_All - nr

ACAGAAGAAGGAAGAAGAGGAATAAACAGTTATTACAATTGGTATTACACAACATTCACA

ATTGTAGTAATGATAACATTAACAGTTGTGGTTTATATACAAGACTCAGTGAGTTGGGTT

TGGGGTTTTGGGATCCCAACAAGTCTTATGTTGTGCTCAATTTTGCTCTTCTTCTTTGGA

ATGAAGTTTTTTGTGTACATCAAGCCTCAAGGAAGCATCTTCTCTAGCATTTTTCGAGTA

TTTGTTGCCGCGTATAGGAAACGTTGTCTTGCCCTTCCTGATCATCGTGGCGTTGATAAA

GGTGTTTTCTATGATCCTCCTCTTTCTGGAGTTCTTCAATCGGTGCTCCCTCTTACCAAT

CAATATAGTTTTTTAAACAAAGGAGCTATAATACAAGAAGGAGAGGTGAGGTCAGATGGT

AGAGCAACAGCAGTAAGCAAATGGAGTTTATGCAGCATCCAAGAAGTAGAACAAGTAAAA

TGTGTGTTAAAAGTGCTCCCAATTTGGGCATCTGGTATAATCTGTTTCACATCAGCGGCT

CAGCAATCAACCTTTGCAGTCTCCCAAGCCATGATCATGAACCGAAACATAGGCCCGAAA

TTTCAAATCCCACCAGGGTCAATAACCATCATATCTATGCTAACCATAGGAGTGTGGGTC

CCAATCTACGACCGGTTCGTGGTCCCGGCCCTTAGGAAGATCACGAAACACGAGGACGGC

CTCTCCCTCCTCCAAAGGATGGGGATCGGCCTCGTTTTCTCGACACTTTCGATGGTGGTG

GCCGGGTTAGCCGAGCACAATAGAAGGGCTAATGCTAACTTGCACCACAATAACAGAGTA

TCAGTGATGTGGTTAGCCCCTCAACTAGTTATGCTAGGGCTTGCTGAAGGGTTCAATTTT

ATAGGTCAAATTGAGTTTTATAATAAGCAATTTCCAGAGAATATGAGAAGTATGGCTACT

TCTATGTTCTTTTGTACAATTGCTGGGGCTAGCTACTTGAGTTCAGCTGTGGTTAGTTTG

GTTCATAAGTTTACTGGTACCCCTGATTGGTTAACCAATGATCTTAACCAGGGTAAGGTG

GACTATTTCTACTACTTGCTTGCTCTTATGGGGGTCTTGAATTTTATTTACTTTTCTATT

GTTGCTAGGAATTACAAATATAAAGGCATTAATTATGATACTACTCATGATGATATAGAA

CTTGATCCAGTTATTAAACATTAACTAATTAATTAAACAGGAGGGATTATTGGAGGTAAT

TCTCTAGTTTAAGGTATATATAATAATATATAGTTTTATATATATATGTAATGTAATAAG

CAAAGGCTGTATTAGAGTCTAATTAGTAATTAATGTTCGGC

>Unigene49607_All + nr

CTGTTTTTGACAAGAGTGGTTGGGCAAACCAACGCTAATGCAGCAAATAATGTTAGTACA

TGGATTGGTACTGTTTATCTCTTCTCTCTTGTTGGTGCCTTCCTTAGTGATTCCTATTGG

GGAAGATTCAAGACTTGCGCTATTTTTCAGGCTATCTTTGTCCTTGGATTGGCATTATTA

TCACTATGCACATACTTATTTTTACTTAACCCAAGAGGGTGTGGAGATAAACGTACACCA

TGTAACAAGCCCTCAAACCTACATGTATCACTATTTTATGTATCACTCTACATGGTTGCC

CTTGGCAACGGTGGTTATCAACCCAATATTGCCACATTTGGGGCAGACCAATTTGATGCC

GAATTTCCAAAGGAAAGGACCTCCAAAATATCCTTCTTTAGTTACTTTTACTTGGCCTTT

AACTTAGGCTCTCTATTTTCCAACACCATATTTGCTTACTATGAAGATGTTGGTAAGTGG

GTGATTGGATTTTCGGCGTCTGCCGCTTGTGCTCTGGTGGCTTTGTGGTTGTTCTTTGGC

GGCACGCCTTGGTATAGGCAC

>Unigene30293_All + nr

CAAGGTTTCAAGTTCTTGGATAAAGCCGCAGTGATCACACCAGCAGAACACGTTGGACTG

AACAAAAAGATGTATGAACAAAAGTACTTGTGCACAGTTACACAAGTTGAAGAAGTGAAA

TGCATCTTACGACTTTTACCAATATGGGTTTGCACTATTATGTATTCTGTAGTTTACACT

CAAATGGCATCCCTATTTGTGGTTCAAGGTGCTGAAATGGACGCGACCTTAGGACCCTAC

CACATTCCCCCAGCTAGCATGTCAAGTTTCGATATCCTTAGCGTTGCAGCCTTCATATTC

ATCCATAGTCACATCGTGAACCCTTTTGTGATGAGATGGAGGAAGAAAGGTTTAAACGAG

CTTGAAAGGATGGGGATCGGCCTAATCTTTGCAATTATGGCCATGATCGCGGCAGGAGTT

GTGGAAATCTACAGGCTGAAATACGCGACAGGGAATTGTAAGGGATGTAGCGGTGCAGCA

TACGCCAGCTCACTGAGCATCTTATGGCAAATTCCGCAGTTTGTGCTGATTGGTGCATCT

GAAGTTTACATGTATGTGTCGCAGTTAGAGTTCTTTAATGGACAAGCGCCCGAAGGAGTG

AGGAGTTTTGGGAGCGCACTTTATACAACATCAATGTCTCTAGGGAATTATGCAAGTATC

ATAATAGTGACCATCGTAATGAAGATAACCACTAGGGATAACATGCAAGGATGGATACCA

CAAGACCTAAATGAGGGTCGATTGGACAAGTTCTACTTCTTATTGGCTTTTCTTACTATG

CTTGATTTTGGGGTGTTCTTGGTCTATGCAAGGGCTTATAAATATGTAGAAACTAATAAT

AGTGATTATAAGGACGAATGTATTGATGTCTAAGTGAACTTGGTATTTACAATTGCAAAT

TTAATGATTATGCGCAAGTTTCGTAATAAACAATATTTTACATGTTTAGTGAAACACG

>Unigene63092_All + nr

CTTCTTTCTCTTGTCTTCTTAAGTATCACATTTTGTTTTCTCTATCTTTCATCAATTCTT

TGTCTTTGTTCTCATTTTTCTTCACTTTATCAATGGCTATTGGTGGGGTGACCAAGAAGA

GAAATGTGAAGAAAGAGCAAGATGATGTTTACACAATGGATGGAACAGTGGATTACCGTG

GTCATCCGGCCAGTCGCCAAGATACTGGGCAATGGTTTGCGGGAAGTATTATCCTTGTAA

ATCAAGGGTTGGCTACCTTGGCATTTTTCGGAGTAGGGGTGAACTTGGTACTGTTCTTGA

CAAGGGTGCTTGGAGAGAACAATGCTGATGCTG

>Unigene71213_All - nr

CAAATACTGTCAGCAAATGGACTGGAACCGTCTACATCTTCTCCCTTGTTGGTGCATTCC

TTAGTGATTCATACTGGGGAAGATTCAAAACTTGTGTTATCTTTCAGATCACATTTGTAA

TTGGTTTGGTTTCAGTATCTTTATCAACATATCTCTTCTTACTTAGACCAAAAGGCTGTG

GAGATAGAATAACTCATTGTGGAAAGCATTCAAGCATGGAGATAGGGTTGTTTTACCTCT

CAATCTACCTAGTGGCCTTAGGATATGGAGGGTATCAGCCTAATATAGCAACACTAGGAG

CTGACCAATTTGATGAAGAGTATGATAAGGAAGGCCAATCAAAGGTGGCCTTTTTTAGCT

ATTTCTACCTAGCAATGAACCTTGGATCACTCTTCTCTAACACCTTTCTTGGATTCTTTG

AAGATGAGGGATTGTGGGCACTTGGATTTTGGGCATCAGCTGGGTTTGCCTTTCTGGGTC

TCATTTTATTTCTTGTTAGGATACCAAAATACAGGCATTTTAAGGCAACAGGCAATCCCA

TTTCAAGGTTATGCCAAGTCCTTGTTGCTGCAACTAGGAAATCAGGAGCTAAAATGCCAG

AAGGACAGAGACTATATGAAGTGGAATTAAAGGAGTGCTCAACAGATGGAAACAGAAAAC

TACTCCATACTGAAGGATTCAAATTCTTAGACAAAGCCGCATGTATCAAACCTGAGGATT

TCTACAACCAAGATCAAAGTTTTCATAACCCATGGCGTCTTTGCCCCATAACACAGGTCG

AGGAAGTGAAGTGCATATTAAGACTACTTCCCATATGGTTATGCACCATAATTTACTCAG

TAGTATTCACTCAAATGGCATCTCTCTTTGTAGAGCAGGGAGCTGCTATGAAAACTACAG

TCGCAAACTTTAGAATTCCAGCTGCAAGCATGTCTAGCTTTGACATTCTGAGTGTTGTAG

CTTTTGTTTTTATGTATAGAAGAGTTATTGATCCTCTAATGAGTAGAATAAGGAAGAGGG

AATCTGGAGGGCTAACTGAGCTACATAGAATGGGTATTGGCCTGATTATAGCAGTAATGG

CCATGGTCTCAGCGGGAATTGTCGAGTGCTACAGACTAAAATATGCCATAAAGGATTGCC

CACATTGCAAAGCACCAAATCCAAGCTCACTCAGCATTTTTTGGCAAATTCCTCAGTATG

TGTTCATTGGAGCATCAGAAGTTTTTATGTATGTAGGTCAACTGGAGTTTTTCAATTCTC

AAGCACCTGATGGGTTAAAGAGTTTTGGGAGTGCTCTTTGTATGACGTCCATCTCACTCG

GAAACTATGTGAGCAGCTTGATCGTCTCAATAGTTATGAAAATCTCAACAGAAGATCATA

TGCCAGGGTGGATACCGGGTAATCTTAACAAGGGTCATTTGGACAGATTTTACTTCCTAT

TAGCAGCTCTCACGATGATTGATTTTGTTGTCTATATATTATGTGCGAAATCATACAAAC

GTATCAAGCTAGAAGGAAAATCTGATGGAGACAACAACCAAGTTGATTATGAAGTGTAAC

ATCAGCTAAAATGTCTACATTATCAAGGCCTCAAGGTTCACTTGAATAAAAGACAAAGGT

AGCTTCCATCCACCATGAAGATGCTTTTTTAAGACAGGAAAAAACAGGAAGATTATTTGA

TGGAACAGTGACCATTGTAATTGCTTGTCAAGCTTACCTGTGATGATTGAAGGATAACAT

TAAGACCCACTTAATTCCCCCCTAAAAGGGCAGACTTGCACTACAAGTTCAAGGGAATGG

TTTTTAGCCCTTCAACTGAATGCTAGTCTATTTGCTCTAATTGTAGTAGGGACTAAGCTG

TGTATTTCAATTCTGCCAATAGCCAGCTCATTCGCATTTTGTCTTCTTCATTTTAATTCC

TAACTTTCATATGCTGGCTTTTTCCAAGATTACAACATCTTAAGAGCAAGGCAAAGTTCA

AACCAACTTGAACCTGCCCTTACAAATCACTAGGCTTTCATGATGTAGTTGGTTTCAGAA

ATGGCAGCCCGTCCAATTATCAGAAACCACCCCTCACAGCACAAGTACTCAAAA

>Unigene34485_All - nr

AATTGGGGTTATGGAATATGTGCATGTGCAATTGTGGTATGCCTAGCTGTGTTCGTTTTG

GGTACTAAACGGTACCGTTTCAAGAAGCTAGTTGGTAGTCCACTTACTGAAATAGCAGCA

GTTTTTGTGGCTGCCTGGAAAAAGAGAAATTTGGAACTTCCTGCTGATTCCTCACTACTT

TTCAACATTGATGACATGGCACATTCTACTTTGAAGAAAAAGAAGAAGCAGAAGCTCCTA

CGTAGCAAGCAATTCAGATTTTTGGACAAAGCCGCGATAAAGACAGCAAAAATGAGTGAA

GACATGAGCTCAGTAAGCAAGTGGAAGTTAGCAACACTAACAGACGTAGAAGAGGTAAAG

ATGATATTAAGAATGCTTCCAATTTGGGCCACAACCATTGAATTTTGGACCATCCATGCC

CAAATGACAACATTTTCCGTATCACAAGCCACCACAATGGACCGTCACTTATTAACTTCC

AATTTCCAACTCCCACCCGCCACCATGACCGCCTTCCTCATCGCCAGCATCCTCCTAACC

GTCCCCATTTACGACCGTCTCGTGGTCCCCACAGCCGCCAGACTCTTCAGGGACCCACAA

GGGCTCACCCCGCTCCAACGAGTCGGGGTAGGCCTGTTCTTAGCCACCGTGGCAATGGTG

GTGGCCGCCCTAACCGAAATCAAACGACTACACGTGGCTGAATCTAACGGGCTGGTGAGG

GACCCGAACGCGGTCCTACCAATGACGGTTTTCTGGCTGGTCCCACAGTTTATATTAACG

GGTGCAGGGGAAGCTATGATATATGCAGGGCAGCTTGATTTTTTTCTAAGGGAATGTCCA

AAGGGAATGAAGACTATGAGTACTGGGTTGTTTTTGAGCACGCTTTCATTAGGGTTTTTC

TTTAGTTCAGTGATTGTTACGATAGTGAACTCGGTAACAGCGGATTCTAAACCGTGGTTA

GCGGATAATCTTAACCAAGGAAGGTTGTATAACTTCTATTGGCTGTTAGGTGGGATTAGT

TTAGTCAATTTTGGGTTGTTCTTGTTGTGTGCAAAGTGGTATGTGTATAAGGAAAATTGG

GTTGATGATCAAGGGTCCTTACAGTTTGAATTGGATGAAATACCTGCCCCAACCTCCCAT

TGAAATTGTAAACAAGATATTTATGTATAACTACGTTTGTTGGTCAAAGAATATACTTAT

AATAAATAAAATGGAC

>Unigene85390_All + nr

AAAGAAAGAGCTTGTACAAGAATTGGCCCTTGATTTCCTCTATTATTGTGTATTGCATTT

TCTCATTTCATGATATGGCTTACACTGAGATATTTTCATTATGGGCAGTTAGCCCAAGAA

CACTAGGGGGATTAGGCCTTTCATCCCAAGATGTTGGAGTTGTTCTTTCTATCACAGGTG

TTGGCATGTTGATATTCCAACTCCTAATATATCCACAATTGGACAAATATTTCGGGCCAA

TTATGTCAGCTCGAATTTGTGGGGTATTGTCAATACCGATATTACAGAGTTACCCTTTCA

TGGCATTATTATCAGGGTTGAGCCTTCATTTCTTGTTAAATTCTGCTTCTGTCCTAAAGA

ACTGTTTATCAGTAAGTGACAAACAATATCATTAAGTTCATTCACACACA

>Unigene23477_All - nr

CCGTTTTTCCTCAAGGATTAGTAGGATTTGCTGGAGTATTCATGGCAGTAGGTCAAGTGG

AGTTTTACTACAAGTAATTTCTTGAGAGCATGAGGATCATTGTTGGATCCATCTTCTTTT

TAGGCATCAGAATGACAGAATCTCAAACTACTTAAGCAGTTTGTTAATTTCCATAGTTCA

AACGACGACAAACATTAGGTGATTGGTTACCAAAGGATCTTAACCAAGGGAGATTAGATT

ACTTCTGTTACTT

>Unigene71982_All + nr

CTCCCAAAACAACTCCCACATTTCTTTCTTTTCTTCTTCTTCTTCAATCTTCATAACTTT

TATTAATCTTTTTCTTGCACGCCAAACATTGGCTCATTCTTCTTAACAACATTATTTCAA

TGGCTTGCATGAATATTTTCCAAAAGGAAAAAATAATAGATGAAAGTGAAGAAGTAAAAT

ACACTCTTGATGGAAGTGTGGATCGACATGGGAAACAAGCAATTCGGAGTAAAACTGGAA

GATGGGGCCTCGGTATCCTTATCCTAGTGAATCAAGGGCTAGCAACAATGGCATTCTTTG

GAGTAGGAGTGAATTTAGTGTTGTTTTTGACAAGTGTGGTAGGCCTACAGAATGCTGATG

CAGCAAATGAGGTCAGCAAATGGACTGGTACTGTTTACCTCTTCTCCCTTGTTGGTGCCT

TCCTTAGTGATTCTTATTGGGGTAGATACAAGACTTGCACCATTTTCCAAGCTATCTTTG

TCCTTGGATTGGGTTTGATGTCCCTATCATCATACTTATTCTTGCTTAAGCCTAAAGGGT

GTGGGGATGAACAAACTATATGCACAACCCACTCAAACCTCCATATATCACTTTTCTACA

TATCATTATACATGGTGGCCCTCGGCAATGGAGGTTATCAACCAAACATCGCGACATTTG

GAGCAGACCAATTTGATGAAGAACATCCAAAGGAAAGCGGCTCCAAAATCGCCTTCTTTA

GTTATTTCTACTTGGCCTTCAACTTAGGTTCATTGTTCTCCAACACCATATTGGCTTATT

ATGAGAATGCTGGGAAATGGGCTCTTGGTTTTTTGGCTTCTGCTACTACTGCTCTCATAG

CTTTGTTGATGTTTCTCGGCGGTACGCCTTTTTATAGGCACTTCAAGTCTCAAGGTAATC

CAATTACAAGGTTTTGCAAAGTGTTTGTTGCTGCCATTAAGAAATGGAAGGCTCATGTTC

CTCCTCAAGGTCAAGGTTTGTATGAATTGGATGCCAAGACTAACTCTAAAAATGGAGCAA

GGAAGATCTCACACACTGAAGGATTGCGGATCTTGGACAAAGCTGCAGTGATCAACGAGC

TTACGAAATGC

>Unigene61016_All - nr

AATGCACTGAGATCTGGCATCTGGGTGAGAAAGCTAGCTTCATACCCGGATGCCAGATCT

CAGTGCATTCTGGCTGCTAATCCAGTACTGCCTCATTGGCATTGCTGAAGTCTTTTGCAT

TGTGGGGTTGCTGGAATTCTTATATGAAGAGGCACCTGATGCCATGAGAAGCATAGGATC

TGCCTATGCAGCGGTCGCAGGAGGTCTAGGTTGCTTTGCTGCAACAATTCTGAACAACAT

TGTTAACTCTATCACAGAAGATAAAGGAGGAGGACGCCCTTCTTGGTTATCCCAGAATAT

CAATAATGGCAGATTTGATTACTTATACTGGCTGCTTACAGGACTCAGTATAGTCAATTT

CTGTGGTTTCCTTTATGCAGCCAGGAGATACAAATATAGATCAGTCTTCCAAGTGGTACA

ATGATAGATGAAAGAAGCACAAGCCTTCAACACAACCTTAAATCTAAGAATATAATTGAG

TCCTTCCAAGTCATTCTTCCAAGTCCTTCCATAGCTCCCATGCAATCTTGAAAACAGAAA

CTTTTTAAAACTCTTTGTTCAAGATGAACAATAATCATGAAATTACCAAATTGAGTTGTT

GCAATTGTACTCTGATGATTGATGAGATTAGTTATAAAAAAAAAATCTACTTTACAGCTC

TTGTTATGCTGTCAAGATATTAATATTTGCACCAA

>Unigene34465_All + nr

GAATTATTAATCAAAGTTGAAATGAAAACAGCATAAATGACAAGAATAAATATTTTGAGA

AATAAGGGGGTACATTCTGTGCCAACCATCAACTAACAAACCTTCATATCTCTGCACTAA

AAGAAACAGACTTTCATATTTCAACATAAATGAGATTCATATTGCATAACAAAAAAAAAA

CCCATACATCATCCTCAAGACCTCAAATCCATTGTAGTTGCACCACATGAATAAAATGTA

TATAGAAGCAGAAATTTGAGTCTGAAATAATGATGCTAATCAAGTAAATAAACAAGGTAA

TAAGAAGGAAGTGAGAATATTCACACAGTGTGTGTGTGAGGCAGAAATGGGTAGTGCAGA

AATCCGGTCACCTCAAATTCAAGTTCTTGGAACACCATCTACTCCTTCCAAAGGCATGGA

CATGTCTAAGAGAAAGAAACTGGGAATCTATTTCATGGAGTCAGACGATAGACGAGGAGC

ACTTGGTGGAGGTTATACTGTAGGGAGTACACCAGTTAACATTCACAGGAAGCCATTGTC

TGAAGATGATCTCTCTAAGACCGGTGGTTGGGTTGCCGCCTTTTTCATCTTTGGAAACGA

GATGGCTGAGAGAATGGCTTACTTTGGTCTATCAGTGAACATGGTGGCTTTTATGTTCTA

TGTTATGCATCGGCCCTTCACTAGTTCGGCCAATGCTGTCAATAATTTCCTTGGAATATC

ACAGATTTCATCCGTGCTTGGTGGCTTTCTAGCTGATGCCTATCTTGGTAGATATTGGAC

TATTGCAATTTTCACAACCATCTATCTTATGGGGTTGACAGGGATAACACTATGTGCAAC

AATGAAGTCGTTTGTGCCGAACCAAGATTGCAGTCAGCTAGCAGTTCTTGTGGGGAATTG

TGAAGCAGCAAAACCATGGCAGATGCTTTACCTGAACACAATTCTCTATATAACCGGATT

TGGAGCAGCAGGCATAAGGCCGTGTGTCTCCTCTTTTGGAGCTGATCAATTTGACGAAAG

AAGTCCAGATTACAAGGCTCACTTGGACAAGTTCTTCAATGTGTTCTATCTGTCTGTCAC

AGTTGGTGCTATTATAGCATTCACAGCAATTGTTTATATCCAGATGCAGCATGGCTGGGG

ATCTGCATTCGGATCGTTAGCATTGGGGATGGGCATATCAAATGCGCTCTTCTTCATTGG

CACTTCCACTTACCGCCACAGGTTGCCAGGAGGCAGTCCTTTGACGCGTGTTGCCCAAGT

TCTAGTAGCAGCCTTCAGAAAAAGAAATGCTTCTTTTTCCAGCAGTGAGATCATTGGCCT

ATATGAACTTACAGGGAAAAAATCTGCTATTAAAGGTAGTGCGAAAATTGCTCACACCAA

TGACTTCAGATGCTTGGACAAAGCAGCACTAAAGTTGCCAGAAGATGGCCCAGATCAAAG

CCCTTGGAGGCTTTGCACAGTTACTCAAGTGGAGGAAGTCAAAATCCTGATAAAACTCTT

GCCTATTCCGGCAAGCACTATTATGCTCAGTCTAATCTTAACAGAGTTCCTGACTCTGTC

TGTCCAACAAGCATACACGATGAATACCCACATAGGTCATCTCAAAGTCCCAGTTACCTG

CATGCCTGTTTTTCCTGCCCTAAGCATATTTCTTG

>Unigene61016_All - nr

AATGCACTGAGATCTGGCATCTGGGTGAGAAAGCTAGCTTCATACCCGGATGCCAGATCT

CAGTGCATTCTGGCTGCTAATCCAGTACTGCCTCATTGGCATTGCTGAAGTCTTTTGCAT

TGTGGGGTTGCTGGAATTCTTATATGAAGAGGCACCTGATGCCATGAGAAGCATAGGATC

TGCCTATGCAGCGGTCGCAGGAGGTCTAGGTTGCTTTGCTGCAACAATTCTGAACAACAT

TGTTAACTCTATCACAGAAGATAAAGGAGGAGGACGCCCTTCTTGGTTATCCCAGAATAT

CAATAATGGCAGATTTGATTACTTATACTGGCTGCTTACAGGACTCAGTATAGTCAATTT

CTGTGGTTTCCTTTATGCAGCCAGGAGATACAAATATAGATCAGTCTTCCAAGTGGTACA

ATGATAGATGAAAGAAGCACAAGCCTTCAACACAACCTTAAATCTAAGAATATAATTGAG

TCCTTCCAAGTCATTCTTCCAAGTCCTTCCATAGCTCCCATGCAATCTTGAAAACAGAAA

CTTTTTAAAACTCTTTGTTCAAGATGAACAATAATCATGAAATTACCAAATTGAGTTGTT

GCAATTGTACTCTGATGATTGATGAGATTAGTTATAAAAAAAAAATCTACTTTACAGCTC

TTGTTATGCTGTCAAGATATTAATATTTGCACCAA

>Unigene49930_All + nr

TGCATCCTGAAAAATAGTGTATTTCATATATCAAATGTCTTTTACTTTGTTACTTTTCCA

AAATTTATTAGGAAATGAGATATTTGAAAAGCTAGGAGCAATTGGTACCCTGTTCAACCT

GTTAATGTTACTTGACAACAGTATGTATTCAACATGAAGACTCACAGCAACAACTTGATA

TATATATCTTCAATGGCACCACCAACTTTGCAACTTTACTAGGAACCTTCCTTTCTGACA

CCTACTTTGGCCGCTGCACCACCTTGGGCTTCGCATACATCACCTCTTTTTGGTACTTTA

TGCTTCTTCTACTATTTCCACGTGCACAATACATGGAGCAGACAATATACAGCAATACAA

CATGGTTTAGGGAGGGTCGGGTGTTTAAAACTGCACATAGTTTCAGTTCTCATTTTTGTC

TAGCTACATGCTGAACTAATCTTTACTGTCAGCATTTTATATTGATGCCATTGTATATGG

TGGGGACCATGAAG

>Unigene54471_All + nr

CCAACAAGGCAGTCAAATGAACACCCAACTCACTTCCTCCATAAACATCCCCCCAGCCTC

ACTCCAATCCATTCCTTACCTCCTTCTCCTCTTCCTCGTCCCACTTTACGACAAACTCTT

CGTCCCTTTCATCCGCCGCTTCACGGGCCACCCCTCCGGCCTCTCCCCTCTCCAACGTAT

CGGGTCCGGCCTCTTCCTCGCCACCTTCTCAATGATCTCCGCCGCCCTCATCGAAAACCA

CCGTAGAAACCACCAACCACCTCTCTCTATCTTCTACATCACCCCACAATTCCTCATATT

TGGACTATCCGAAATGATGACTGCCGTTGGACTTATTGAATTTTTTTACAAACAGTCACT

TAAAGGAGGGTCATTACAGTCTTTTCTTACGGCGATTACTTACTGCTCTTACTCTTTTGG

GTTTTACCTTAGCTCTGTGTTGGTTACCCTTATTAATAAACTCACTTCTTCTTCTAAGAC

TAATTATCAAGGGTGGTTAAGTGATAATGACCTTAATAATGATAGGTTAGACTTGTTTTA

TTGGTTGTTAGCTGCCCTTAGCTTCTTAAACTTCCTTAATTACCTATTTTGGTCTCGTTG

GTACTCTTTTAATCCTTCTCTTTTGCTCTCTTCTCAACCACATGTTTCTCCTCATGATGA

CCTCACCCCTCCTCCTACCAATAATACACATTCATTAATTTAATTAGATTATAATTAAAT

TAAGGAAAATTTGTTAGAAACCACCTTATGAATTAAATTTTTCGTCAAAAACCACCTTTT

AAATTATTATTTTGTTTGTAAGAAGCCACCTTTTTTGGATTTTTTTTTTGGTGAGGAACC

ACCTTTGGTTGAATTTCGGGTTTCGATTTTGGGTTATTTTTGGCGTGGTGGTTCAGGGGA

GGAAAAGGGACGGAGACGGCTTGGGGAGGTACGAAGGGGGTTAGCCGAAATGGCGGAGGG

AGGTTCAATTTAAACACTGAAATTAGGCTAAAGGTGGTTCTTGACAAAAAAAATTAAAAT

AAGGTGGCTTCATAAAAATTAAAATTTAAGGTGGTTTTTGACAAAAACAAAATGTTTTAT

AAGGTGGTTTTTAACAAATTTTCCTTAAAATTATTTATTTTTGTAAAAAAAACACCTTTG

AAATTATTGTTATTATTTTTTTTTGGGTTTCAATTTTGGGTAGTGATACAGGGGAGGGAC

AGACTGGTCTGTGTTCGCCAGAACGACAGAAGAGTTCGATTTAAACACCAAAATTCAGCC

AAAGGTGTCTCTTTAAAAAAAAATTAAGATTAGG

>Unigene54452_All - nr

AAAAAAAAAAAAAAAAAACTCATCTTCCACAATAACATAGCAGAGCAGTACATCACCTAA

CAGTCTAACACAACCAGTAAGCAAAAAAATTAGCCTTACATATCAATCAATCAAGCAGTA

CATCACCCTAAAAAAATTAGCCTTACATACCAAAAAAAAAATAAAATAGAGCAATGGCAT

CCATATACAAAGGGCTTGAAGCAAAAAGATTTGCTCTTCAAGTTGATGCAGAGAACAAGG

CCATAAGCTTCCCACTATTTTCAAAAGCAAAACCTCACATGCAAGCATTTCATTTATGCT

GGATACAATTCTTCTCTTGCTTTCTCTCCACATTTGCTGCTCCACCTCTTCTCCCTATCA

TTCGTGATAATCTCAACCTTACAGCCAATGACATTGGTAATGCAGGGGTTGCATCTGTGT

CAGGGGCAATCTTTGCTCGTGTTGCCATGGGTGTAGCCTGTGATTTAGTCGGACCACGAC

TAGCCTCTGCAACCCTCACCCTCCTAATTGCACCTGCAGTATATCTATCAGCAGTGGCTG

ATTCTGCCACTGGTTACTTTCTTATGCGCTTTTTCACAGGCTTTGCTTTGGCCACTTTTG

TGTCAACTAAATACTGGATGAGTTGTATGTTCTCCCCGAGAGTAGTTGGTCGAGCCAATG

GGCAAGCAGCAGGGTGGGGAAACCTAGGAGGAGGTGTAGCCCAACTCGTAATGCCTCTGG

TCTACAGTGTAGTCCTTAAAACAGGAGCAACGCCATTTGCAGCTTGGAGGATCCCATTCT

TCATCCCTGCTCTCTTCCAAATGTCCTCAGCATTTGCCGTGATGCTTTTTGGCCAAGACC

TGCCAGATGGAAATTTCCACCAGCTACAAACGTCAGGTGTGACATGTACAAGGACAATGC

TTCCAAAGTCTTGTATCATGGAATCACCAACTATAGGAGCTGGATCACTGCTCTGGGCTA

TGGGTTTTGTTTCGGGGTGGAGCTGGCAGTAAATAACATAGTTGCCTACTACTTTTATGA

CCGGTTTAATCTGAATCTCTATATTTCAGGAGTAGTTGCAGCCAGTTTTGGAATGGTCAA

TCTTTTTTCCAGGCCTATAGGTGGTGGGTTGTCGGATTTTTCAGCAAGAAAATTTGGAAT

GAGAGGAAGATTGTGGAGCTGGTGGATTGTGCAGGCCCTAAGCGGTGTTCTGCGTATTGT

TCTCGGATGGATCAACACCTTAGGCGCTTCAATAGCAGTAATGATCGTCTTCTCAGTGTT

TGTGCAAGCAGCCGAAGGACTCACGTTTGGGGTTGTTCCTTTTGTCTCC

>Unigene44258_All - nr

GTTTTGAAATTAAATGAGCAGCAGATTATTGAACTTCTAACCAGAATAAAGGGGGGGAAG

GGAGAAAAATTACAGTCAAACATGCAGATAGGCGAGATAGCAGTTCATGCAATGTGGAAA

CAGCTACTCCGTACTAGTCCGTAGTTTAATTTGCTCATTTGCGATAATAAAAGGGCTTCT

TGATTGGACTGTCCATGTTCCTAGAATGCCGGAACCAATGAATTTGATTCCTTTCAAATT

ACCTTATTTGCACAACTAGGCTTCTCTCACATGGACAGAGACAAAAGGATAGAATATTCC

AACCCATAATTAGTACTGTAGTTGTGTATGTCGAAGTTCCCTTCATATAACTAATTGTTA

TTCCAGAAGAATGTTCCAGTTTGATCCATTCCACAGCTATGCTGACAGTCTATGATTCTG

CTTCCATTAGGCTTTTACAGAAGATTTTGCCTGACAAGCCTTTAATCCTCCTTACTGAGC

TATCCCTGCAAACAATTTTGATACATATCTTCATCTCTTCAGATCTATGGGAGTGGTTGC

TGGTATGGCAGGAGCTGGTGGAAATGTAGGTGCAGTCATACTTCAAGTGATTTTCTTTAG

GGGATCTCAATTCTCGACGGAAGAAGGAATAACATACATGGGTGTTGTGATCACAGCATG

CAGCTTCGGCATAATGACCATATACTTCCCACAGTGGGGAG

>Unigene34113_All - nr

CCCCCCCACAAAAAAAAAAAAAAACAAAACTTGCCAAATACAGGCAAAACATAAAGAGAG

ATGGCATCAGTAAATGAAATGAGCGAGCCTGAACCCAAAAAATTTGATCTTCCAGTTGAT

AAAGAGAACAAGGCCATAATCCTGCCAATATTTTCAGTAGCAAAACCTCACATGAGGGCA

TTTCATCTATGTTGGATGCAATTCTTTACCTGTTTTCTGTCCACTTTTGCAGCTCCACCC

CTCCTTCCCATCATTCGTGACAACCTCAACCTCACAGCCCGGGACATCGGCAACGCAGGG

GTCGGGTCTGTGTCGGGTGCAATCTTTGCTTGTGTTGCCATGGGTGCTGCCTGTGACATA

GTCGGGCCACGACTAGCCTCTGCCACCCTCGTCCTCCTCACTGCACCTGCAATCTACCTG

TCTTCTATGGCTGATTCAGCCATGGAATTCTTTCTGATGCGGTTTTTTATAGGATTCGGG

TTAGCCACATTAGTCCCAACTCAATACTGGATGAGCTCCATGTTTTCCCCAAGAGTACTG

GGGCAAGCAAACGGGCAAACGGCCGGGTGGGGAAATCTAGGAGGAGGAGTTGCCCAGTTC

CTCATGCCCCTTGTCTACAGCCTAATCCTTAAGACAGGGGCAACTCCGTTCTCTGCATGG

AGGATTTCCTTCTTCATCCCCGGTCTTATGCAAATGTTCTCAGCATTTGCAGTCATGCTT

TTCGGCCAAGATACACCTGATGGAAATTTCCACCAGCTGCAGAAGTCTGGTACTATGCAC

AAGGACAATGCTTCCAAGATCTTTTACCATGGAATCAGCAA

>Unigene5588_All - nr

GGGATCTCAATTCTCAACGGAACAAGGAATAACATACATGGGTATTACGATCGCAGTATG

CAGCTTAGGCATATTGTCCATATACTTCCCACAGTGGGGAGGTGCATTTTGTGGTCCTAA

ACCATGTTTCAATGAAGAAGATTATTACTTAAGGGAATGGGACGCAAAGGAACAGGAACA

AGGTCTTCATCTGTCAAGCATGAAATTTGCTGAAGGCAGTAGAAACGAGAGAAGCACCAA

GAAAATGTCAGCAACAACAGAACAAGAGATGAGCACAACCAATCAAGTCTAAGCTAATTC

AGTACTTACAGCAGAGAGAACACCGGATCATGTCCAATTGCTGCACTGTAATATGTTTTA

TACTATATGAGCTTCTCAAAAGAAATGAACTACGGAGAAAATTTTCCTACTGCAGCTGCT

TCCAACAGTTTCCTTTTCCTTTCTGCTTTTTTGCATATATGTCATGCTTTACAAGTTTTC

GATAACAAGAGTACAACTGTACAAGAATTGTATCTATTTTTTCCCATCCTTGATTTGATA

TTTAAGTTTTTTAATTATACTCCCAAATGGAGTCCTTGTAAGCAAAGCCTTCAATTGGAT

CTTCACATCCAAGATGTATGCCAATAGGCCAGTGTACAAATAAAATCCATGAGTTGAACA

AAAGATAATGGAATTGCAAAGCACAAATACAGACTGTTTACATCACGTCTCATCATTAAT

AAACACTAAAATCTTTTATTGGCATCT

>Unigene39295_All - nr

GCCCCGAGAGTGGTGGGTCAAGCAACCGGGCAAGCAGCCGGGTGGGGCAACCTAGGAGGA

GGAGTGGCCCAGCTCTTCATGCCTTTTGTCTACAGTGTTATCCTTAAAACAGGGGCAACC

CCATTCGCAGCCTGGAGGATTGCCTTTTTCATCCCTGCTCTTTTACAGATGTTCTCAGCA

TTTGCAATTATGTTTTTTGGCCAAGACATGCCTGATGGAAATTTTCAACAGTTGCAGAAG

TCGGGTGACATGCACAAGGACAATGCTTCCAAG

>Unigene80102_All - nr

GGTTGTTCCTTTTGTCTCCAGAAGGTAACTTCTAAATATTTTAATTACTGGGTCAGCTAA

CAATTTAATTTTATGTTATATGTTCATTAAGCAATACAAATATCTCATTCTCAAAGTGAA

GCTGCCTTTCTAAGGGCTTCAATAGAACAAATCTGTATTTTCTTAAAAACAAATCTGTAT

AATGATTCCCTTCAGGTCTTTGGGAGTAGTTTCTGGGATGACAGGAGCTAGTGGAAATGT

GGGTGCTGTCATAACTCAAGTGATTTTCTTC

>Unigene136150_All + nr

GAAAGACAAAAAAAAAAAAAAAAAGGTTTGGAAAAATGAATGTAGAGTCGTCGTTGGAAC

CCAAAAAGTTTGCTCTACCAGTAGATGGAGAGAACAAAGCAACAGTATTGCCACTATTAT

CAATAGCAAAGCCTCATATGCAAGCATTTCATTTAAGTTGGTTTCAATTTTTTTCATGTT

TCTTAGCCACATTTGCTGCTGCACCCC

>Unigene132777_All - nr

GACGTAGGGGCAGTGATACTTCAACTGATATTCTTCAGAGGATCAGAATTCTCAACAGAA

CAAGGAATAACATACATGGGTGTTATGATCACAATATGCAGTGTTGGGATATTATCCATA

TACTTCCCACAATGGGGAGGTGCCTTTTGTGGCCTGAAATAATCACTACATATATACCAG

ATGATTCCGCGTTTATACCGAACTGCAATTCTCCGTTTATGGCGGTGCTATATATTCTTG

TTGAACTGTGGATAGTACAAATGACATGTTTTGTTTTGTCCCCGACAATCAAGTAGTTTC

TGAATGGAGGGTAGTGGAGCAGTTGATGATCTGATCTGATTGAATTGAATTGGTAGTGGA

GCAAC

**Sequencing of PCR products (10)**

>PCR product of Unigene pair 3

TTGTTCTTGGGGTGGAAGTGATGGAAAATATGGCGTTTTTAGCAAATGCA

AGCAATTTGGTGATGTATGTATCAAAAGACATGCATTTTACACCGTCAAA

ATCATCAAACACCGTTACCAATTTCATGGGCACTGCTTTTCTCCTTGCTT

TGCTCGGTGGTTTCCTCTCTGATGCCTTTTTCACCACCTTTCAAGTCTAC

CTTACTAGTGCTGCCCTTGAGTTTTTGGGACTAGTATTGTTGACGATTCA

AGCATACAAACCGTCCCTACAACCACCAAAATGCAACCCAATGGACACCA

CAACAGGAGGAGGGTGCAGGGGACTTGATACTTCTGAAGCAGCAATGTTG

TACATAGGCCTATACCTAGTGGCAGCCGGTGTTGGAGGGATAAAAGGGTC

ATTACCAACTCATGGCGCGGAACAATTTGATGAGAGCACCCAACAAGGGA

GGATAAAAAGATCTACCTTCTTCAATTACTTTGTGTTCTGCCTATCGGCT

GGTGGTCTCATTGCAACCACGTTTGTGGTGTGGGTCGAGGACAACAAGGG

TTGGAAATGGGGCTTCCTCATCTCCA

>PCR product of Unigene pair 4

GAACAACCTGATAACGTACGTGATAAATGAAATGCATTATCCGTTGTCAA

AGGCAGCGAATATCGTCACCAATTTTATTGGCACCATTTTCCTCCTCTCC

CTCCTTGGTGGTTACCTCTCTGATTCTTTTCTTGGCAGCTTTTGGACCAT

GCTCATCTTTGCCTTCGTCGAACTTTCTGGATTCATCCTACTATCAGTGC

AAGCACACCTACCACAACTAAAACCACCAAAATGCAACATGCTAGAAGCA

GATAAAGTATGTGAGGAAGCAAAAGGGATAAAAGCAGTCATATTCTTTGC

AGCACTTTACTTAGTAGCCTTAGGAAGTGGTTGTGTAAAACCTAACATGA

TAGCTCATGGTGGTGATCAATTCAACTCTAACCAATCTAAACAACTCTCC

ACCTACTTCAACGCCGCCTACTTCGCCTTCTCCGTTGGTGAGCTCATCGC

CCTCACCGTCCTCGTTTGGGTCCAAACCCATTCGGGTATGGATATCGGGT

TTGGTATCTCTGCTATTGTCATGGCTATGGGCTTGATTTGC

>PCR product of Unigene pair 5

AGGCTGAGCTACTTTGGAATGGCAACGAATATGATAATATACATGACGAA

AGTGATGCAGCAAGATCTGAAAACAGCGGCAAACAGTGTGAATTTATGGT

CGGGAGTCACCACCGTCATGCCTCTTGTTGGGGGATTTCTTGCCGATGCT

TATGCTGGCCGCTACTTCATGATTCTCTTCTCTACTTTTCTTTATGTCTT

GGGCTTAAGTATATTAACCATGACACAATACATCCCCAGTCTTAAACCAT

GTGGCGCAACGAAAAACACGTGCGACCACGTGAGCAAGGTCCACGAGGTG

GTGTTCTTCCTAGCGGCCTACCTCGTGGCCCTTGCGACAGGCGGCTACAA

GCCGTGCCTGGAGAGTTTCGGGGCCGACCAATTCGACGACAACCATTCAA

AAGAAAGGAAACAAAAGATGTCATTTTTCAATTGGTGGAACATTGCTCTT

TGTGGTGGCCTCTTTTTTGGGGTTACATTGATTGTGTATGTTCAAGATTA

TGTTAGTTGGGGTGTTGGGTATCTTATACTGACCGTTGCAATGGGGACAA

CTGTCATTGTGTTTGTTCTTGGGAGACCGTTTTACCGTTACAGAAATGCC

AATGGTAGCCCTTTTACT

>PCR product of Unigene pair 6

ATATCACCACCACGAGAACACGTTGGACTGAACAAAAAGATGTATGAACAAAAGTACTTG

TGCACAGTTACACAAGTTGAAGAAGTGAAATGCATCTTACGACTTTTACCAATATGGGTT

TGCACTATTATGTATTCTGTAGTTTACACTCAAATGGCATCCCTATTTGTGGTTCAAGGT

GCTGAAATGGACGCGACCTTAGGACCCTACCACATTCCCCCAGCTAGCATGTCAAGTTTC

GATATCCTTAGCGTTGCAGCCTTCATATTCATCCATAGTCACATCGTGAACCCTTTTGTG

ATGAGATGGAGGAAGAAAGGTTTAAACGAGCTTGAAAGGATGGGGATCGGCCTAATCTTT

GCAATTATGGCCATGATCGCGGCAGGAGTTGTGGAAATCTACAGGCTAAAATACGCGACT

GTGAATTGTAAGGGATGCAGCGGTGCAGCATACGCCAGCTCACTGAGCATCTTATGGCAA

ATTCCGCAGTTTGTGCTGATTGGTGCATCTGAAGTTTACATGTATGTGTCGCAGTTAGAG

TTCTTTAATGGACAAGCGCCCGAAGGAGTGAGGAGTTTTGGGAGCGCACTTTATACAACA

TCAATGTCTCTAGGGAATTATGCAAGTATCATAATAGTGACCATCGTAATGAAGATAACC

ACTAGGGATAACATGCAAGGATGGATACCACAAGACCTAAATGAGGGTCGATGGACAAGT

CTACTTCTATGCTACCCC

>PCR product of Unigene pair 7

ATCTTTGTCCTTGGATTGGCATTATTATCACTATGCACATACTTATTTTT

ACTTAACCCAAGAGGGTGTGGAGATAAACGTACACCATGTAACAAGCCCT

CAAACCTACATGTATCACTATTTTATGTATCACTCTACATGGTTGCCCTT

GGCAACGGTGGTTATCAACCCAATATTGCCACATTTGGGGCAGACCAATT

TGATGCCGAATTTCCAAAGGAAAGGACCTCCAAAATATCCTTCTTTAGTT

ACTTTTACTTGGCCTTTAACTTAGGCTCTCTATTTTCCAACACCATATTT

GCTTACTATGAAGATGTTGGTAAGTGGGTGATTGGATTTTCGGCGTCTGC

CGCTTGTGCTCTGGTGGCTTTGTGGTTGTTCTTTGGCGGCACGCCTTGGT

ATAGGCACTTTAAATCACAAGGCAATCCGATTTCGAGGATTTCCAAGGTG

TTTGGAGCTTCCTTAAAGAAATGGAAGGCTCATGTTCCTCCTGAAGTTGG

CTTGTATGAATTGGATGCCAAAGCTTATTCCAAGACTGGGTGCCGTAAGA

TCTCCCATACACAAGGTTTCAAGTTCTTGGATAAAGCCGCAGTGATCACA

CCAGCAGAACACGTTGGACTGAACAAAAAGATGTATGAACAAAAGTACTT

GTGCACAGTTACACAAGTTGAAGAAGTGAAATGCATCTTACGACTTTTAC

CAATATGGGTTTGCACTATTATGTATTCTGTAGTTTACACTCAAATGGCA

TCCCTATTTGTGGTTCAAGGTGCTGAAATGGACGCGACCTTAGGACCCTA

CCACATTCCCCCAGCTAGCATGTCAAGTTTCGATATCCTTAGCGTTGCAG

CCTTCATATTCATCCATAGTCACATCGTGAACCCTTTTGTGATGAGATGG

AGGAAGAAAGGTTTAAACGAGCTTGAAAGGAT

>PCR product of Unigene pair 8 (upper band)

GAGGATGCTGGCGATGCATTCGCG

GCCGGGGCGGTCGTCGCCATTGGCGATCTTGACCCCGGTCTGGCGCACGT

AGCTGTCCCATTCGCCGGCGGTGATATGGAAGCGGCTCATGGCGAACGGT

TTGGCGAAGGTCACCTCATGCATCGGGCCTTCGTCGGGCTCGCGGCCGAC

TTCGTCTTCCGGCGTGCCCATGGTGAAGGTGCCGGCGGGCAGCACCACCA

TTTCCGGGCAGTCCTTGCAGTCTTTGAACACCTTGCCCGGTTGTGGGGCG

GCGGCCTGGGCCAGGCCGGGCAGCAGCGCGCCGCACAGGGCGGTGAGTGC

CAGTGCGGTGAGGGGTTTGAGTTGGGATGAAATCATGGAGGCATCTCGTT

CAAAGGGAAAAGGGGTCATAACCGCTGGCTCAGCAGGGTCATGAAGCGCT

GGATCTCATCGGAGTTGTTAAGCAGGCCCGGTGAGGTGCGAATCACCGGG

CCAACGTCACGGTCCACCGCGTCCACCACCACGCGGTTTTTCATCAGGTA

GGCCGCGACGGCATCGCTGTCCTGATCCTTGACTCGGAAAAAGGTAAAAC

CCGCTGACAGCTCGTGGCTGCGCGGCGTGACCAGTTCGATCTGCGGGTGG

GCCAGCAGTTGGTCCTTGAGTTCGGTGTTGAGGGCATGGATACGCGCTTG

CACCGGCGCCTTGCCCAGTTGCAGGTGCAATTTGAAGGCTTCATCGGCGG

CCCAGCGATGTTCGAAGGCGTGGTAGCCACCGGGCGTCATGGTCGTGGCG

AAGTCCTTGTCTTCGGAGAAGGTCGGAATCATCGGTGTGACGTATTTGTT

TTCGGTCTCGCGGGCGCACACCAGGCCGGTGCCGCGCGGGCCGAACATCC

ACTTGTGGGTGCCGGCGATGAAGAAGTCGCAGTGCATAGCGGGGAAGTCG

AGGTCTTCCACGCCGAAGCCGTGCACGCCATCAACGATATAGAGCAGGCG

ATCTGGCTCGTCACGGTTGCGGTTGTGTTCGTCCACCAGCTTGCCGATCT

CACCGATGGGCAATTTCACGCCGCTGCCGGACTGCACCCAGGTCATGCCC

AGCACGCGAGTGTTGGGGCGAATATTGCGCTGGATATTGCCCAGCACCTC

GTCGGCCGAGACCTGATGGGCGTTGTTGAACAGGCGTATTTTACGTACCT

GGGTCCGCTCCTTGCGCACCCTGAAATCCAGGCTGAACGCGGTGGCGTAG

TGCTCATGCACCGTGGTGAGGATTTCCTGGTCGGCGCGCACCTTGATCCC

GCCATAGATCATCGCCAGCATCCTCCTA

>PCR product of Unigene pair 8 (lower band)

GTGGGGTGACCAAGAAGAGAACA

AACATTTTTTTTGTTAATTGCAACCACGGTTACCTTAAAATAGACACAAAATTGTAACAG

TTCAATTTTACATATGAATAAGAATTCTGAATTTAGGGTCAAAGTGATTATAATGATGCA

GAAAAGGCAATAATACAACCTCGAATTTCCACTTTAATACAAGCGAAAAGTTTTAAAAGG

TAAACCGTAGTTCACATAACAACTCAACATTAAGCTTACTGGCCATTAGGAAAGCTTGTC

TTACAGTAAGATTCCATGGAATACACCATAGCTAAGAATGACATAGATCCATCTAGAAAG

ATGAATATAAGGCCAAAAATTCAACTTTTGTGGATCCAAAAGGAAGTAACAGACTAACAG

GTTTTCACCAATGCAAATTCTAAGGCCTAAACTTAAGAAACATGACTAGCACTACTTATT

CTTGCATTGATCCACGTAACAAATAACTAATAAGCATGAAATGCTACACTGTATGTAAGA

ATGTACAAGAAAATGTTTAAATTCTTTATCCAAAGCACAAGCATAAGTCATTTTTATTGA

CACGACACAACAATGCAACAAAGTTATATCAACAGATATGTAAAGAGCTCAACCTCATCG

CCAGCATCCTCCTAAATCGGATCCCCGGGTACCGAGCTCGAATTCACTGGCCGTCGTTTT

ACAACGTCGTGACTGGGAAAACCCTGGCGTTACCCAACTTAATCGCCTTGCAGCACATCC

CCCTTTCGCCAGCTGGCGTAATAGCGAAGAGGCC

>PCR product of Unigene pair 11 (upper band)

CGTCCTCCTCCTTTATCTTCTGTG

ATAGAGTTAACAATGTTGTTCAGAATTGTTGCAGCAAAGCAACCTAGACC

TCCTGCGACCGCTGCATAGGCAGATCCTATGCTTCTCATGGCATCAGGTG

CCTCTTCATATAAGAATTCCAGCAACCCCACAATGCAAAAGACTTCAGCA

ATGCCAATGAGGCAGTACTGGATTAGCAGCTAGAATGCACTGAGATCTGG

CATCTGGGTGAGAAAGCTAGCCTCATACCCGTGGCTAATAGCATAATTTC

TTCTGAACCTCTCAAAAACCCCAGCCCAGGCAACAGAAAGGATAGAGATT

CCCATGCCAATGCCTACCCTTTGAAGCTGAGAAGCTCCATGAGGATGTCC

CGTGATGCGTCTTGATACCGGGACAAATATGTAGTAATAAAGGGAGAGAA

CAAGAAATATGCTTAGGGCAGGAAAAACAGGCATGCAGGTAACTGGGACT

TTGAGATGACCTATGTGGGTATTCATCGTGTATGCTTGTTGGACAGACAT

AGTCAGGAACTCTGTTAAGATTAGACTGAGCATAATAGTGCTTGCCGGAA

TAGGCAAGAGTTTTATCAGGATTTTGACTTCCTCCACTTGAGTAACTGTG

CAAAGCCTCCAAGGGCTTTGATCTGGGCCATCTTCTGGCAACTTTAGTGC

TGCTTTGTCCAAGCATCTACAGAAAGAGTAAGGATTATTATTATTATTAG

GAATTCATGAGTAACCCTTATCAATGTGATATGCAAGCACTGATTATAAT

TAGAATTTTTGTGATTTTAGTAACGGTTGGGTTAAACATCCTAGGTAATA

TCCAAATGCCCCTTTTTCCCCCCAAAATCTCTTATCTAGGAACAATAATC

TGTGAGTTGTGCATCTGGGTGGACAACAAACATCATGCGCCTGTATTTAA

AATAGAAAATTCATTCTATGCATTTAAAATAGAACATTCATATTGAGCAT

TTAGAACGTTTGTGTTTTATATTTAGACCACTTAATTTAATAAAATAAGT

GTTCAAAAATAAAACATGAATGTGCCAAATGAATAGAATAAATATTCAAA

ATGCATGATATGAACGTTCTATTCTAAGTGTACGTGCATGGTGATCACCA

TCCAAGCTCTCGGGTGCACGAAGAGATTTTCAAGTATTGTTTGAGGATCC

AAGGGTCTAATTTAGAAAACCCTTTGATCTAGCTTTTGGATCCAAGGGTC

TAATTTGGTGAAGTAATCCACTTAAAAAGGGTTGAACTTGGATGTTTACC

TGAAGTCATTGGTGTGAGCAATTTTCGCACTACCTTTAATAGCAGATTTT

TTCCCTGTAAGTTCATATAGGCCAATGATCTCACTGCTGGAAAAGGAAGC

ATTTCTTTTTCTGAAGGCTGCTACTAGAACTTGGGCAACACGCGTCAAAG

GACTGCCCCCTGGCAACCTGTGGCGGTAAGTGGAAGTG

>PCR product of Unigene pair 11（lower band）

GTGTTGCCCAAGTTCTAGTAGCAGCCTTCAGAAAAAGAAATGCTTCTTTT

TCCAGCAGCGAGATCATTGGCCTATATGAACTTACAGGGAAAAAATCTGC

TATTAAAGGTAGTGCGAAAATTGCTCACACCAATGACTTCAGATGCTTGG

ACAAAGCAGCACTAAAGTTGCCAGAAGATGGCCCAGATCAAAGCCCTTGG

AGGCTTTGCACAGTTACTCAAGTGGAGGAAGTCAAAATCCTGATAAAACT

CTTGCCTATTCCGGCAAGCACTATTATGCTCAGTCTAATCTTAACAGAGT

TCCTGACTCTGTCTGTCCAACAAGCATACACCATGAATACCCACATAGGT

CATCTCAAAGTCCCAGTTACCTGCATGCCTGTTTTTCCTGCCCTAAGCAT

ATTTCTTGTTCTCTCCCTTTATTACTACATATTTGTCCCGGTATCAAGAC

GCATCACGGGACATCCTCATGGAGCTTCTCAGCTTCAAAGGGTAGGAATT

GGCATGGGAATCTCTATCCTTTCTGTTGCCTGGGCTGGGGTTTTTGAGAG

GTTCAGAAGAAATTATGCTATTAGCCACGGGTATGAGGCTAGCTTTCTCA

CCCAGATGCCAGATCTCAGTGCATTCTGGCTGCTAATCCAGTACTGCCTC

ATTGGCATTGCTGAAGTCTTTTGCATTGTGGGGTTGCTGGAATTCTTATA

TGAAGAGGCACCTGATGCCATGAGAAGCATAGGATCTGCCTATGCAGCGG

TCGCAGGAGGTCTAGGTTGCTTTGCTGCAACAATTCT

>PCR product of Unigene pair 14

AAGACAGGGGCAACTCCGTTCTCTGCATGGAGGATTTCCTTCTTCATCCC

CGGTCTTATGCAAATGTTCTCAGCATTTGCAGTCATGCTTTTCGGCCAAG

ATACACCTGATGGAAATTTCCACCAGCTGCAGAAGTCTGGTACTATGCAC

AAGGACAATGCTTCCAAGATCTTTTACCATGGAATCAGCAACTATAGAAG

TTGGATCACTGCTCTAGGCTTCGGTCTTGCTTTGGGAGTGGAGCTCGCAG

TAGACAATATCGTTGCCTACTATTTCTATGATCGGTTTAACCTGAACCTC

TACGTTTCAGGAATGGTTGCAGCTAGCTTTGGGTTGGTAAATCTTTTCTC

TAGGCCTATTGGTGGTGGGATGTCAGATTATGCGGCAAAGAAGTTTGGAA

TGCCCGGAAGAATATGGACCTGGTGGGTTATTGAGTCCCTAAGTGGTGTG

ATGTGCATTGTTCTCGGACTTCTCGACCACTTGACCGCATCAATTATAGT

ATTGGTTATCTTCTCTGTGTTTGTACAAGCAGCTGAAGGACTCACATTTG

GGGTTGTTCCTTTTGTCTCCAGAAGATCTATGGGAGTGGTCTCCGGCATG

ACAGGAGCAGGTGGAGATGTAGGCGCAGTCATACTCCAGCTGATTTTCTT

CAGGGGATCTCAATTCTCAACGGAACAAGGAATAACATACATGGGTATTA

CGATCGCAGTATGCAGCTTAGGCATATTGTCCATATACTTCCCACAGTGG

GGAGGTGCATTTTGTGGTCCTAAACCATGTTTCAATGAAGAAGATTATTA

CTTAAGGGAATGGGACGCAAAGGAACAGGAACAAGGTCTTCATCTGTCAA

GCATGAAATTTGCTGAAGGCAGT

**Assembly sequences (5)**

>Assembly 3 (Unigene68619_All + PCR product + Unigene91547_All)

GTTATCGAAAGATACAATGATTCCTAAATCCTACATAAGCATTTTTAGAGTCCTTTAAAT

AGTGTCCCATCAGCCATCGTATTCCCCAAATTAACGTGTTTTTACTCGAACAAACAAACA

AACAAACAAACTTCTCTCAAAAAATCCATTACTATAATCTCCCACCTTCTTCAATTCTTC

TCTACCAATCACCTTCATCTTATTTCTTACTACCTCCATTTCCAATCAATATTATGGAAG

TAGAAAATCAAAGTGACAAATGGGAAGGCTACGTCGATTGGAAAGGCCGCCCTGCTCTCC

GCCACCGCCACGGTGGCTTTCTTGCTGCTTCCTTTGTTCTTGGGGTGGAAGTGATGGAAA

ATATGGCGTTTTTAGCAAATGCAAGCAATTTGGTGATGTATGTATCAAAAGACATGCATT

TTACACCGTCAAAATCATCAAACACCGTTACCAATTTCATGGGCACTGCTTTTCTCCTTG

CTTTGCTCGGTGGTTTCCTCTCTGATGCCTTTTTCACCACCTTTCAAGTCTACCTTACTA

GTGCTGCCCTTGAGTTTTTGGGACTAGTATTGTTGACGATTCAAGCATACAAACCGTCCC

TACAACCACCAAAATGCAACCCAATGGACACCACAACAGGAGGAGCGTGCAGGGGACTTG

ATACTTCTGAAGCAGCAATGTTGTACATAGGCCTATACCTAGTGGCAGCCGGTGTTGGAG

GGATAAAAGGGTCATTACCAACTCATGGCGCGGAACAATTTGATGAGAGCACCCAACAAG

GGAGGATAAAAAGATCTACCTTCTTCAATTACTTTGTGTTCTGCCTATCGGCTGGTGGTC

TCATTGCAACCACGTTTGTGGTCTGGGTCGAAGACAACAAGGGTTGGAAATGGGGCTTCC

TCATCTCCACCACGACTCTATTGTTATCTGTTCCTGTCTTCCTTTGTGGCTCC

>Assembly 4 (Unigene60049_All + PCR product + Unigene54473_All)

TCCACGTATGAACTCTTTAAGGAATAATAATAATAATAATAATAATGGAGAATCTGGAAT

TACTGATCAAACTACTGTTGATTGGAGAGGCAGACCTTCCAATCCTACTAAGCATGGTGG

CATGAGAGCTGCTCTCTTTGTTCTTGGGTTACAAGGATTTGAGATAATGGGAATAGCAGC

AGTGGGAAACAACCTGATAACGTACGTGATAAATGAAATGCATTATCCGTTGTCAAAGGC

AGCGAATATCGTCACCAATTTTATTGGCACCATTTTCCTCCTCTCCCTCCTTGGTGGTTA

CCTCTCTGATTCTTTTCTTGGCAGCTTTTGGACCATGCTCATCTTTGCCTTCGTCGAACT

TTCTGGATTCATCCTACTATCAGTGCAAGCACACCTACCACAACTAAAACCACCAAAATG

CAACATGCTAGAAGCAGATAAAGTATGTGAGGAAGCAAAAGGGATAAAAGCAGTCATATT

CTTTGCAGCACTTTACTTAGTAGCCTTAGGAAGTGGTTGTGTAAAACCTAACATGATAGC

TCATGGTGGTGATCAATTCAACTCTAACCAATCTAAACAACTCTCCACCTACTTCAACGC

CGCCTACTTCGCCTTCTCCGTTGGTGAGCTCATCGCCCTCACCGTCCTCGTTTGGGTCCA

AACCCATTCGGGTATGGATATCGGGTTTGGTATCTCTGCTATTGTCATGGCTATGGGCTT

GATTTGCTTGGTTTCTGGTACTCTTTTTTACAAGAACAAGAGGCCTCGTGGTAGCATTTT

CACTCCTATTGCTCAGGTATTTGTGGCCGCATTTTTAAACAGAAAGAAAGTAAGCCCGGA

CGTGAAGCTCCTTCATGGAAGTTACAATGCATCACACAATAATCTCATACACACTGAGAG

ATTTAGGTGTTTGGACAAGGCATGCATAAAAACAGAAGGAGAAGAACAAGGACCATGGAG

AGTATGTTGCACAGTAACACAAGTAGAACAAGTAAAACTCCTAATATCAATCCTCCCAAT

ATTTGGTTGCACAATAGTATTCAACACTATCTTAGCCCAACTCCAAACATTCTCAGTCCA

ACAAGGCAGTCAAATGAACACACAACTCACTTCTTCATTAAGCATACCCCCAGCCTCACT

CCAATCCATCCCTTACCTCCTCCTCCTCTTCCTCGTACCTCTCTACGACAAACTCTTCGT

CCCTTTCATCCGCCGCTTCACGGGCCACCCCTCGGGCCTCTCCCCTCTCCAACGAATCGG

GTCGGGCCTCTTCCTCGCCACCTTCTCCATGATCTCCGCCGCCCTCATTGAAAACCACCG

TAGAAACCACCACCACCAACCGCTCTCTATCTTCTACATCACCCCACAATTCCTCATATT

TGGACTATCCGAAATGATGACTGCCGTTGGACTTATTGAATTTTTTTACAAACAGTCACT

TAAAGGAGGGTCATTACAGTCTTTTCTTACGGCGATTACTTACTGCTCTTACTCTTTTGG

GTTTTACCTTAGCTCTGTGTTGGTTACCCTTATTAATAAACTCACTTCTTCTTCTAAGAC

TAATTATCAAGGGTGGTTAAGTGATAATGACCTTAATAATGATCGGTTAGACTTGTTTTA

TTGGTTATTAGCTGGGCTTAGCTTCTTAAACTTCCTTAATTACCTATTTTGGTCTCGTTG

GTACTCTTTTAATCCTTCTCTTTTGCTCTCTTCTCAACCACATGTTTCTCCTCATGATGA

CCTCACCCCTCCTCCTACCAATAATACACATTCATTAATTTAATTAGATTATAATTAAAT

TAAGGAAAATTTGTTAGAAACCACCTTATGAATTAAATTTTTCGTCAAAAACCACCTTTT

AAATTATTATTTTGTTTGTAAGAAGCCACCTTTTTTGGATTTTTTTTTTGGTGAGGAACC

ACCTTTGGTTGAATTTCGGGTTTTGATTTTGGGTTATTTTCGGCGTGGTGGTTTAGGGGA

GGAAAAGCGATGGAGACGCTTGGGGAGGTACGAAGGGAGTTGCCGAAATCATGGAGGGAG

GTTTGATTTAAACACCGAAACTAAGCTAAAGGTGATTCTTTACAAAAAAAAAAAAATTAA

AAAAAGGTGGCTTCTTACAAAAAAAAAAAAAATTTAAGGTGGTTTTTGACACAAAACAAA

AATGTTTTATAAGGTAGTTTTTAACAAATTTTCCTTAAATTAATGTATAATTTCATGATA

TTTCTTTCTTATTATGTACTAATTAATTAATTAG

>Assembly 7 (Unigene49607_All + PCR product + Unigene30293_All)

CTGTTTTTGACAAGAGTGGTTGGGCAAACCAACGCTAATGCAGCAAATAATGTTAGTACA

TGGATTGGTACTGTTTATCTCTTCTCTCTTGTTGGTGCCTTCCTTAGTGATTCCTATTGG

GGAAGATTCAAGACTTGCGCTATTTTTCAGGCTATCTTTGTCCTTGGATTGGCATTATTA

TCACTATGCACATACTTATTTTTACTTAACCCAAGAGGGTGTGGAGATAAACGTACACCA

TGTAACAAGCCCTCAAACCTACATGTATCACTATTTTATGTATCACTCTACATGGTTGCC

CTTGGCAACGGTGGTTATCAACCCAATATTGCCACATTTGGGGCAGACCAATTTGATGCC

GAATTTCCAAAGGAAAGGACCTCCAAAATATCCTTCTTTAGTTACTTTTACTTGGCCTTT

AACTTAGGCTCTCTATTTTCCAACACCATATTTGCTTACTATGAAGATGTTGGTAAGTGG

GTGATTGGATTTTCGGCGTCTGCCGCTTGTGCTCTGGTGGCTTTGTGGTTGTTCTTTGGC

GGCACGCCTTGGTATAGGCACTTTAAATCACAAGGCAATCCGATTTCGAGGATTTCCAAG

GTGTTTGGAGCTTCCTTAAAGAAATGGAAGGCTCATGTTCCTCCTGAAGTTGGCTTGTAT

GAATTGGATGCCAAAGCTTATTCCAAGACTGGGTGCCGTAAGATCTCCCATACACAAGGT

TTCAAGTTCTTGGATAAAGCCGCAGTGATCACACCAGCAGAACACGTTGGACTGAACAAA

AAGATGTATGAACAAAAGTACTTGTGCACAGTTACACAAGTTGAAGAAGTGAAATGCATC

TTACGACTTTTACCAATATGGGTTTGCACTATTATGTATTCTGTAGTTTACACTCAAATG

GCATCCCTATTTGTGGTTCAAGGTGCTGAAATGGACGCGACCTTAGGACCCTACCACATT

CCCCCAGCTAGCATGTCAAGTTTCGATATCCTTAGCGTTGCAGCCTTCATATTCATCCAT

AGTCACATCGTGAACCCTTTTGTGATGAGATGGAGGAAGAAAGGTTTAAACGAGCTTGAA

AGGATGGGGATCGGCCTAATCTTTGCAATTATGGCCATGATCGCGGCAGGAGTTGTGGAA

ATCTACAGGCTGAAATACGCGACAGGGAATTGTAAGGGATGTAGCGGTGCAGCATACGCC

AGCTCACTGAGCATCTTATGGCAAATTCCGCAGTTTGTGCTGATTGGTGCATCTGAAGTT

TACATGTATGTGTCGCAGTTAGAGTTCTTTAATGGACAAGCGCCCGAAGGAGTGAGGAGT

TTTGGGAGCGCACTTTATACAACATCAATGTCTCTAGGGAATTATGCAAGTATCATAATA

GTGACCATCGTAATGAAGATAACCACTAGGGATAACATGCAAGGATGGATACCACAAGAC

CTAAATGAGGGTCGATTGGACAAGTTCTACTTCTTATTGGCTTTTCTTACTATGCTTGAT

TTTGGGGTGTTCTTGGTCTATGCAAGGGCTTATAAATATGTAGAAACTAATAATAGTGAT

TATAAGGACGAATGTATTGATGTCTAAGTGAACTTGGTATTTACAATTGCAAATTTAATG

ATTATGCGCAAGTTTCGTAATAAACAATATTTTACATGTTTAGTGAAACACG

>Assembly 11 [Unigene34465_Al + PCR product (lower band) + Unigene61016_All]

GAATTATTAATCAAAGTTGAAATGAAAACAGCATAAATGACAAGAATAAATATTTTGAGA

AATAAGGGGGTACATTCTGTGCCAACCATCAACTAACAAACCTTCATATCTCTGCACTAA

AAGAAACAGACTTTCATATTTCAACATAAATGAGATTCATATTGCATAACAAAAAAAAAA

CCCATACATCATCCTCAAGACCTCAAATCCATTGTAGTTGCACCACATGAATAAAATGTA

TATAGAAGCAGAAATTTGAGTCTGAAATAATGATGCTAATCAAGTAAATAAACAAGGTAA

TAAGAAGGAAGTGAGAATATTCACACAGTGTGTGTGTGAGGCAGAAATGGGTAGTGCAGA

AATCCGGTCACCTCAAATTCAAGTTCTTGGAACACCATCTACTCCTTCCAAAGGCATGGA

CATGTCTAAGAGAAAGAAACTGGGAATCTATTTCATGGAGTCAGACGATAGACGAGGAGC

ACTTGGTGGAGGTTATACTGTAGGGAGTACACCAGTTAACATTCACAGGAAGCCATTGTC

TGAAGATGATCTCTCTAAGACCGGTGGTTGGGTTGCCGCCTTTTTCATCTTTGGAAACGA

GATGGCTGAGAGAATGGCTTACTTTGGTCTATCAGTGAACATGGTGGCTTTTATGTTCTA

TGTTATGCATCGGCCCTTCACTAGTTCGGCCAATGCTGTCAATAATTTCCTTGGAATATC

ACAGATTTCATCCGTGCTTGGTGGCTTTCTAGCTGATGCCTATCTTGGTAGATATTGGAC

TATTGCAATTTTCACAACCATCTATCTTATGGGGTTGACAGGGATAACACTATGTGCAAC

AATGAAGTCGTTTGTGCCGAACCAAGATTGCAGTCAGCTAGCAGTTCTTGTGGGGAATTG

TGAAGCAGCAAAACCATGGCAGATGCTTTACCTGAACACAATTCTCTATATAACCGGATT

TGGAGCAGCAGGCATAAGGCCGTGTGTCTCCTCTTTTGGAGCTGATCAATTTGACGAAAG

AAGTCCAGATTACAAGGCTCACTTGGACAAGTTCTTCAATGTGTTCTATCTGTCTGTCAC

AGTTGGTGCTATTATAGCATTCACAGCAATTGTTTATATCCAGATGCAGCATGGCTGGGG

ATCTGCATTCGGATCGTTAGCATTGGGGATGGGCATATCAAATGCGCTCTTCTTCATTGG

CACTTCCACTTACCGCCACAGGTTGCCAGGAGGCAGTCCTTTGACGCGTGTTGCCCAAGT

TCTAGTAGCAGCCTTCAGAAAAAGAAATGCTTCTTTTTCCAGCAGCGAGATCATTGGCCT

ATATGAACTTACAGGGAAAAAATCTGCTATTAAAGGTAGTGCGAAAATTGCTCACACCAA

TGACTTCAGATGCTTGGACAAAGCAGCACTAAAGTTGCCAGAAGATGGCCCAGATCAAAG

CCCTTGGAGGCTTTGCACAGTTACTCAAGTGGAGGAAGTCAAAATCCTGATAAAACTCTT

GCCTATTCCGGCAAGCACTATTATGCTCAGTCTAATCTTAACAGAGTTCCTGACTCTGTC

TGTCCAACAAGCATACACCATGAATACCCACATAGGTCATCTCAAAGTCCCAGTTACCTG

CATGCCTGTTTTTCCTGCCCTAAGCATATTTCTTGTTCTCTCCCTTTATTACTACATATT

TGTCCCGGTATCAAGACGCATCACGGGACATCCTCATGGAGCTTCTCAGCTTCAAAGGGT

AGGAATTGGCATGGGAATCTCTATCCTTTCTGTTGCCTGGGCTGGGGTTTTTGAGAGGTT

CAGAAGAAATTATGCTATTAGCCACGGGTATGAGGCTAGCTTTCTCACCCAGATGCCAGA

TCTCAGTGCATTCTGGCTGCTAATCCAGTACTGCCTCATTGGCATTGCTGAAGTCTTTTG

CATTGTGGGGTTGCTGGAATTCTTATATGAAGAGGCACCTGATGCCATGAGAAGCATAGG

ATCTGCCTATGCAGCGGTCGCAGGAGGTCTAGGTTGCTTTGCTGCAACAATTCTGAACAA

CATTGTTAACTCTATCACAGAAGATAAAGGAGGAGGACGCCCTTCTTGGTTATCCCAGAA

TATCAATAATGGCAGATTTGATTACTTATACTGGCTGCTTACAGGACTCAGTATAGTCAA

TTTCTGTGGTTTCCTTTATGCAGCCAGGAGATACAAATATAGATCAGTCTTCCAAGTGGT

ACAATGATAGATGAAAGAAGCACAAGCCTTCAACACAACCTTAAATCTAAGAATATAATT

GAGTCCTTCCAAGTCATTCTTCCAAGTCCTTCCATAGCTCCCATGCAATCTTGAAAACAG

AAACTTTTTAAAACTCTTTGTTCAAGATGAACAATAATCATGAAATTACCAAATTGAGTT

GTTGCAATTGTACTCTGATGATTGATGAGATTAGTTATAAAAAAAAAATCTACTTTACAG

CTCTTGTTATGCTGTCAAGATATTAATATTTGCACCAA

>Assembly 14 (Unigene34113_All + PCR product + Unigene5588_All)

CCCCCCCACAAAAAAAAAAAAAAACAAAACTTGCCAAATACAGGCAAAACATAAAGAGAG

ATGGCATCAGTAAATGAAATGAGCGAGCCTGAACCCAAAAAATTTGATCTTCCAGTTGAT

AAAGAGAACAAGGCCATAATCCTGCCAATATTTTCAGTAGCAAAACCTCACATGAGGGCA

TTTCATCTATGTTGGATGCAATTCTTTACCTGTTTTCTGTCCACTTTTGCAGCTCCACCC

CTCCTTCCCATCATTCGTGACAACCTCAACCTCACAGCCCGGGACATCGGCAACGCAGGG

GTCGGGTCTGTGTCGGGTGCAATCTTTGCTTGTGTTGCCATGGGTGCTGCCTGTGACATA

GTCGGGCCACGACTAGCCTCTGCCACCCTCGTCCTCCTCACTGCACCTGCAATCTACCTG

TCTTCTATGGCTGATTCAGCCATGGAATTCTTTCTGATGCGGTTTTTTATAGGATTCGGG

TTAGCCACATTAGTCCCAACTCAATACTGGATGAGCTCCATGTTTTCCCCAAGAGTACTG

GGGCAAGCAAACGGGCAAACGGCCGGGTGGGGAAATCTAGGAGGAGGAGTTGCCCAGTTC

CTCATGCCCCTTGTCTACAGCCTAATCCTTAAGACAGGGGCAACTCCGTTCTCTGCATGG

AGGATTTCCTTCTTCATCCCCGGTCTTATGCAAATGTTCTCAGCATTTGCAGTCATGCTT

TTCGGCCAAGATACACCTGATGGAAATTTCCACCAGCTGCAGAAGTCTGGTACTATGCAC

AAGGACAATGCTTCCAAGATCTTTTACCATGGAATCAGCAACTATAGAAGTTGGATCACT

GCTCTAGGCTTCGGTCTTGCTTTGGGAGTGGAGCTCGCAGTAGACAATATCGTTGCCTAC

TATTTCTATGATCGGTTTAACCTGAACCTCTACGTTTCAGGAATGGTTGCAGCTAGCTTT

GGGTTGGTAAATCTTTTCTCTAGGCCTATTGGTGGTGGGATGTCAGATTATGCGGCAAAG

AAGTTTGGAATGCCCGGAAGAATATGGACCTGGTGGGTTATTGAGTCCCTAAGTGGTGTG

ATGTGCATTGTTCTCGGACTTCTCGACCACTTGACCGCATCAATTATAGTATTGGTTATC

TTCTCTGTGTTTGTACAAGCAGCTGAAGGACTCACATTTGGGGTTGTTCCTTTTGTCTCC

AGAAGATCTATGGGAGTGGTCTCCGGCATGACAGGAGCAGGTGGAGATGTAGGCGCAGTC

ATACTCCAGCTGATTTTCTTCAGGGGATCTCAATTCTCAACGGAACAAGGAATAACATAC

ATGGGTATTACGATCGCAGTATGCAGCTTAGGCATATTGTCCATATACTTCCCACAGTGG

GGAGGTGCATTTTGTGGTCCTAAACCATGTTTCAATGAAGAAGATTATTACTTAAGGGAA

TGGGACGCAAAGGAACAGGAACAAGGTCTTCATCTGTCAAGCATGAAATTTGCTGAAGGC

AGTAGAAACGAGAGAAGCACCAAGAAAATGTCAGCAACAACAGAACAAGAGATGAGCACA

ACCAATCAAGTCTAAGCTAATTCAGTACTTACAGCAGAGAGAACACCGGATCATGTCCAA

TTGCTGCACTGTAATATGTTTTATACTATATGAGCTTCTCAAAAGAAATGAACTACGGAG

AAAATTTTCCTACTGCAGCTGCTTCCAACAGTTTCCTTTTCCTTTCTGCTTTTTTGCATA

TATGTCATGCTTTACAAGTTTTCGATAACAAGAGTACAACTGTACAAGAATTGTATCTAT

TTTTTCCCATCCTTGATTTGATATTTAAGTTTTTTAATTATACTCCCAAATGGAGTCCTT

GTAAGCAAAGCCTTCAATTGGATCTTCACATCCAAGATGTATGCCAATAGGCCAGTGTAC

AAATAAAATCCATGAGTTGAACAAAAGATAATGGAATTGCAAAGCACAAATACAGACTGT

TTACATCACGTCTCATCATTAATAAACACTAAAATCTTTTATTGGCATCT
