# Supplementary material for: A method for the further assembly of targeted unigenes in a transcriptome after assembly by Trinity
Source: Front Plant Sci. 2015 Oct 14;6:843. doi: 10.3389/fpls.2015.00843 (PMC4604318; doi:10.3389/fpls.2015.00843)
Supplement: Data Sheet 2 — PHT sequences of Salicornia europaea. The doc file contains the sequences of 12 unigenes pairs, 8 sequences of PCR products, and 3 assembled sequences. [file DataSheet2.DOC]

**Sequences of 12 unigene pairs**

>Unigene91380_All + nr

GTGAAGTTCAGTGAAGCGTTAAACGACGTAGTTGTGGAGGAGAAATGGTGGGAGCAGTTT

CCTAAACGATGGATTATAGTCCTTCTGTGTTTCTCTGCTTTTCTTCTCTGTAACATGGAC

AGGGTTGTGTAACTTTAAACAGATGCTGACTGGCAGGTGAATATGAGCATTGCAATACTT

CCAATGTCAGCCGAGTATGGGTGGAATCTAACTACTGTTGGTTTGATCCAGTCTTCTTTC

TTCTGGGGCTATCTTCTTACACAGATAGCAGGGGGTATATGGGCAGACACTGTAGGGGGC

G

>Unigene105912_All + nr

CACGGCCATCTTTGACTCGTTCGCCCACGGCGCCAACGACGTGGCCAACTCGATCGCCCC

CTTTGCCGCCATCTATGCCATCTACACCACCGGCGAGATCCCCTCCAAGAGCGCCGTGCC

CGTGTGGATCCTCGCCCTCGGCGGCGCCGGCATCGTCGTCGGCCTCGTGCTCTACGGCTA

CCGCATCATGCAGGCCATTGGCATGCAGCTCACCAAGATCACCCCCTCCAGGGGCTTTGC

CATTGAGCTCGGCACCGCCTTCACCGCCGTCACCTTCTCCATCCTCGCCGTCCCCATCTC

CACC

>Unigene5546_All + nr

AACTAAAAAGTCATGTTAACTTTGTAGGTATTGTATGCTATATAAGGCCAACACTGAGTA

AAAACAAAATAGACTAAAGAGAAGAGACAACTCTTGCTTTCATGGCGAAAGGACAACAAT

TGGAAGTGCTTAATGCACTTGATGTGGCAAAAACACAATGGTACCATTTCACAACTATTG

TGATTGCCGGTATGGGCTTCTTCACAGATGCCTACGATTTATTTTGCATTTCCTTATTGA

CAAAACTTTTGGGGCGTCTGTACTACACTGAACCTGGTGCAACAAGGCCAGGGGCTTTAC

CTATTAGTGTATCAGCTGCTGTGAGCGGTGTTGCTCTGTGCGGCACTCTAGCTGGTCAAC

TCTTCTTTGGTTGGCTTGGAGACAAAATGGGTAGGAAAAAGGTGTATGGTATCACCTTAG

TTTTGATGGTTTTTACTTCTTTTGCCTCTGGTCTTTCTTTTGGAAGCAGTGCAAAGGCGG

TTATGACCACCCTTTGCTTTTTTCGCTTCTGGCTTGGGTTTGGTGTTGGAGGTGACTATC

CCCTTTCTGCCACCATCATGTCTGAATATGCT

>Unigene11489_All + nr

TTGGCGTATGCAGATGCCTGAGACCGCTAGGTACACTGCCCTAGTGGCAAAAAATGCAAA

ACAAGCAGCTGCCGACATGTCCAAAGTCCTCAAGGTTCAGATAGACGCCGATGAGGAGAA

TTATGCGAAAATTTCAAAAAGTGGCAAGAACGATTTCGGGCTATTCAGTCGTGAATTTGT

TCGTCGGTACGGAGTTCGCCTTTTAGGAACCACAAGCACGTGGTTCCTACTAGACGTTGC

TTTTTATAGTCAAAACCTTTTCCAAAAGGACCTT

>Unigene5546_All + nr

AACTAAAAAGTCATGTTAACTTTGTAGGTATTGTATGCTATATAAGGCCAACACTGAGTA

AAAACAAAATAGACTAAAGAGAAGAGACAACTCTTGCTTTCATGGCGAAAGGACAACAAT

TGGAAGTGCTTAATGCACTTGATGTGGCAAAAACACAATGGTACCATTTCACAACTATTG

TGATTGCCGGTATGGGCTTCTTCACAGATGCCTACGATTTATTTTGCATTTCCTTATTGA

CAAAACTTTTGGGGCGTCTGTACTACACTGAACCTGGTGCAACAAGGCCAGGGGCTTTAC

CTATTAGTGTATCAGCTGCTGTGAGCGGTGTTGCTCTGTGCGGCACTCTAGCTGGTCAAC

TCTTCTTTGGTTGGCTTGGAGACAAAATGGGTAGGAAAAAGGTGTATGGTATCACCTTAG

TTTTGATGGTTTTTACTTCTTTTGCCTCTGGTCTTTCTTTTGGAAGCAGTGCAAAGGCGG

TTATGACCACCCTTTGCTTTTTTCGCTTCTGGCTTGGGTTTGGTGTTGGAGGTGACTATC

CCCTTTCTGCCACCATCATGTCTGAATATGCT

>Unigene47851_All + nr

ATAGCGGCTGTTTTTGCAATGCAAGGCATGGGTATCTTAGCTGGTGGGATAGTGGCCTTA

ATAGTTTCGGCTGCCTTTGAAAGGAAGTTTAAACCTCCTCCCTTCAAGGATGATCCGATT

GGCTCTATCCCACCGGAATCAGATTACATTTGGCGAATTGTCTTAATGTTTGGGGTTGTC

CCTGCTGCCTTGACCTACTATTGGCGTATGCAGATGCCTGAGACAGCTAGGTATACTGCT

TTAGTGGCCAACAATCCTAAACAAGCTGCAGAAGACATGTCAAGGGTACTCAATTTTCCT

TTAGATCCTGAGGAAGAGAGGGTTAAGAACATCACAAAAAGCGAAGGGAACAAGTTTGGG

TTGTTCTCAAGAGAGTTTGTCCGACGGTATGGTATTCGTCTCCTAGGAACCACTAGCTCT

TGGTTCTTACTTGATGTTGCATTCTATAGTCAAAACCTTTTTCAAAAGGATCTTTTATTC

TCAGTTGGGTGGATCCCACCCGCCCGTGAAATGAATGCTATCAAGGAGGTGTATATGATA

TCCAGGTTTTCTACTTTGATTGCTCTTTGTGGAACTGTTCCGGGATACTGGTTTAGTGTA

GCCTTCATAGACGTCATCGGACGTTGGTGGATCCAAATGATGGGATTCTTTTTTATGACA

CTTTTTATGTTCGCCATTGCCTTTCCTTATGAATATTGGAAGACCAAGGATCATCGTATT

GGGTTTATCATAATGTATGGGCTTACATTCTTCTTTGCCAACTTTGGTCCTAATGCAACC

ACCTTCGTTGTGCCTGCAGAGATCTTTCCGGCTAGGCTTAGGTCCACTTGCCATGGCATT

TCTGCAGCATCAGGAAAAGCAGGGGCTATAGTAGGAGCCTTTGGCTTTCTTTACGCCTCT

CAAGAGACAGACCCCAATAAGAGAAGTCATAATTATCCAGCTGGTATCGGCAAAAGAGGC

TCACTTATTGTTCTTGGTATCATCAACTTTCTTGGTATGCTTTGCACATTTCTCATACCT

GAGCCTATGGGTAAGTCTCTAGAAGAACTTGCCGGTGAGGTTGATATGGAAGAGGTTGAA

TATGAGCGTATTACTGTTTGATTATGTTTTAATTTACATAATAAACCTATAAATCCATAA

ATAGTAAACTTTTTAAAAAAATATATATATGCTTGACTTTCTTTGCTCTCTAGAATATAC

TCCCTCCGATTTTTTTTAGATGTAACACTTCTCTTTTCGTACTTATCGAGACACAGCTTG

GACTCATAATAATATCTTTAAATATATAAGTGCAAAAATTATAAAATTTATATATTTGAA

AAGTGTATTCCGAGGTAGATCCAACAAGATATCACTCGATTA

>Unigene16022_All - nr

AATGAACTTAATGAAAAAGGAGAAGCATTGTACCAGCTTGAAAGCCTCAATCAAACCCTT

GTATTGAATGAGTGTTCAAGCAATGAAGAGCTGAAAGATGCTAGAATGGAAGCCATTAAA

TGTTTCTTAACTATGGATAGCAATGAAACAAAAATCGGAATCAAAAGAATAGGAGAGATT

GATCCACAACCTTTCCGAGATATATGTTCCAAGAAACTTTGCAGTAGAAACTGGGAGGAA

GAATCAGATGAGCTGTATTCTTTGTGGCAAGAAAACGTCAAAGATCCAAGCTGGAATCCC

TTTAAGAAAGAAATAAAGAACGGGCAAGTTAGTATGCAGGAGACTGTTGATGAAAATGAC

AAGAAGCTAGTTCAGTTAAGAGAAGAATGGGGTGAAGAACCATCCAAAGCTGTTGTTACT

GCACTGCTGGATCTAAAGAAGTACAACCCTAGTCTGAGGTCTTCTATTCCCGAACTCTGG

AACTTTGAAGAGGGAAGAAGAGCAAATTTGAAAGAAGTAATTCAGTACATGATTCATCAA

TGGGAAGTTAATAGAAACAAAGCCATTGAGCAGTCTGGTTCTATCATTGGGCTGATGGAA

GAAAAACAGAAACTACTTCAGAAATTGGCAAGTCTCAATATTCTAATGGAAGAGAAGGAC

AAGGTTCTCAAACAAAAATATGCAGCAGTTAGCAAATTGATAGCTCAGGATATGGAGAAG

GATAAACTAATTGGCCAACTATCAGTAACTATCAGCGCTTTGAAAGCTGAGAAACATACT

CTTTTTGAAAGTTGCAATGCAGAGATTGTAACGATGAAATGCATGAAGGAGCAGACTGAA

AAGTTGAAGAAGTTGCAATCTCAGGAGGAGCTGGCAAAGCAAACAGAGGAATCTAAGGAG

ACAAAGCAACATAATTGCGGGGGTGAAAAAAGTGTGACAATCAAGGAAGATTACTCCAAG

AGGCGTCTAGATTCTTTGACAGTGCAAGTGGGGCCAATTGCAGAAAACAGGAAGCACTTC

CCATTTCATAATACCAAAGAGCAAATTAGCTCAACATCAGAAAATAAAAAGTTTCAAGGT

GAAATGTTAAAGCTTTGTAAAGCTGAGCCTGGCATGCTCGGTGAATTATCACATGGGAGG

TGGGATGTACTAGAGTTATTGAAACCAACTACGCACTCAAACTCTCTACCCTCCAAAGCT

CTGGTTTCCTCTCCATCAAACAACTCATCAATAGCCTTGGAAAGATTTTTTCAGACAACT

AATGTGAAGAAAGAAGATGAAGACTAGGTTATGAAGCGAAGGACATAATGCAGTTTTTCA

GGCAGCTTGCTTGTTTGTGTCTCGTAACATAAGCTCATATTAAAGTTGATGAAAGGAAAC

CTGTGTCCTGGCAGCTTCAATATCAAGGAACAACCTATCATCCGAGGTTTTCAATTATTA

CCAAAATCGGTGTAAGAGTTCAAGTGTTTACAATGATTACGAGTGTTCCAGAAGTCTAAA

TTGGAAACGTCTGAATTTGTGCAGAATTATGCTCAAAGTTAAGTTGTTTAATTGAGTTAT

TAGGCAATACAAATCGTATGGTCCATTTCATCACACACCCAGAAAAAAAGAAAAAAGAAA

AAAACTTTGCTTTCCAAAGTTTGAAATTGAATTAATTATAAGCCTTTTGTGTTACTACTC

ATAATAATTGAGGGTAAACACAAATTATACCAATTCCCCCCTGTTTAGTTGCTGCTTCTT

TTTTTCCCTGTTTTCTTGTCATTTTGACTACAAAGTAACAATCCAGCTCCGCCACCACAC

GGAGAAAAGGTGATGATGATGATGTAAAAGGAAATTAAAAATTATAGCTTCGAGCAGGTG

ATTGATTGAGTTAATTAATCATGTTAACCGGACAATTGCAAGTGTTGAATGCGCTTGATG

TGGCAAGAACACAGTGGTACCATTTTAAGGCCATATCGATTGCTGGTATGGGCTTCTTTA

CAGACGCCTATGATTTGTTTTGTATTTCGATGGTGGCAAAGTTGCTTGGGCGATTGTACT

ACACCCGCCCTGGGGCTGAGAGCCCCGGGACTTTGCCCATCAATGTAACAGCTACCGTGA

GTGGTGTTGCGCTATTTGGAACCATGATAGGCCAACTCTTCTTCGGTTGGCTTG

>Unigene50223_All - nr

CTTTGCCAACTTTGGTCCTAATGCCACAACTTTTGTGGTGCCCGCTGAGGTTTTTCCAGC

TAGGTTGAGGTCCACCTGCCATGGAATATCCGCTGCAACTGGTAAAGCAGGAGCTATTGT

AGGGGCCTTTGGATTTGTCTATGCTTCTCAAGAGAGAGACCCCGCAAAGAGAGATCATGG

TTATCCAGCAGGTATTGGCAAAAAGAACTCCCTTATTGTTCTTGGTTGCACTAACTTTCT

CGGTATGGTCTGCACTTTATTTGTACCGGAGTCTATGGGTAAATCTCTTGAAGAATTAAC

CGGTGAGATTGACGAAATTGAGGATAACGAAACACGTGCCGTTTAATATAATTACCATCT

TACGGGTTTGAGAATGCATGTTGGCTGCCAAGCCGCTTCTTGACATACTACTATTAACTC

ATCATGTTAGTAGTTTTGTTTTATTTCCCGAAACTCATTTAAATTGTTGTATCACATGTT

TTTCTTCTTTGTATCTAATGGTACTATTTTATTCGCTTCAACATTTGTTGCGTTATCCTC

AA

>Unigene16022_All - nr

AATGAACTTAATGAAAAAGGAGAAGCATTGTACCAGCTTGAAAGCCTCAATCAAACCCTT

GTATTGAATGAGTGTTCAAGCAATGAAGAGCTGAAAGATGCTAGAATGGAAGCCATTAAA

TGTTTCTTAACTATGGATAGCAATGAAACAAAAATCGGAATCAAAAGAATAGGAGAGATT

GATCCACAACCTTTCCGAGATATATGTTCCAAGAAACTTTGCAGTAGAAACTGGGAGGAA

GAATCAGATGAGCTGTATTCTTTGTGGCAAGAAAACGTCAAAGATCCAAGCTGGAATCCC

TTTAAGAAAGAAATAAAGAACGGGCAAGTTAGTATGCAGGAGACTGTTGATGAAAATGAC

AAGAAGCTAGTTCAGTTAAGAGAAGAATGGGGTGAAGAACCATCCAAAGCTGTTGTTACT

GCACTGCTGGATCTAAAGAAGTACAACCCTAGTCTGAGGTCTTCTATTCCCGAACTCTGG

AACTTTGAAGAGGGAAGAAGAGCAAATTTGAAAGAAGTAATTCAGTACATGATTCATCAA

TGGGAAGTTAATAGAAACAAAGCCATTGAGCAGTCTGGTTCTATCATTGGGCTGATGGAA

GAAAAACAGAAACTACTTCAGAAATTGGCAAGTCTCAATATTCTAATGGAAGAGAAGGAC

AAGGTTCTCAAACAAAAATATGCAGCAGTTAGCAAATTGATAGCTCAGGATATGGAGAAG

GATAAACTAATTGGCCAACTATCAGTAACTATCAGCGCTTTGAAAGCTGAGAAACATACT

CTTTTTGAAAGTTGCAATGCAGAGATTGTAACGATGAAATGCATGAAGGAGCAGACTGAA

AAGTTGAAGAAGTTGCAATCTCAGGAGGAGCTGGCAAAGCAAACAGAGGAATCTAAGGAG

ACAAAGCAACATAATTGCGGGGGTGAAAAAAGTGTGACAATCAAGGAAGATTACTCCAAG

AGGCGTCTAGATTCTTTGACAGTGCAAGTGGGGCCAATTGCAGAAAACAGGAAGCACTTC

CCATTTCATAATACCAAAGAGCAAATTAGCTCAACATCAGAAAATAAAAAGTTTCAAGGT

GAAATGTTAAAGCTTTGTAAAGCTGAGCCTGGCATGCTCGGTGAATTATCACATGGGAGG

TGGGATGTACTAGAGTTATTGAAACCAACTACGCACTCAAACTCTCTACCCTCCAAAGCT

CTGGTTTCCTCTCCATCAAACAACTCATCAATAGCCTTGGAAAGATTTTTTCAGACAACT

AATGTGAAGAAAGAAGATGAAGACTAGGTTATGAAGCGAAGGACATAATGCAGTTTTTCA

GGCAGCTTGCTTGTTTGTGTCTCGTAACATAAGCTCATATTAAAGTTGATGAAAGGAAAC

CTGTGTCCTGGCAGCTTCAATATCAAGGAACAACCTATCATCCGAGGTTTTCAATTATTA

CCAAAATCGGTGTAAGAGTTCAAGTGTTTACAATGATTACGAGTGTTCCAGAAGTCTAAA

TTGGAAACGTCTGAATTTGTGCAGAATTATGCTCAAAGTTAAGTTGTTTAATTGAGTTAT

TAGGCAATACAAATCGTATGGTCCATTTCATCACACACCCAGAAAAAAAGAAAAAAGAAA

AAAACTTTGCTTTCCAAAGTTTGAAATTGAATTAATTATAAGCCTTTTGTGTTACTACTC

ATAATAATTGAGGGTAAACACAAATTATACCAATTCCCCCCTGTTTAGTTGCTGCTTCTT

TTTTTCCCTGTTTTCTTGTCATTTTGACTACAAAGTAACAATCCAGCTCCGCCACCACAC

GGAGAAAAGGTGATGATGATGATGTAAAAGGAAATTAAAAATTATAGCTTCGAGCAGGTG

ATTGATTGAGTTAATTAATCATGTTAACCGGACAATTGCAAGTGTTGAATGCGCTTGATG

TGGCAAGAACACAGTGGTACCATTTTAAGGCCATATCGATTGCTGGTATGGGCTTCTTTA

CAGACGCCTATGATTTGTTTTGTATTTCGATGGTGGCAAAGTTGCTTGGGCGATTGTACT

ACACCCGCCCTGGGGCTGAGAGCCCCGGGACTTTGCCCATCAATGTAACAGCTACCGTGA

GTGGTGTTGCGCTATTTGGAACCATGATAGGCCAACTCTTCTTCGGTTGGCTTG

>Unigene90558_All - nr

CTTTGCCAACTTTGGTCCTAATGCCACAACTTTTGTGGTGCCCGCTGAGGTTTTTCCAGC

TAGGTTGAGGTCCACCTGCCATGGAATATCCGCTGCAACTGGTAAAGCAGGAGCTATTGT

AGGGGCCTTTGGATTTGTCTATGCTTCTCAAGAGAGAGACCCCGCAAAGAGAGATCATGG

TTATCCAGCAGGTATTGGCAAAAAGAACTCCCTTATTGTTCTTGGGGTCACTAACTTTCT

CGGCATGGTTTGCACTTTATTTGTACCTGAGTCTATGGGTAAATCTCTTGAAGAATTAAC

CGGTGAGATTGACGAAATTGAGGATAATGAAACGCGTGCCGTATAATATAATTACCATCT

TACTGGTTTGAGAATGCATGTTGGTTGCCAAGCCACTTCTTGACATACTACTACTAACTC

ATCACGTTAGTAGTTTGGTTTTATTTCCCGAAACACATGTGATATTACAATTCAAATGAG

>Unigene11489_All + nr

TTGGCGTATGCAGATGCCTGAGACCGCTAGGTACACTGCCCTAGTGGCAAAAAATGCAAA

ACAAGCAGCTGCCGACATGTCCAAAGTCCTCAAGGTTCAGATAGACGCCGATGAGGAGAA

TTATGCGAAAATTTCAAAAAGTGGCAAGAACGATTTCGGGCTATTCAGTCGTGAATTTGT

TCGTCGGTACGGAGTTCGCCTTTTAGGAACCACAAGCACGTGGTTCCTACTAGACGTTGC

TTTTTATAGTCAAAACCTTTTCCAAAAGGACCTT

>Unigene63539_All - nr

GCTTACCTTCTTCTTTGCCAACTTCGGCCCTAATTCCACTACCTTTATAATACCTGCTGA

AATTTTCCCGGCTAGGTTAAGATCCACTTGTCATGGTATATCTGCTGCAACAGGAAAAGC

AGGGGCTATAATAGGAGCCTTTGGATTTGTTTATGCTTCCCAAGAGAAAGACCCTGCTAA

AAGAAGCGCTGGTTATCCAGCTGGTATTGGTAAAAAGAACTCTCTTATTGTTCTTGGTTG

CACTAATTT

>Unigene48391_All + nr

ACTACTACACTATTAGCTTTAATTTCTCCATATTTCTTTCTTTCTTTCCTCTTTCCACCT

CAAGTCATCTCCCCAACTTTAATTATAATTTTAAACAGGGCGTGTATGACAATTGTGAGC

TAAGAGGAAGAAAAATAATAACAAATAACAAAAAAGGTGAAAACACATCATGGCATCAAA

AACATCACAATTGGAAGTACTTAATGCACTTGATGTCGCAAAGACACAATTGTATCACTT

CACTGCAATTGTGATTGCCGGGATGGGGTTCTTTACAGATGCATATGATTTGTTCTGTAT

CTCCCTCCTAACAAAACTTCTAGGCCGCTTGTACTACACTGAACCTGGCGCAGACCATCC

AGGGGAT

>Unigene44133_All - nr

TCGTATTGGGTTCATTATAATGTACGCACTTACCTTCTTTTTTGCAAACTTTGGTCCTAA

TGCGACTACCTTTATAATTCCTGCGGAGATCTATCCAGCTAGGTTAAGGGCCACTTGCCA

CGGCATATCCGCTGCGACCGGAAAAGCGGGAGCTATAATAGGGGCTTTTGGATTTGTCTA

TGCTTCCCAAGAGAAAGACCCCGCCAAAAGAAGCGCTGGTTATCCCGCTGGTATTGGCAA

AAAGAACTCTCTTATTGTTCTTGGTTGCACTAATTTCCTGGGTATGTTGTGCACATTATT

GGTTCCTGAGTCAAAGGGTAAATCTCTTGAAGAATTAACTGGTGAGGCTGAGCTTGATGA

CCCTGAGGATGACCGCAGGGCTACTGCAGCTTGATAATTCATGCTGCTTCAATGTGATAA

CCACGCGCTCCTATATATTGCAACTAGTTGAATTGTTATGTTTATTTTTCATCAATCATA

AGGTGTGGATTTTTTATTATGAATCTTTGTACTATTGGATTGTATACCAATTGATCTATT

TTCTCATTTGTTATTAAATTATATATGGTTTGTGATATATAATAAAGTAAGCAAAGAAAT

ATACAGGAGCGCGTGGTTATC

>Unigene53055_All + nr

TATGCAAGAGGAAAAATTTAATGGTGTTGGATCTCTTATAATATATGTATTTGATTGTGT

AGGGCGTGTATGACAATTGTGAGCTAAGGGGAAGAAAAATAATAATAATAAAAGGTGAAA

ACACATCATGGCATCAAAAACATCACAATTGGAAGTACTTAATGCACTTGATGTCGCAAA

GACACAATTGTATCACTTCACTGCAATTGTGATTGCCGGGATGGGGTTCTTTACAGATGC

ATATGATTTGTTCTGTATCTCCCTCCTAACAAAACTTCTAGGCCGCTTGTACTACACTGA

ACCTGGCGCAGACCATCCAGGGGAACTGCCTATTAGTGTAAAATCTGCAGTTAGTGGAGT

TGCCCTCTGTGGGACTCTAGCTGGCCAAGTCTTTTTTGGTTGGCTCGGAGACAAGTTGGG

TCGGAAAAAGGTGTATGGTATTACCTTAGTTCTGATGGTTTGTTGTTCTATTGGCTCTGG

CCTTTCTTTTGGGAGCAGTGCCAAGGGGGTCATGACCACCATTTGCTTTTTTCGCTTTTG

GCTTGGGTTTGGTGTTGGAGGTGACTATCCCCTTTCTGCCACCATCATGTCTGAGTATGC

TAACAAAAGGACCCGTGGA

>Unigene44133_All - nr

TCGTATTGGGTTCATTATAATGTACGCACTTACCTTCTTTTTTGCAAACTTTGGTCCTAA

TGCGACTACCTTTATAATTCCTGCGGAGATCTATCCAGCTAGGTTAAGGGCCACTTGCCA

CGGCATATCCGCTGCGACCGGAAAAGCGGGAGCTATAATAGGGGCTTTTGGATTTGTCTA

TGCTTCCCAAGAGAAAGACCCCGCCAAAAGAAGCGCTGGTTATCCCGCTGGTATTGGCAA

AAAGAACTCTCTTATTGTTCTTGGTTGCACTAATTTCCTGGGTATGTTGTGCACATTATT

GGTTCCTGAGTCAAAGGGTAAATCTCTTGAAGAATTAACTGGTGAGGCTGAGCTTGATGA

CCCTGAGGATGACCGCAGGGCTACTGCAGCTTGATAATTCATGCTGCTTCAATGTGATAA

CCACGCGCTCCTATATATTGCAACTAGTTGAATTGTTATGTTTATTTTTCATCAATCATA

AGGTGTGGATTTTTTATTATGAATCTTTGTACTATTGGATTGTATACCAATTGATCTATT

TTCTCATTTGTTATTAAATTATATATGGTTTGTGATATATAATAAAGTAAGCAAAGAAAT

ATACAGGAGCGCGTGGTTATC

>Unigene125621_All + nr

CTCACTTCCACCCCCCATCTCCACTCCTGTTCATATCTCATACACAATGTCTGCCCAGGC

TCCTCCCACCTCCACCGGCAGCGTCAAGGTCGACGCTGTCAAGCAGCAGGTCCAGGCCGC

TGAGCCCCAGAAGCTTTCTGGTGTCGCTCTCTACTCTCGTTTCGCCTTCGCCGGTGCCGT

GTGCTGTTCCGTCACCCACGGTGGCCTCACCCCCGTCGATGTCGTCAAGACCCGCATCCA

GCTCGACCCCC

>Unigene61379_All - nr

GCTGCCAACAACCGCACCGCTGTCTACCTCGCCTCCTCCGCCTGCGCTGAGTTCCTTGCC

GACATTGCCCTCTGCCCTCTCGAGGCCACCCGTATCCGTCTCGTCTCCCAGCCTACCTTC

GCTACCGGTCTCGTGTCTGGTTTCTCCCGCATTGCCAAGGAGGAGGGTATTGGTGCTTTC

TACGGCGGTTTCGGCCCCATCCTCTTCAAGCAGATCCCCTACACCATGTCCAAGTTCGTC

GTCTACGAGAAGGTCGCTGAGGCCATCTACGCCAACTACGTCGACAAGGCCACTGCCTCT

GCTGGCATGAACACCACCGTCAACCTTGGTTCCGGTCTCATCGCCGGTTTCGCCGCCGCC

CTCGTCTCCCAGCCCGCCGACACCATGCTTTCCAAGATCAACAAGACCCAGGCTCTTCCT

GGTGAGGGTACCACCAGCCAG

>Unigene141416_All - nr

GAAGTCTAGTCATTCCTCTTTATTAATCCCTTCAAATCTATACTCTTCAGGTTCAACAAA

AAATGAAGTAATAATAATGAATAATAAAAAGAGAAAAAGTATAATGGTAGGAAGTCCAAT

TGAGAATAAGATGAAGATGTATTCTCCAACATTCTATGGTGCTTGTGTTACTGGTGGCAT

GCTTAGTTGTGGTCTCACTCATACTGCTATCACCCCTCTTGATCTCATCAAGTGTAACAT

CCAGATTGATCCAGCAAAGTACAAGAACATAAGCTCAGGATTTGGAGTCTTAATGAAAGA

GCAGGGGATTAAGGGGTTGTTTAAAGGTTGGGCACCAAAAATGTTTGGTTATAGTGCTCA

AGGAGCTTTTAAAATGGGAGGTTATGAGTTCTTCAAGAAGTATTATTCAGATATAGCTGG

TCCTGAAAATGCAGTCAAATACAAACCTCTAATAGTATTAGCTGGTTCTGCTTCTGCTGA

GCTGATTGCTGATGTTGCCCTTTGCCCCTTTGAGGCTGTTAAAGTTCGGGTCCAAACTCA

ACCCGGCTTTGCTCGTGGTTTGTTTGATGGTCTCCCTAAGATTGTCCGGTCCGAAGGTGT

TTCCGGATTGTACAGAGGATTAGCACCTCTTTGGGGCCGTCAGATTCCATATACAATGAT

GAAGTTTGCAACATTTGAGACTATAGTGGAGCAAATGTACAAGCATGTGATTCCAACACC

CA

>Unigene129694_All - nr

AACCTTGTCTCCTTCCTCAACAATTCTAAGGGTTCTTCTGTTTCTCATGCTGTGAAGAAG

CTGGGAGTGTGGGGATTATTTACACGTGGCCTTCCTCTGCGAATAGTCATGATTGGAACA

CTAACTGGTGCTCAATGGGGCATCTATGATGCTTTTAAAGTCATTGTTGGCCTACCAACT

ACTGGTGGAAGCTCTCCTACCCCTGAGGATAAAAATTGAACCAAGGCTATGGGCCGGG

>Unigene141415_All - nr

GAAGTCTAGTCATTCCTCTTTATTAATCCCTTCAAATCTATACTCTTCAGGTTCAACAAA

AAATGAAGTAATAATAATGAATAATAAAAAGAGAAAAAGTATAATGGTAGGAAGTCCAAT

TGAGAATAAGATGAAGATGTATTCTCCAACATTCTATGGTGCTTGTGTTACTGGTGGCAT

GCTTAGTTGTGGTCTCACTCATACTGCTATCACCCCTCTTGATCTCATCAAGTGTAACAT

CCAGGTTTTTTTTTTCTTTCTTTTTCCCATTTTATATACAATATACCCCCGTAACATATT

AATGATAACAATTTGAAATAAAGTAGAGGGTG

>Unigene129694_All - nr

AACCTTGTCTCCTTCCTCAACAATTCTAAGGGTTCTTCTGTTTCTCATGCTGTGAAGAAG

CTGGGAGTGTGGGGATTATTTACACGTGGCCTTCCTCTGCGAATAGTCATGATTGGAACA

CTAACTGGTGCTCAATGGGGCATCTATGATGCTTTTAAAGTCATTGTTGGCCTACCAACT

ACTGGTGGAAGCTCTCCTACCCCTGAGGATAAAAATTGAACCAAGGCTATGGGCCGGG

>Unigene141415_All - nr

GAAGTCTAGTCATTCCTCTTTATTAATCCCTTCAAATCTATACTCTTCAGGTTCAACAAA

AAATGAAGTAATAATAATGAATAATAAAAAGAGAAAAAGTATAATGGTAGGAAGTCCAAT

TGAGAATAAGATGAAGATGTATTCTCCAACATTCTATGGTGCTTGTGTTACTGGTGGCAT

GCTTAGTTGTGGTCTCACTCATACTGCTATCACCCCTCTTGATCTCATCAAGTGTAACAT

CCAGGTTTTTTTTTTCTTTCTTTTTCCCATTTTATATACAATATACCCCCGTAACATATT

AATGATAACAATTTGAAATAAAGTAGAGGGTG

>Unigene129694_All - nr

AACCTTGTCTCCTTCCTCAACAATTCTAAGGGTTCTTCTGTTTCTCATGCTGTGAAGAAG

CTGGGAGTGTGGGGATTATTTACACGTGGCCTTCCTCTGCGAATAGTCATGATTGGAACA

CTAACTGGTGCTCAATGGGGCATCTATGATGCTTTTAAAGTCATTGTTGGCCTACCAACT

ACTGGTGGAAGCTCTCCTACCCCTGAGGATAAAAATTGAACCAAGGCTATGGGCCGGG

>Unigene29837_All + nr

CTGTGTTTGCTGTCAAGTAGCTGGGGCGAGGACAATTTCGACTTGAGGCGAGTAGGAGTA

GTGTACCCAATCTTTCTCAACCCATACTAATTACCAGCCCATCTTCTCTCTCTCTCTCTC

CCTCTGTCCTCTACCCACTTCAGCAACTTACCATGAGCACTTCTCTTCCAACCTTCACCA

ATAATCTCCATTTTCGCACTACTTCCCCTTCTGCAAACCCTAATTTCCTCTTCTTCTCTT

CCCATAATTCTCTCACTTTCTCTAAATTCACTCAACATTCCATCAACAAACCTTCAATCC

TCCCTCCCTTCTTTGCTTCCCCGAATTCACCTCTTACTTTGAACTTCCGCCATGGATATT

CGACCTTTCTCGCCTCTAAAGCTAGGGTTTCTTCCGATGACTCTCGATTCTTCGCCGGAA

ATGAAGTTGGAGACAAAATGCAATCCCCCAGTTTTCTGGAATTTTTGACTTCTGAAAGAG

TTAAGGTTGTTATGATGCTTGCTCTAGCTCTTGGTCTCTGTAATGCTGATCGTGTTGTTA

TGTCTGTTGCTATTGTTCCTCTTTCGTCATCTCATGGCTGGAGTCGTGCCTTTGCTGGCG

TTGTTCAGTCATCTTTCTTGTGGGGGTATCTTGTATCACCTATAGCTGGTGGAGCTCTAG

TGGATTACTATGGTGGTAAAGCTGTCATGGACTGGGGTGTTGCTTTGTGGTCCTTTGCTA

CGTTTCTTACTCCTTAGGCAGCTGAAAAATCACTCTGGACTCTGCTTGCCGTGCGAGCTT

TGCTTGGCATTGCAGAAGGTGTAGCTCTTCCTTCAATGAACAACATGATATCAAAGAAAT

GGATAATGTTTCAATTGATTCAAAAACAAAATTGCTTCAGTGTCTACTCAGAAAATAAGT

CTGAAGAACATGGTAGGGTTGTGTGTTTGTTGAACCTATAAGTTTGTAACTATTGTCACC

CATCGTAATTTAGTTTGATGCTCATTAATTTGTCGATTGATTCACCCTTGTAATTATTTT

TTTTTTCCCTTAAATTAAGAGAATTTTTTTGTGGCTGATTCAATATATATCAGGTTACTT

GTATGGATTTACGTCATAGATTACAATTAGTTAATGTTTT

>Unigene46396_All + nr

TAGTGTTCCAGAGCGAAGCTCTCAGATATCTGAGGATGAACTAAATTACATACTGAAAAA

GAGGCAGCAGCCTCCACATACTCTGAAGGCTAAAGTAATCCCTCCTTTCAAGCGGTTGCT

CTCTAAGCTTCCAACTTGGTCACTTATCGTTGCTAATGCTATGCATAGTTGGGGGTTCTT

TGTTATTCTTTCATGGATGCCCTTGTATTTTAGCAGTGTATATAATGTCGATCTTAGACA

AGCAGCATGGTTTAGTGCTGTTCCATGGTGTATGATGGCAATTGTGGGATATTTTGGAGG

TGCTATTTCAGATATATTAATTCAGCGTGGTTTGAGTATCACTTTGACTCGAAAGATTAT

GCAGTCAATAGGGTTTATTGGTCCCGGCATTGCTCTTATTGGCCTGACTACAGCAGCTAA

CCCCTCGACAGCTTCTGCTTGGCTTACATTAGCTGTTGGACTAAAGTCATTCAGTCATTC

AGGTTTCCTTGTCAATATACAGGAAATTGCTCCACAATATACCGGAATATTACATGGGTT

GGCAAATACGGCTGGAACTTTTGCTGCGATTTTGGGGACAGTTGGGGCTGGTTATTTTGT

TCAACTGGTGGGATCTTTTAGTGGATTTTTATGGCTGACATCATTCTTATATTTTCTTGC

TGCCCTCTTCTGGAATCTATTTTCAACGGGGAAACTAGTAGACTTTGATGCATCCAGCA

**Sequencing of PCR products (8)**

> PCR product of Unigene pair 6

GCAACCAAGAACAATAAGAGAGTTCTTTTTGCCAATGCCAGCTGGATAACCAGCACTTCTTTTAGCAGGGTCTTTCTCTTGGGAAGCATAAACAAATCCAAAGGCTCCTATTATAGCCCCTGCTTTTCCTGTTGCAGCAGATATACCATGACAAGTGGATCTTAACCTAGCCGGGAAAATTTCAGCAGGTATTATAAAGGTAGTGGAATTAGGGCCGAAGTTGGCAAAGAAGAAGGTAAGCCCATACATTATGATGAACCCAATACGGTTCTCCCTCTTTTGCCAGTGGTTGTAAGGGAAGGCAACGCCAAACATGAATACGGTCATGAAAAAGAATCCCATCATCATGATTCTCCATCGTCCCATTATATCCATAAAGGCGACACTAAGCCAATATCCAGGAACAGTTCCACAAAGAGCAATGAGGCTTTGAGCCCTAGCTATCATATACACCTCCCTAATAGCATTCATTTCACCAGCCGGTGGTATCCAACCAACGGAACTAAAAAGGTCCTTTTGGAAAAGGTTTTGACTATAAAAAGCAACGTCTAGTAGGAACCACGTGCTTGTGGTTCCTAAAAGGCGAACTCCGTACCGACGAACAAATTCACGACTGAATAGCCCGAAATCGTTCTTGCCACTTTTTGAAATTTTCGCATAATTCTCCTCATCTGCGTCTATCTG

> PCR product of Unigene pair 7

CTTTCCACCTCAAGTCATCTCCCCAACTTTAATGATAATTTTAAACAGGGCGTGAATGACAATTGTGAGCTAAGGGGAAGAAAAATAATAATAATAAAAGGTGAAAACACATCATGGCATCAAAAACATCACAATTGGAAGTACTTAATGCACTTGATGTCGCAAAGACACAATTGTATCACTTCACTGCAATTGTGATTGCCGGGATGGGGTTCTTTACAGATGCATATGATTTGTTCTGTATCCCCCTCCTAACAAAACTTCTAGGCCGCTTGTACTACACTGAACCTGGCGCAGACCATCCAGGGGAACTGCCTATTAGTGTAAAATCTGCAGTTAGTGGAGTTGCCCTCTGTGGGACTCTAGCTGGCCAAGTCTTTTTTGGTTGGCTCGGAGACAAGTTGGGTCGGAAAAAGGTGTATGGTATTACCTTAGTTCTGATGGTTTGTTGTTCTATTGGCTCTGGCCTTTCTTTTGGGAGCAGTGCCAAGGGGGTCATGACAACTATTTGCTTTTTTCGCTTCTGGCTTGGGTTTGGTGTTGGAGGTGACTATCCCCTTTCTGCCACCATCATGTCTGAATATGCTAACAAAAGGACCCGTGGAAGCTTTATCGCCGCAGTCTTTGCTATGCAAgGTATGGGGATCTTGGCTGGGGGTGTTGTTTCCTTAATAGTTTCTTCTGCATTTGAGAGAAAGTTCCAACCTCCTTCCTTCAATGAAGATCCTGTGGCCTCCATGCCACCACAATCAGACTACATGTGGCGTATTGTCTTAATGTTTGGATCCATTCCTGCTGCCTTGACTTTCTACTGGCGTATAAAGATGCCAGAGACTGCAAGGTACACTGCCTTGGTAGCAAGAAATGCAAAGCAAGCAGCTGAGGACATGTCAAAGGTCCTGAAGGTTCAAATAGACGCAGATGAGGAGAAGTTTGAGACAATATCAAAAAATGATAGAAACCAATTTGGGCTATTCACCCGTGAATTTGCTCGTCGGTACGGCGTCCGTCTTTTAGGTACAACCAGCACTTGGTTCCTACTTGACGTTGCCTTTTATAGTCAAAACCTTTTCCAAAAGGACCTTTTTAGTTCTGTTGGGTGGATCCCACCTGCTGGTGAAATGAATGCTATTAGGGAGGTCTATATGATAGCTAGGGCTCAATCCCTTATTGCTCTTTGTGGAACCGTCCCTGGATATTGGTTTAGTGTCGCCTTCATGGATATAATGGGGCGATGGAGGATCATGATGATGGGATTCTTTTTCATGACCGTATTCATGTTTGGTGTTGGCATACCCTACGATCATTGGCAAAAGAAGGAAAACCGTATTGGGTTCATTATAATGTATGCACTTACCTTCTTCTTTGCCAACTTTGGTCCTAATGCGACTACCTTTATAATTCCTGCGGAGATCTATCCGGCTAGGTTAAGGGCCACTTGCCACGGCATATCCGCTGCGACTGGAAAAGCGGGAGCTATAATAGGGGCTTTTGGATTTGTCTATGCTTCCCAAGAGAAAGACCCCGCCAAAAGAAGCGCTGGTTATCCCGCTGGTATTGGCAAAAAGAACTCTCTTATTGTTCTTGGTTGCAC

> PCR product of Unigene pair 8

TCTGTATCTCCCTCCTAACAAAA

CTTCTAGGCCGCTTGTACTACACTGAACCTGGCGCAGACCATCCAGGGGA

ACTGCCTATTAGTGTAAAATCTGCAGTTAGTGGAGTTGCCCTCTGTGGGA

CTCTAGCTGGCCAAGTCTTTTTTGGTTGGCTCGGAGACAAGTTGGGTCGG

AAAAAGGTGTATGGTATTACCTTAGTTCTGATGGTTTGTTGTTCTATTGG

CTCTGGCCTTTCTTTTGGGAGCAGTGCCAAGGGGGTCATGACAACTATTT

GCTTTTTTCGCTTCTGGCTTGGGTTTGGTGTTGGAGGTGACTATCCCCTT

TCTGCCACCATCATGTCTGAATATGCTAACAAAAGGACCCGTGGAAGCTT

TATCGCCGCAGTCTTTGCTATGCAAGGTATGGGGATCTTGGCTGGGGGTG

TTGTTTCCTTAATAGTTTCTTCTGCATTTGAGAGAAAGTTCCAACCTCCT

TCCTTCAATGAAGATCCTGTGGCCTCCATGCCACCACAATCAGACTACAT

GTGGCGTATTGTCTTAATGTTTGGATCCATTCCTGCTGCCTTGACTTTCT

ACTGGCGTATAAAGATGCCAGAGACTGCAAGGTACACTGCCTTGGTAGCA

AGAAATGCAAAGCAAGCAGCTGAGGACATGTCAAAGGTCCTGAAGGTTCA

GATAGACGCAGATGAGGAGAAGTTTGAGACAATATCAAAAAATGATAGAA

ACCAATTTGGGCTATTCACCCGTGAATTTGCTCGTCGGTACGGCGTCCGT

CTTTTAGGTACAACCAGCACTTGGTTCCTACTTGACGTTGCCTTTTATAG

TCAAAACCTTTTCCAAAAGGACCTTTTTAGTTCTGTTGGGTGGATCCCAC

CTGCTGGTGAAATGAATGCTATTAGGGAGGTCTGTATGATAGCTAGGGCT

CAATCCCTTATTGCTCTTTGTGGAACCGTCCCTGGATATTGGTTTAGTGT

CGCCTTCATGGATATAATGGGGCGATGGAGGATCATGATGATGGGATTCT

TTTTCATGACCGTATTCATGTTTGGTGTTGGCATACCCTACGATCATTGG

CAAAAGAAGGAAAACCGTATTGGGTTCATTATAATGTATGCACTTACCTT

CTTCTTTGCCAACTTTGGTCCTAATGCGACTACCTTTATAATTCCTGCGG

AGATCTATCCGGCTAGGTTAAGGGCCACTTGCCACGGCATATCCGCTGCG

ACTGGAAAAGCGGGAGCTATAATAGGGGCTTTTGGATTTGTCTATGCTTC

CCAAGAGAAAGACCCCGCCAAAAGAAGCGCTGGTTATCCCGCTGGTATTG

GCAAAAAGAACTCTCTTATTGTTCTTGG

>PCR product of Unigene pair 10(up band)

CCTTGGTTCAATTTTTATCCTCAGGGGTAGGAGAGCTTCCACCAGTAGTTGGTAGGCCAACAATGACTTTAAAAGCATCATAGATGCCCCATTGAGCACCAGTTAGTGTTCCAATCATGACTATTCGCAGAGGAAGGCCACGTGTAAATAATCCCCACACTCCCAGCTTCTTCACAGCATGAGAAACAGAAGAACCCTTAGAATTGTTGAGGAAGGAGACAAGGTTATCAGCAGGGTGAGACACAGCAGCACAGAACACACCAGCAATATATCCTCCTACAAAGCTAACCCCCAACTGCACCTTCTTGCTACACTCTTTTTTGGGTGTTGGAATCACATGCTTGTACATTTGCTCCACAATAGTCTCAAATGTTGCAAACTTCATCATTGTATATGGAATCTGACGGCCCCAAAGAGGTGCTAATCCTCTGTACAATCCGGAAACACCTTCGGACCGGACGATCTTAGGGAGACCATCAGACAAACCACGAGCAAAACCGGGTTGAGTTTGGACCCGAACTTTAACAGCCTCAAAGGGGCAAAGGGCAACATCAGCAATCAGCTCAGCAGAAGCAGAACCAGCTAATACTATTAGAGGTTTGTATTTGACTGCATTTTCAGGACCAGCTATATCTGAATAATACTTCTTGAAGAACTCATAACCTCCCATTTTAAAAGCTCCTTGAGCACTATAACCAAACATTTTTGGTGCCCAACCTTTAAACAACCCCTTAATCCCCTGCTCTTTCATTAAGACTCCAAATCCTGAGCTTATGTTCTTGTACTTTGCTGGATCAATCTGGATGATACACTTGATGAGATCAAGAGGGGTGATAGCAG

>PCR product of Unigene pair 10(down band)

CCTTGGTTCAATTTTTATCCTGTGTTTTTCTGAAAATTCTTTCTTTTCTTTGTTACACCCTACTCTGTTTTTATTGATTATTTTGGGTTGGAATTGTTTTCAGATTTTAGTTTTCTGGGTTTTGATTATCATAATGATTCTTGTATCTGCTTATAATAAGTTAATAACTGTGTTTAAGTTTTATTTATGGGTTTTTGTGGTGCTTGAGGTTTAGCTCTTCACATTTTGTTGTGTCCTTTTTAGGTCTCTTTTGACTACAGGTGTAATGTAATGGTGTCAAATTATAACCCTTTTGCTTTGTGATAAATTATGAGCATTTGGTCCAAGAGATTGATATGGAAAATGATTCTGGCACTCCAAAATTTAATATTGGTTCTCCAATTGTGAGAGCATAATTTATCATACTCTACTCACAAGAGGGGTGATAGCAG

>PCR product of Unigene pair 11

AAAGTATAATGGTAGGAAGTCCAA

TTGAGAATAAGATGAAGATGTATTCTCCAACATTCTATGGTGCTTGTGTT

ACTGGTGGCATGCTTAGTTGTGGTCTCACTCATACTGCTATCACCCCTCT

TGATCTCATCAAGTGTAACATCCAGATTGATCCAGCAAAGTACAAGAACA

TAAGCTCAGGATTTGGAGTCTTAATGAAAGAGCAGGGGATTAAGGGGTTG

TTTAAAGGTTGGGCACCAAAAATGTTTGGTTATAGTGCTCAAGGAGCTTT

TAAAATGGGAGGTTATGAGTTCTTCAAGAAGTATTATTCAGATATAGCTG

GTCCTGAAAATGCAGTCAAATACAAACCTCTAATAGTATTAGCTGGTTCT

GCTTCTGCTGAGCTGATTGCTGATGTTGCCCTTTGCCCCTTTGAGGCTGT

TAAAGTTCGGGTCCAAACTCAACCCGGTTTTGCTCGTGGTTTGTCTGATG

GTCTCCCTAAGATCGTCCGGTCCGAAGGTGTTTCCGGATTGTACAGAGGA

TTAGCACCTCTTTGGGGCCGTCAGATTCCATATACAATGATGAAGTTTGC

AACATTTGAGACTATTGTGGAGCAAATGTACAAGCATGTGATTCCAACAC

CCAAAAAAGAGTGTAGCAAGAAGGTGCAGTTGGGGGTTAGCTTTGTAGGA

GGATATATTGCTGGTGTGTTCTGTGCTGCTGTGTCTCACCCTGCTGATAA

CCTTGTCTCCTTCCTCAACAATTCTAAGGGTTCTTCTGTTTCTCATGCTG

TGAAGAAGCTGGGAGTGTGGGGATTATTTACACGTGGCCTTCCTCTGCGA

ATAGTCATGATTGGAACACTAACTGGTGCTCAATGGGGCATCTATGATGC

TTTTAAAGTCATTGTTGGCCTACCAACTACTGGTGGAAGCTCTCCTACCC

CTGAGGATAAAAATTGAAC

>PCR product of Unigene pair 12(up band)

GTGTTGCTTTGTGGTCCTTGGTTTGAGGGGAGGATTGTTGGGGTTTGCAATTCAAGTCCCACATCGGAAAAATAACAGTGTTGTCTTGCTTATTAAAGTCCACCAACTCCATTAGTATGAGGCCTTTTGGGAAGGTGCCCAAAAAAAATCCGTACGGGCTATGCCCAAAGCAGACAATATCATACTAATGCCGGAGTCCAGGTGGTGTCGTGGGCCTAACAAACATATACAAAGTAAACCTTTGTATTTCATTTTTCCTATGATATTAACCTTTAAAACCCGATAACGAGTCAAGGTTAGTTTTAATGTCACCTTAATTATTGTGTTAACAACATCTTCAACATTATAGGCTTCCTAATTCACACCAAAATTTCCCTAATTAACGACATTACTTAGGCTATGTTAACCTTTGTATTTGATTTTTCCTATGTTATTAACCTTTAAAAACCAATAACAAGTCAAGGTTAGTTTTAATGTCACCTTAATTATTGTGTTAACAACATCTTCAACATTATAGGCTTCCTAATTCACATCAAAATTTCCCTAATTAACGCCATTACTTAGGCTATGTTTAGTTCACCTTATTTCAGGTACTTGTTATTTATTTTAGATCATATCAGATAAAAAAATAAGTTCAAATTAGATCGGAATAGAAAAAATAAGTTCAAATCAGGCTTGTAAATTTTATTTGATTTTTTCATACTTTAAATATCCTCAATTCCACAACAAAAGATATTATAAAAATCACATATTTGACAAATACTCATTAATACAAATAAAATAAGATCTCAATTGATATACTTTTTCCTTATGGATTAGTGTAACGCAGTCAATCAAATTAGACAATAAGTTCACATCAGAACTAATCATAAAATTAAGTTTAGTTCAAAAAAAAAAAGAAAAAAAAAGCTCCAACTAGATCAGATAATTTCATATCAGCAAAATCCATTGAACTAAACATAGCCTTAGTTAAGTGGGAATTAGTGAGACAGGAAATACTAGGACAAACTCGATCAAATTAAATGGAAGGAGTGTTCACAGCAAAATCCATTGAACTAAACATAGCCTTAGTTAAGTGGGAATTAGTGAGATAGGAAATAGTAGGACAAACTTGATCAAATTAAATGGAAGGAGTGTTCAAGGTTATGACTCATTCTAGTATCTAGGATACATTATCCAAAACGATGATAAATTAGATGAGGATTTTTCTCATAGGATCAAATTAGATTGATTAAAGTGGGAAGAAGGCTTCGATTGATTAAAAGTGGGAAGAAGATTCAAGTTTTCTTTGTGATCGGGGTATGCCACAAAGACTTGAGGGAAAATTCAACCGTACAGCTATTAGACCGACATTGCTATATGGCATGATTTGTAATTTCCTAGCTCCAACCTCTACATTTTGTTTCCAGAGGATTGATCAAAGAACATAATAAAGCTCTTTTCTATTGATCCATTAACTTCAAAGGCATTATTTAACATGTGATGAAGAAATGTGCAAGCAGAGAGTATACCATCAGGTGCCCCATTAAGTGGTTTTGCCTCAAATTAAAGAATGATATTTGAAAACTAAATTTCTTTGGTACTAAACTCGAACTTTCTTGTACAAAACATATGCATTCAACATAAGATTTCTGAATTGAAAGATAACTTCCTATGGAGTTCCAACACGGGTTATAATGCAGATCCATATCATTGACAGTGAGTTGATTTAAGACCAAAACTAACCACCCTGAAAATTTCACACCAAATGACCCAAACAAGCTACAATTTAACATAAATAATGGAGCCACAGACTGATATCATACTAAGGAACTGTATCAACAGCTTAAGCTATCAGATAAAATAGGTTTCATTTTTAACACCAACTAAGACATTATATAGATCTCTAACATTTTGAAATGAAAGACATACAACAGAGGTATATTTTACAGAATAGACCATAATGAGAGTGTTGCAAGATGACAAAGATTGATTTTAATTCAGCACTTAAAGACGGTAGGGACTGCCGTGCTGTTCCAGGGGGCCGGGCATTATATTGAGGTATATAAACTAAGAAGCATATTAAAGCTTTAATTACATCTCAAACTAAAAGAAAATCATAATGCAAGGTCAACCTAGAGGCAAACCTGTGCAGGACCACAAAGCAACAC

>PCR product of Unigene pair 12(down band)

GTGTTGCTTTGTGGTCCTCCGTTAGGAAGTCTTTATAATAAATTTGCAGTTAGACAATCAAGTTATTAATGACTGGGTAAAGTGAACTCTTATTATTCTGCTGGCTAAACATATATATCTATATATGCACAACAGCAAACCGTAACATGAAAAAATTATGACATTGCTAAAGCAATTGTTAGCTTCCCTTTTCTACAAACTCGAAATGGAATAAAATTCACATGTGCAACAGAGGCTTGCATAACAAATTCATAAGTACCTGGAGAGGATCTTGAAAATCATTCACAATGAATACATTTGCATCATCTGCTGATCTGGAAACAAATGTTTCAAAATAAAAAGTTAACACTGCTAGTACTAAGTAATACCAATTACGCACACATCACGGCCTCTTGTAAAGATTTGCGCCCTAAAAGCATCAGGATTTTTTAAATAAACACCTCATAATCTACCAAATTTTCTTAAAACCCTATGGCAACACTAACAACAAACATTTCTGTCAAATCATCCGCTAATGGATCCACCAAATAATGATTGGGGTAGATAAGTTCAAATTATAGCCAATAAAATCAAAAAGTTAAACTTTCTCATTACAGCAAATATATAATTTGCTAATTTAATTTCTCCCCAAAAGCAGAATTTTTAACTGAATTATTGGCTCTGTAAACCAATTTGTGGGATCCCAAGACCCACCAATATGAATTTTCAGGTTAATGTGGGATCCCAATAATCATAACTTTAAAAATTCATATTGGTGGGTATTGGGATCCCAATAAAAATTCATAAATTTAATTTCAGTTTTCAGACCACAAAGCAACAC

**Assembly sequences (3)**

> Assembly 6 (Unigene11489_All + PCR product + Unigene63539_All)

TTGGCGTATGCAGATGCCTGAGACCGCTAGGTACACTGCCCTAGTGGCAAAAAATGCAAA

ACAAGCAGCTGCCGACATGTCCAAAGTCCTCAAGGTTCAGATAGACGCAGATGAGGAGAA

TTATGCGAAAATTTCAAAAAGTGGCAAGAACGATTTCGGGCTATTCAGTCGTGAATTTGT

TCGTCGGTACGGAGTTCGCCTTTTAGGAACCACAAGCACGTGGTTCCTACTAGACGTTGC

TTTTTATAGTCAAAACCTTTTCCAAAAGGACCTTTTTAGTTCCGTTGGTTGGATACCACC

GGCTGGTGAAATGAATGCTATTAGGGAGGTGTATATGATAGCTAGGGCTCAAAGCCTCAT

TGCTCTTTGTGGAACTGTTCCTGGATATTGGCTTAGTGTCGCCTTTATGGATATAATGGG

ACGATGGAGAATCATGATGATGGGATTCTTTTTCATGACCGTATTCATGTTTGGCGTTGC

CTTCCCTTACAACCACTGGCAAAAGAGGGAGAACCGTATTGGGTTCATCATAATGTATGG

GCTTACCTTCTTCTTTGCCAACTTCGGCCCTAATTCCACTACCTTTATAATACCTGCTGA

AATTTTCCCGGCTAGGTTAAGATCCACTTGTCATGGTATATCTGCTGCAACAGGAAAAGC

AGGGGCTATAATAGGAGCCTTTGGATTTGTTTATGCTTCCCAAGAGAAAGACCCTGCTAA

AAGAAGCGCTGGTTATCCAGCTGGCATTGGCAAAAAGAACTCTCTTATTGTTCTTGGTTG

CACTAATTT

>Assembly 8 (Unigene53055_All + PCR product + Unigene44133_All)

TATGCAAGAGGAAAAATTTAATGGTGTTGGATCTCTTATAATATATGTATTTGATTGTGT

AGGGCGTGTATGACAATTGTGAGCTAAGGGGAAGAAAAATAATAATAATAAAAGGTGAAA

ACACATCATGGCATCAAAAACATCACAATTGGAAGTACTTAATGCACTTGATGTCGCAAA

GACACAATTGTATCACTTCACTGCAATTGTGATTGCCGGGATGGGGTTCTTTACAGATGC

ATATGATTTGTTCTGTATCTCCCTCCTAACAAAACTTCTAGGCCGCTTGTACTACACTGA

ACCTGGCGCAGACCATCCAGGGGAACTGCCTATTAGTGTAAAATCTGCAGTTAGTGGAGT

TGCCCTCTGTGGGACTCTAGCTGGCCAAGTCTTTTTTGGTTGGCTCGGAGACAAGTTGGG

TCGGAAAAAGGTGTATGGTATTACCTTAGTTCTGATGGTTTGTTGTTCTATTGGCTCTGG

CCTTTCTTTTGGGAGCAGTGCCAAGGGGGTCATGACAACCATTTGCTTTTTTCGCTTCTG

GCTTGGGTTTGGTGTTGGAGGTGACTATCCCCTTTCTGCCACCATCATGTCTGAATATGC

TAACAAAAGGACCCGTGGAAGCTTTATCGCCGCAGTCTTTGCTATGCAAGGTATGGGGAT

CTTGGCTGGGGGTGTTGTTTCCTTAATAGTTTCTTCTGCATTTGAGAGAAAGTTCCAACC

TCCTTCCTTCAATGAAGATCCTGTGGCCTCCATGCCACCACAATCAGACTACATGTGGCG

TATTGTCTTAATGTTTGGATCCATTCCTGCTGCCTTGACTTTCTACTGGCGTATAAAGAT

GCCAGAGACTGCAAGGTACACTGCCTTGGTAGCAAGAAATGCAAAGCAAGCAGCTGAGGA

CATGTCAAAGGTCCTGAAGGTTCAGATAGACGCAGATGAGGAGAAGTTTGAGACAATATC

AAAAAATGATAGAAACCAATTTGGGCTATTCACCCGTGAATTTGCTCGTCGGTACGGCGT

CCGTCTTTTAGGTACAACCAGCACTTGGTTCCTACTTGACGTTGCCTTTTATAGTCAAAA

CCTTTTCCAAAAGGACCTTTTTAGTTCTGTTGGGTGGATCCCACCTGCTGGTGAAATGAA

TGCTATTAGGGAGGTCTGTATGATAGCTAGGGCTCAATCCCTTATTGCTCTTTGTGGAAC

CGTCCCTGGATATTGGTTTAGTGTCGCCTTCATGGATATAATGGGGCGATGGAGGATCAT

GATGATGGGATTCTTTTTCATGACCGTATTCATGTTTGGTGTTGGCATACCCTACGATCA

TTGGCAAAAGAAGGAAAACCGTATTGGGTTCATTATAATGTACGCACTTACCTTCTTCTT

TGCAAACTTTGGTCCTAATGCGACTACCTTTATAATTCCTGCGGAGATCTATCCAGCTAG

GTTAAGGGCCACTTGCCACGGCATATCCGCTGCGACCGGAAAAGCGGGAGCTATAATAGG

GGCTTTTGGATTTGTCTATGCTTCCCAAGAGAAAGACCCCGCCAAAAGAAGCGCTGGTTA

TCCCGCTGGTATTGGCAAAAAGAACTCTCTTATTGTTCTTGGTTGCACTAATTTCCTGGG

TATGTTGTGCACATTATTGGTTCCTGAGTCAAAGGGTAAATCTCTTGAAGAATTAACTGG

TGAGGCTGAGCTTGATGACCCTGAGGATGACCGCAGGGCTACTGCAGCTTGATAATTCAT

GCTGCTTCAATGTGATAACCACGCGCTCCTATATATTGCAACTAGTTGAATTGTTATGTT

TATTTTTCATCAATCATAAGGTGTGGATTTTTTATTATGAATCTTTGTACTATTGGATTG

TATACCAATTGATCTATTTTCTCATTTGTTATTAAATTATATATGGTTTGTGATATATAA

TAAAGTAAGCAAAGAAATATACAGGAGCGCGTGGTTATC

>Assembly 10 (Unigene141416_All + PCR product + Unigene129694_All)

GAAGTCTAGTCATTCCTCTTTATTAATCCCTTCAAATCTATACTCTTCAGGTTCAACAAA

AAATGAAGTAATAATAATGAATAATAAAAAGAGAAAAAGTATAATGGTAGGAAGTCCAAT

TGAGAATAAGATGAAGATGTATTCTCCAACATTCTATGGTGCTTGTGTTACTGGTGGCAT

GCTTAGTTGTGGTCTCACTCATACTGCTATCACCCCTCTTGATCTCATCAAGTGTAACAT

CCAGATTGATCCAGCAAAGTACAAGAACATAAGCTCAGGATTTGGAGTCTTAATGAAAGA

GCAGGGGATTAAGGGGTTGTTTAAAGGTTGGGCACCAAAAATGTTTGGTTATAGTGCTCA

AGGAGCTTTTAAAATGGGAGGTTATGAGTTCTTCAAGAAGTATTATTCAGATATAGCTGG

TCCTGAAAATGCAGTCAAATACAAACCTCTAATAGTATTAGCTGGTTCTGCTTCTGCTGA

GCTGATTGCTGATGTTGCCCTTTGCCCCTTTGAGGCTGTTAAAGTTCGGGTCCAAACTCA

ACCCGGCTTTGCTCGTGGTTTGTCTGATGGTCTCCCTAAGATCGTCCGGTCCGAAGGTGT

TTCCGGATTGTACAGAGGATTAGCACCTCTTTGGGGCCGTCAGATTCCATATACAATGAT

GAAGTTTGCAACATTTGAGACTATAGTGGAGCAAATGTACAAGCATGTGATTCCAACACC

CAAAAAAGAGTGTAGCAAGAAGGTGCAGTTGGGGGTTAGCTTTGTAGGAGGATATATTGC

TGGTGTGTTCTGTGCTGCTGTGTCTCACCCTGCTGATAACCTTGTCTCCTTCCTCAACAA

TTCTAAGGGTTCTTCTGTTTCTCATGCTGTGAAGAAGCTGGGAGTGTGGGGATTATTTAC

ACGTGGCCTTCCTCTGCGAATAGTCATGATTGGAACACTAACTGGTGCTCAATGGGGCAT

CTATGATGCTTTTAAAGTCATTGTTGGCCTACCAACTACTGGTGGAAGCTCTCCTACCCC

TGAGGATAAAAATTGAACCAAGGCTATGGGCCGGG
